# Classification of human genomic regions based on experimentally determined binding sites of more than 100 transcription-related factors

**DOI:** 10.1186/gb-2012-13-9-r48

**Published:** 2012-09-05

**Authors:** Kevin Y Yip, Chao Cheng, Nitin Bhardwaj, James B Brown, Jing Leng, Anshul Kundaje, Joel Rozowsky, Ewan Birney, Peter Bickel, Michael Snyder, Mark Gerstein

**Affiliations:** 1 Program in Computational Biology and Bioinformatics, Yale University, 260 Whitney Avenue, New Haven, CT 06520, USA; 2 Department of Molecular Biophysics and Biochemistry, Yale University, 260 Whitney Avenue, New Haven, CT 06520, USA; 3 Department of Computer Science and Engineering, The Chinese University of Hong Kong, Shatin, New Territories, Hong Kong; 4 Hong Kong Bioinformatics Centre, The Chinese University of Hong Kong, Shatin, New Territories, Hong Kong; 5 CUHK-BGI Innovation Institute of Trans-omics, The Chinese University of Hong Kong, Shatin, New Territories, Hong Kong; 6 Department Statistics, University of California at Berkeley, 367 Evans Hall Berkeley, CA 94720, USA; 7 Department of Computer Science, Stanford University, 353 Serra Mall, Stanford, CA 94305, USA; 8 European Bioinformatics Institute (EMBL-EBI), Wellcome Trust Genome Campus, Hinxton, Cambridge, CB10 1SD, UK; 9 Department of Genetics, Stanford University School of Medicine, Mail Stop-5120, Stanford, CA 94305, USA; 10 Department of Computer Science, Yale University, 51 Prospect Street, New Haven, CT 06511, USA

## Abstract

**Background:**

Transcription factors function by binding different classes of regulatory elements. The Encyclopedia of DNA Elements (ENCODE) project has recently produced binding data for more than 100 transcription factors from about 500 ChIP-seq experiments in multiple cell types. While this large amount of data creates a valuable resource, it is nonetheless overwhelmingly complex and simultaneously incomplete since it covers only a small fraction of all human transcription factors.

**Results:**

As part of the consortium effort in providing a concise abstraction of the data for facilitating various types of downstream analyses, we constructed statistical models that capture the genomic features of three paired types of regions by machine-learning methods: firstly, regions with active or inactive binding; secondly, those with extremely high or low degrees of co-binding, termed HOT and LOT regions; and finally, regulatory modules proximal or distal to genes. From the distal regulatory modules, we developed computational pipelines to identify potential enhancers, many of which were validated experimentally. We further associated the predicted enhancers with potential target transcripts and the transcription factors involved. For HOT regions, we found a significant fraction of transcription factor binding without clear sequence motifs and showed that this observation could be related to strong DNA accessibility of these regions.

**Conclusions:**

Overall, the three pairs of regions exhibit intricate differences in chromosomal locations, chromatin features, factors that bind them, and cell-type specificity. Our machine learning approach enables us to identify features potentially general to all transcription factors, including those not included in the data.

## Background

Transcription factors (TFs) are proteins that bind specific DNA elements and regulate gene transcription. There are approximately 1,700 to 1,900 TFs in human, including about 1,400 manually curated sequence-specific TFs [1]. They bind different types of DNA elements, including promoters, enhancers, silencers, insulators and locus control regions [2]. While promoters are close to transcription start sites (TSSs), the other types of elements could be far away from the genes that they regulate, and there are no simple rules known to define their exact locations. For instance, enhancers can be as far as one mega base pairs (1 Mbp) from the target gene in eukaryotes [3], and can be both upstream and downstream of the promoter of the target gene [4].

One important step towards a thorough understanding of transcriptional regulation is to catalog all regulatory elements in a genome. There are databases for regulatory elements with experimental data [5-7]. The completeness of these databases has been limited by a small number of validation experiments performed relative to the expected number of regulatory elements, and a small amount of TF binding data available relative to the total number of TFs. There are also a lot of computational methods for predicting *cis*-regulatory modules, many of which are based on evolutionary conservation and binding motif densities and distributions [8,9]. Since these features are static information that does not take into account the dynamic environment of DNA, such as DNA methylation, nucleosome occupancy and histone modifications, these predictions usually have high false positive rates.

To systematically identify TF binding sites on a large scale, high-throughput methods such as chromatin immunoprecipitation followed by sequencing (ChIP-seq) [10,11] have been invented. With a goal to identify all functional elements in the human genome, the Encyclopedia of DNA Elements (ENCODE) project [12] has used high-throughput methods to produce a large amount of experimental data for studying TF binding sites. In the pilot phase, which aimed at studying 44 regions that sum up to about 1% of the human genome [13], the binding sites of 18 sequence-specific TFs and components of the general transcription machinery were identified using chromatin immunoprecipitation followed by microarray (ChIP-chip) [14,15], paired-end tag sequencing (ChIP-PET) [16], and sequence tag analysis of genomic enrichment (STAGE) [17]. Analysis of a subset of these data revealed non-uniform distribution of TF binding sites in the surveyed regions, statistical association of the binding sties with both TSSs and transcription end sites of known genes, and clustering of binding sites of different TFs [18].

With the success of the pilot phase, ENCODE has entered its production phase since 2007 to study DNA elements in the whole human genome. Both the scale and variety of experiments have been greatly increased [19,20]. In terms of protein-DNA binding, many ChIP-seq experiments have been performed to identify the binding sites of sequence-specific TFs, general TFs, and chromatin-related factors, which we will call transcription-related factors (TRFs) in general. About 500 ChIP-seq datasets have been produced, involving more than 100 different TRFs in more than 70 cell lines [20]. There are also matched expression data and chromatin features, such as histone modifications from ChIP-seq experiments, and DNA accessibility from DNase I hypersensitivity analysis [21,22] and formaldehyde-assisted isolation of regulatory elements (FAIRE) [23], making the dataset a valuable resource for studying transcriptional regulation.

Having this large amount of data available notwithstanding, it is still non-trivial to identify all regulatory elements and provide useful annotations for them due to two major reasons. First, the fraction of TRFs included in the experiments is still small compared to the total number of TRFs in human. For instance, if a regulatory element is only bound by TRFs not covered by these experiments, it cannot be identified simply by cataloging all the observed TRF binding sites. Instead, it is necessary to model each type of regulatory element by some general features that are available for the whole genome, and use these features to extend the search of the elements to regions not covered by the experiments.

Second, the overwhelming amount of data makes it difficult to extract useful information. Processing hundreds of genome-scale data files requires a lot of computational resources even for simple analysis tasks, not to mention the complexity in cross-referencing other types of related data, such as gene expression and histone modifications. Statistical significance of observations is also difficult to evaluate due to non-uniform distribution of genomic elements and complex dependency structures within a single dataset and between different datasets.

Here we report our work in using statistical methods to learn general properties of different types of genomic regions defined by TRF binding. We also describe the application of the learned models in locating all occurrences of these types of regions in the whole human genome in different cell types, including locations with no direct experimental binding data. Our main goal is to provide a concise and accessible summary of the large amount of data in the form of several types of regions with clear interpretations, to facilitate various kinds of downstream analyses.

Specifically, we report our identification of six different types of genomic regions that can be grouped into three pairs: regions with active/inactive binding; regulatory modules proximal to promoters/distal to genes; and regions with extremely high/low degrees of co-occurrence of binding by factors that do not usually co-associate. We discuss the chromosomal locations of these regions, their cell-type specificity, chromatin features and different sets of TRFs that bind them, and show that a variety of properties of our called regions are in strong agreement with prior knowledge of TRF binding.

To further explore functional aspects of the identified regions, we report our work in predicting enhancers from the distal regulatory modules and validating their activities by reporter assays. We also link distal regulatory modules to potential target genes and identify the TRFs involved. Finally, we suggest a potential relationship between non-sequence-specific TRF binding and DNase hypersensitivity at regions with high co-occurrence of TRF binding. All these whole-genome analyses would have been difficult to carry out without the large cohort of data produced by ENCODE.

Related ideas for identifying different types of regions in the whole genome have been proposed, both by groups within ENCODE and by other groups. One approach is to use one or a few previously known features to define particular region types, such as using DNase I hypersensitivity and some specific histone marks in identifying enhancers. In comparison, our approach identifies feature patterns directly from data using a machine learning framework, which has the potential to discover novel features for specific region types. Another related idea is to segment the genome in an 'unsupervised' fashion, that is, to group regions based on observed data alone without any predefined region types. This approach is most suitable for exploring new region types. A big challenge of this approach is to interpret the resulting segments. In the current work we focus on the six types of regions described, and take on a 'supervised' approach whenever possible, that is, to learn general properties of a region type using known examples. When there are sufficient examples, the supervised approach is usually preferred in identifying members of well-defined classes.

## Results

### Identification of six types of genomic regions based on TRF binding data

We selected five ENCODE cell lines that have the largest numbers of TRFs with binding sites assayed by ChIP-seq (Table S1 in Additional file 1). In total, 117 TRFs are included in the ENCODE datasets from the five cell lines. The data files were processed by the ENCODE pipeline [24], which includes signal quality and reproducibility tests by comparing data from replicate experiments, a uniformly applied procedure for calling binding peaks (using PeakSeq [25] for our selected subset of data), and the removal of problematic regions due to issues such as repeats and sequences with low mappability.

**Figure 1:**
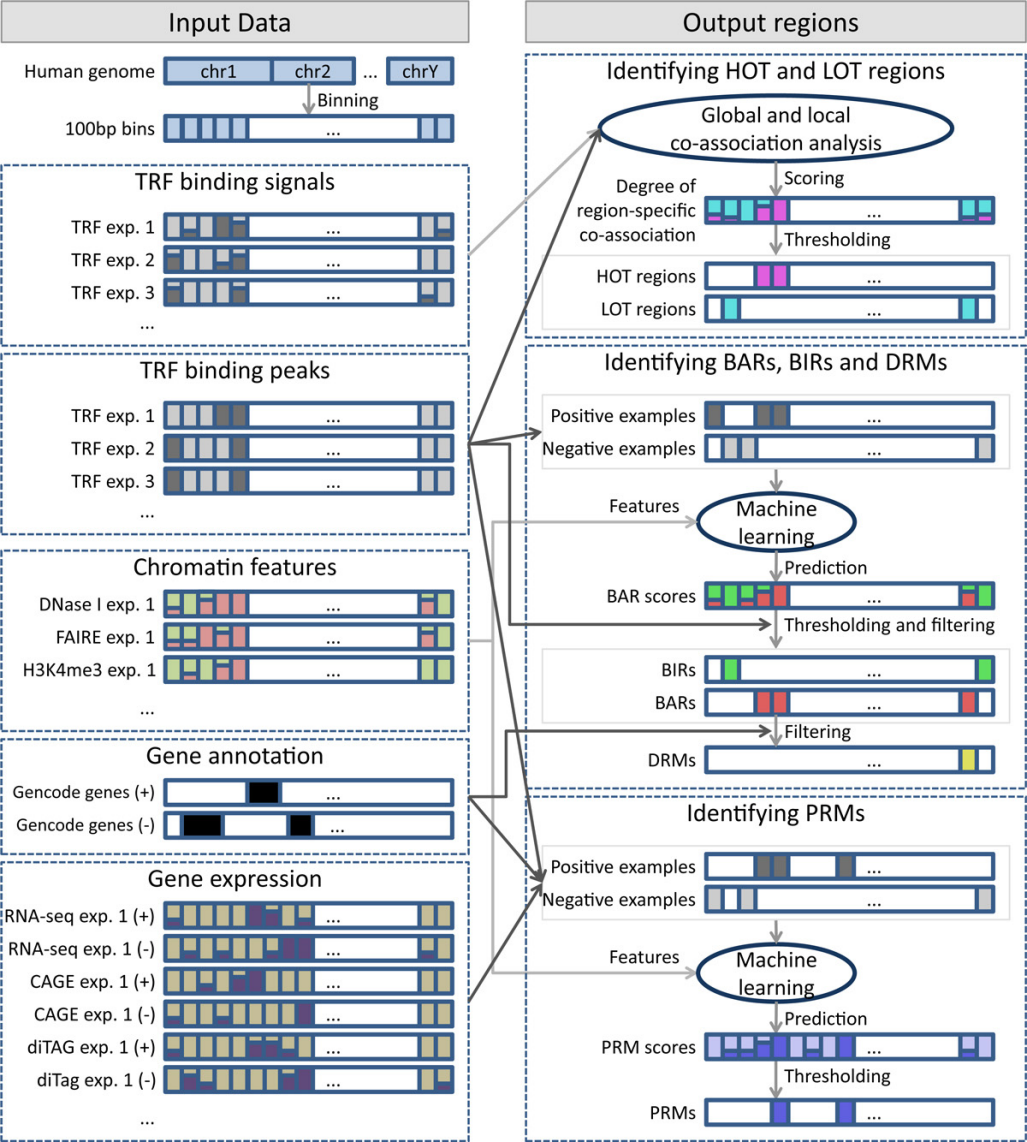
**Overview of the pipeline for identifying the six types of regions for one cell line**. The left side shows the input data involved. The right side shows how these datasets were used to identify the regions. The same pipeline was applied to five different cell lines. See Materials and methods for details. The color scheme for the six regions is used in all figures and supplementary figures of the paper. CAGE, cap-analysis of gene expression; exp., experiment.

For each of the five cell lines, we used the cell-line-specific TRF binding data to learn patterns in chromatin features and gene expression levels using machine learning methods. We then used the learned models to define six different types of genomic regions that form three pairs: 1) binding active regions (BARs) and binding inactive regions (BIRs); 2) promoter-proximal regulatory modules (PRMs) and gene-distal regulatory modules (DRMs); and 3) high occupancy of TRF (HOT) regions, and low occupancy of TRF (LOT) regions (Figure 1). In each pair, the two region types are mutually exclusive. On the other hand, region types from different pairs may overlap. For instance, DRMs are subsets of BARs, while some HOT regions overlap with PRMs and DRMs. Each of the six types of regions, however, exhibits some unique properties and we will discuss the six types separately. With the use of cell-line-specific data, we aimed at identifying regions that reflect the internal states of the particular cell types. For PRMs and DRMs, for example, our goal was to identify modules that have active regulatory roles in the particular cell line from which they were called, instead of modules that are only potentially active in some unknown cell types [26].

#### Binding active regions and binding inactive regions

We first identified broad genomic regions that TRFs tend to bind, which we call binding active regions (BARs). One simple way to define BARs is to collect all regions covered by the binding peaks of the TRFs in our dataset, which are regions with the strongest binding signals compared to the local genomic backgrounds. However, while we are using one of the largest sets of ChIP-seq data currently available, it contains only a small portion of the estimated 1,700 to 1,900 human TFs [1]. We therefore took the regions covered by the TRF binding peaks as examples to learn a statistical model based on the observed chromatin features of these regions for each cell line using data produced by ENCODE (Materials and methods). We then applied the model to score all regions in the whole human genome. Cross-validation results show that our learned models can separate regions covered by TRF binding peaks from other random regions well (Figures S1 and Figure S2 in Additional file 2). Since some of the selected random regions may actually be bound by TRFs not in our dataset, we do not expect 100% accuracy, and the observed accuracy values are sufficiently high to indicate that our models have captured some general chromatin properties of regions with active binding. We then defined a cutoff threshold to define BARs for each cell line as regions with a score higher than it (Materials and methods).

To contrast with BARs, we also defined BIRs as regions that have low BAR scores and are not covered by any binding peaks of the TRFs in our dataset.

#### Promoter-proximal regulatory modules and gene-distal regulatory modules

Among the TRF binding sites, one subset of particular interest comprises those close to the TSSs of active genes, as they are likely actively involved in the regulation of these genes in the corresponding cell lines. Depending on the distance from a TSS, these regions may contain core promoters and proximal promoter elements [2]. We call these regions promoter-proximal regulatory modules (PRMs) in general. To define PRMs, instead of using an arbitrary distance threshold from TSSs, we determined distance cutoffs according to chromatin feature patterns using a machine learning framework. Specifically, for each cell line, we took TSSs of genes expressed in the cell line as positive examples, and random non-TRF binding sites and distal TRF binding sites as negative examples (Materials and methods). Expression of TSSs was determined by ENCODE data from cap-analysis of gene expression (CAGE) [27], paired-end diTag (PET) [28], and RNA sequencing (RNA-seq) [29,30]. Based on the examples, a discriminative model was learned using chromatin features and TRF binding data of the cell line as explanatory variables. The resulting models separated positive and negative examples well in all cell lines (Figures S3 and S4 in Additional file 2). Finally we used the learned models to give PRM scores to all regions in the whole genome. Since in this case we have a relatively complete set of positive examples from annotated genes, we used a more stringent threshold to call PRMs (Materials and methods).

In contrast to PRMs, there are also regulatory modules that are more distal to promoters. For example, enhancers are frequently thousands of bases pairs upstream or downstream of a promoter, and they can be within an intron of a gene [2]. To study properties unique to this type of DNA element, we focused on BARs at least 10 kbp from any annotated coding and non-coding transcript (Materials and methods) and removed from this list any identified PRMs, to eliminate properties superimposed from annotated and potentially unannotated genes.

#### High occupancy of TRFs and low occupancy of TRFs regions

In addition to binding potential and relative distance from genic features, TRF binding regions can also be classified by the likelihood of co-occurrence of TRF binding sites. In separate studies we have observed widespread co-occurrence of binding sites of different TRFs [20,31]. An extreme case is the binding of many TRFs at the same narrow regions on the scale of around a hundred base pairs. While it is physically impossible to have many TRFs binding a small site at this scale at the same time in a single cell, different TRFs can simultaneously bind to the same site in a population of cells and be detected by a single ChIP-seq experiment. We were particularly interested in regions bound by many TRFs that do not frequently co-associate globally in the whole genome. We call this kind of event region-specific TRF co-occurrence. For instance, since members of the c-Jun and c-Fos families dimerize to form the AP-1 transcription factor [32], their binding sites co-occur globally [20] and this kind of co-occurrence is not regarded as region-specific TRF co-occurrence.

We derived a method to compute the degree of region-specific co-occurrence of TRF binding sites, which takes into account both the binding signals and global co-occurrence of TRFs (Materials and methods). Basically, binding peaks with stronger, more reliable binding signals are weighted more, while sets of TRFs that frequently co-occur in the whole genome are group-wise down-weighted.

We found that the degree of region-specific TRF co-occurrence forms a smooth distribution with no obvious peaks except at around zero due to regions with no TRF binding (Figure S5 in Additional file 2). We extracted the most extreme cases and defined HOT regions and LOT regions as the regions with the highest and lowest (but non-zero) degrees, respectively (Materials and methods). Genome-wide analyses of HOT regions have been performed before in *Caenorhabditis elegans *[33] and *Drosophila *[34]. In the current work we developed an improved computational method to study these regions at the genome scale in human.

### Genomic locations of the six types of regions

**Figure 2:**
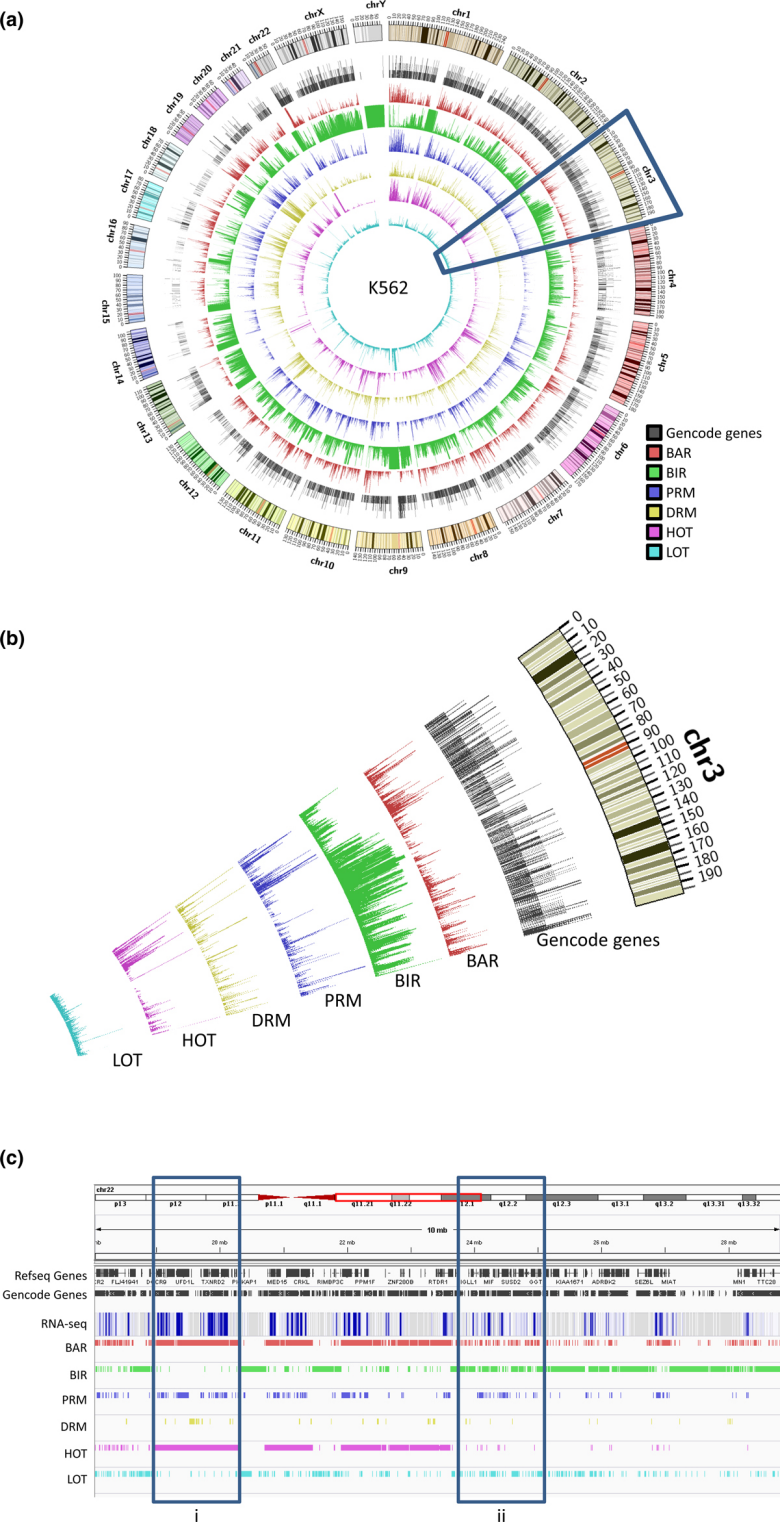
**Distribution of the six types of regions in the genome in K562**. **(a) **Densities of the regions in the whole genome, defined as the running fractions of bases covered by the regions. The tracks are, respectively, from outermost to innermost, the ideogram for the human karyotype (genome build hg19), Gencode version 7 level 1 and level 2 genes, BARs, BIRs, PRMs, DRMs, HOT regions and LOT regions. The tracks are scaled separately to show density fluctuations. The highlighted segment corresponds to the area in (b). **(b) **Zoom-in of chromosome 3 to show the correlated fluctuations of the different types of regions. **(c) **Locations of the six types of regions at the beginning of the q-arm of chromosome 22 in K562. Due to the high density of genes, only a subset of the gene names is shown. Expression values were measured by long poly-A+ RNA-seq of whole-cell RNA extract. A darker color indicates a higher average expression level in the local region. Box i marks a broad area with significant active TF binding and co-binding. Box ii marks an area with many small interspersed active and inactive TF binding regions.

The six types of regions identified by our computational methods occupy from about 15.5 Mbp (PRMs in H1-hESC, equivalent to 0.50% of the human genome) to 1.39 Gbp (BIRs in GM12878, equivalent to 45% of the human genome) in the different cell lines (Table 1). At a global scale, their locations are highly non-uniform and inter-related (Figures 2a; Figure S6 in Additional file 2; visualization by Circos [35]). BARs are correlated with gene density (Figure 2b). PRMs and DRMs are, by definition, distributed according to gene locations. For HOT regions, about 70 to 80% of them are within 10 kbp of annotated coding and non-coding genes, while the remainder are at intergenic regions (Table 1). In contrast, only about half of the LOT regions are close to or overlap genes, and the other half are within intergenic regions.

**Table 1:** Total sizes of the six types of genomic regions derived from transcription factor binding data in the five cell lines

Region type	GM12878	H1-hESC	HeLa-S3	Hep-G2	K562
Binding active regions (BARs)	109 Mbp (3.5%)	78.8 Mbp (2.6%)	93.6 Mbp (3.0%)	88.8 Mbp (2.9%)	98.7 Mbp (3.2%)
Binding inactive regions (BIRs)	1,390 Mbp (45%)	1,200 Mbp (39%)	1,330 Mbp (43%)	401 Mbp (13%)	1,010 Mbp (33%)
Promoter-proximal regulatory modules (PRMs)	25.4 Mbp (0.82%)	15.5 Mbp (0.50%)	20.7 Mbp (0.67%)	17.1 Mbp (0.55%)	24.3 Mbp (0.79%)
Gene-distal regulatory modules (DRMs)	24.6 Mbp (0.80%)	18.2 Mbp (0.59%)	25.3 Mbp (0.82%)	21.1 Mbp (0.68%)	22.0 Mbp (0.71%)
High occupancy of TF (HOT) regions (whole genome)	25.9 Mbp (0.84%)	26.4 Mbp (0.86%)	25.6 Mbp (0.83%)	26.3 Mbp (0.85%)	26.6 Mbp (0.86%)
High occupancy of TF (HOT) regions (intergenic regions only)	5.82 Mbp (0.19%)	6.43 Mbp (0.21%)	6.70 Mbp (0.22%)	6.74 Mbp (0.22%)	5.18 Mbp (0.17%)
Low occupancy of TF (LOT) regions (whole genome)	24.7 Mbp (0.80%)	23.5 Mbp (0.76%)	22.8 Mbp (0.74%)	23.8 Mbp (0.77%)	24.2 Mbp (0.78%)
Low occupancy of TF (LOT) regions (intergenic regions only)	10.7 Mbp (0.35%)	11.0 Mbp (0.36%)	10.1 Mbp (0.33%)	10.7 Mbp (0.35%)	12.4 Mbp (0.40%)

Numbers in parentheses are the percentages of the whole human genome covered by the regions. Intergenic regions are defined as regions at least 10 kbp from any level 1 or level 2 gene defined in Gencode version 7.

Figure 2c shows the relative locations of the six types of regions in an example area at the beginning of the q-arm of chromosome 22 in K562 (visualization by IGV [36]). There are large segments of DNA covered by BIRs with low gene activities as measured by RNA-seq. BARs are, in general, distributed according to gene locations, but there are two major subtypes. One subtype corresponds to broad areas with extensive TRF binding and co-binding, as indicated by continuous BAR and HOT regions, respectively (Figure 2c, box i). The other subtype involves regions with interspersed active and inactive TRF binding, where only a small fraction of the PRMs and DRMs intersect with HOT regions (Figure 2c, box ii). As discussed below, the former likely corresponds to general open chromatin regions with potential 'motifless' binding, while the latter involves more sequence-specific binding.

**Figure 3:**
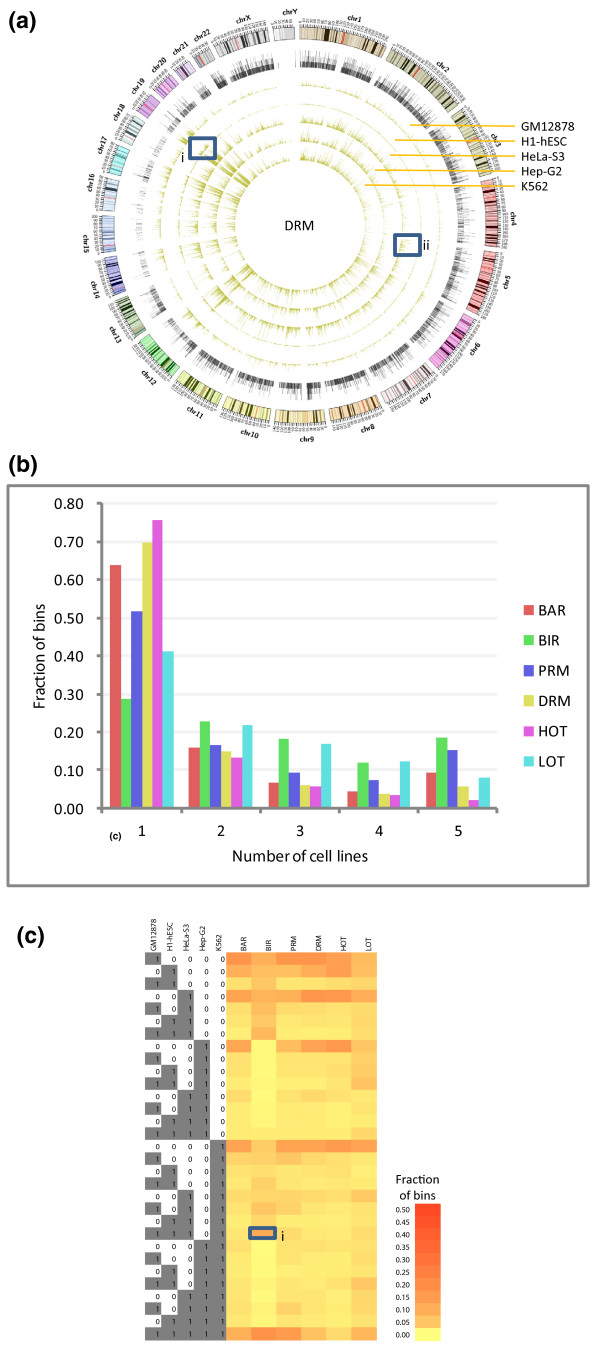
**Distribution of the DRMs in the five different cell lines**. **(a) **Densities of the regions in the whole genome, defined as the running fractions of bases covered by the regions. The tracks are, respectively, from the outermost to the innermost, the ideogram for the human karyotype (genome build hg19), Gencode version 7 level 1 and level 2 genes, and regions in GM12878, H1-hESC, HeLa-S3, Hep-G2 and K562. The five innermost tracks are all in the same scale. Box i shows an area with an exceptionally high density of DRMs on chromosome 19 in the h1-hESC line. Box ii shows an area with exceptionally high density of DRMs on chromosome 5 in HeLa-S3 cells. **(b) **Fraction of bins covered by the six types of regions shared by different numbers of cell lines. **(c) **Fraction of bins covered by the six types of regions shared by the 31 possible combinations of the 5 cell lines. Box i marks the high fraction of BIR bins shared by cell lines GM12878, H1-hESC, HeLa-S3, and K562.

In general, each of the six types of regions shows a high level of consistency across different cell lines (Figure 3a; Figure S7 in Additional file 2), despite the fact that the regions in different cell lines were called independently using datasets from different sets of TRFs. For example, while no constraints were placed as to where the BARs should be called in the whole genome, their resulting genomic distributions in the different cell lines are highly similar (Figure S7A in Additional file 2).

Amid the general consistency, some subtle cell-type-specific patterns are also observed. At the genome scale, H1-hESC is found to differ most from the other cell lines by having much lower average densities of all regions except BIRs, which highlights the drastic difference between embryonic stem cells and differentiated cells. Among the different chromosomes, there is a higher density of BARs on chromosome 19 in H1-hESC, many of which are DRMs (Figure 3a, box I; Figure S7A in Additional file 2). The high density of BARs is consistent with both the intrinsic high gene density of chromosome 19 [37], and the highest over-representation of genes expressed on this chromosome in human embryonic stem cells, as previously observed [38].

Some local regions also exhibit cell line specificity. For example, the p-arm of chromosome 5 has a much higher density of DRMs in HeLa-S3 than the other cell lines (Figure 3a, box ii). This region also has a high degree of region-specific co-occurrence of TRF binding (Figure S7E in Additional file 2), which is not found in the other four cell lines. There were previous reports that HeLa cells contain three to five copies of isochromosome 5p [39], which may have caused stronger binding and open chromatin signals.

We then systematically computed the overlap of each type of region in the different cell lines. Overall, BIRs show the highest level of consistency, with 18% of all BIR bins identified from the different cell lines commonly shared by all five cell lines, and only 29% unique to one particular cell line (Figure 3b). In contrast, active regions show higher levels of cell-line specificity. For example, 76% of the indentified HOT regions are specific to only one cell line, which means, on average, each cell line contributes about 15% unique regions to the whole set of HOT regions.

We also examined all combinations of the five cell lines, and found that Hep-G2 missed a substantial set of BIRs present in the other cell lines (Figure 3c, box i), which can also be observed from a density plot (Figure S7B in Additional file 2). In general, no two cell lines appear to be particularly more similar to each other than to other cell lines in terms of the six types of regions.

### Chromatin features of the six types of regions

We then studied various chromatin features of the six types of regions, including open chromatin signatures and histone modifications. The set of histone modifications from the ENCODE experiments consists of both active (for example, histone 3 lysine 4 tri-methylation (H3K4me3)) and repressive (for example, H3K9me3) marks, as well as marks that are usually found at promoters (for example, H3K4me3), gene bodies (for example, H3K36me3) and distal elements (for example, H3K4me1) (Table S2 in Additional file 1) [40].

For each combination of cell line, region type and chromatin feature, we collected the signal values of the feature at all regions of that type in the cell line to form a distribution (Materials and methods). We then compared these distributions of different types of regions. The full set of distributions is shown in Figure S8 in Additional file 2 using box-and-whisker plots (visualization by JFreeChart [41]).

**Figure 4:**
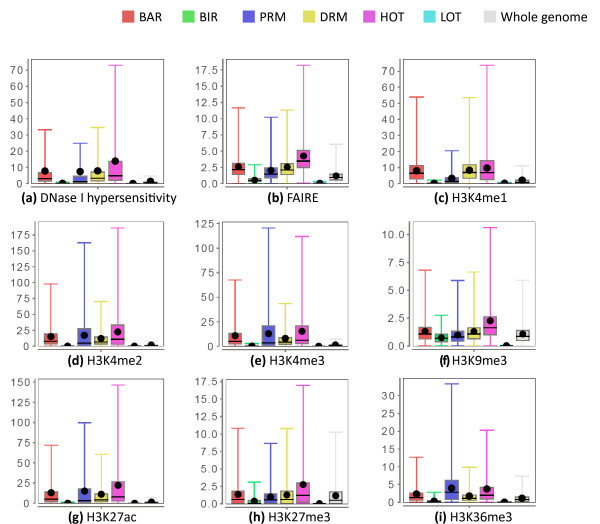
**Chromatin features of the six types of regions in K562**. **(a) **DNase I hypersensitivity from the dataset Uw.OpenChrom.K562.Dnase.Na (compare Figure S8E in Additional file 2). **(b) **FAIRE signals from the dataset Unc.OpenChrom.K562.Faire.Na. **(c) **H3K4me1 signals from the dataset Broad.Histone.K562.H3K4me1.Std. **(d) **H3K4me2 signals from the dataset Broad.Histone.K562.H3K4me2.Std. **(e) **H3K4me3 signals from the dataset Broad.Histone.K562.H3K4me3.Std. **(f) **H3K9me3 signals from the dataset Broad.Histone.K562.H3k9me3.Std. **(g) **H3K27ac signals from the dataset Broad.Histone.K562.H3k27ac.Std. **(h) **H3K27me3 signals from the dataset Uw.Histone.K562.H3k27me3.Std. **(i) **H3K36me3 signals from the dataset Uw.Histone.K562.H3k36me3.Std. Each dataset ID has the format <Data source>.<Experiment type>.<Cell line>.<Open chromatin method/histone modification/TF>.<Experiment details>. The dot in each box-and-whisker plot is the average value. Some outlier values are not shown. See Materials and methods for details.

Figure 4 shows some of the characteristic chromatin features of the different regions. For each type of data, we have picked a particular dataset from the K562 cell line for illustration, but the general trends are also observed in other datasets in K562 and in other cell lines.

BARs, PRMs and DRMs have strong open chromatin signals (Figure 4a,b), consistent with their expected roles as active gene regulatory elements [21,23,42]. PRMs have stronger H3K4me3 signals and DRMs have stronger H3K4me1 signals (Figure 4c,e), which are expected since H3K4me3 is a signature of active promoters while H3K4me1 is an indicator of enhancers [43]. Both PRMs and DRMs have enriched H3K4me2 signals over the whole genome, which is also consistent with previous observations [40]. PRMs have stronger H3K36me3 and H3K79me2 signals (Figure S8 in Additional file 2) than DRMs. These histone marks are found in transcribed regions [44-46], and are thus good features for distinguishing between regulatory elements that are close to and those that are far away from transcribed genes.

We notice that histone 3 lysine 27 acetlylation (H3K27ac), which is expected to be enriched at enhancers [40], has much stronger signals at both PRMs and DRMs than the genomic background. However, the enrichment is slightly stronger at PRMs than DRMs. It is likely caused by a combination of reasons. First, our DRMs consist of all kinds of distal regulatory elements, which may include non-enhancers such as insulators and silencers that do not have strong H3K27ac signals. Second, some enhancers are within 10 kbp of a gene, which are not included in the DRM set based on our current definition. Third, some of our DRMs may be inactive or poised enhancers, which have weaker H3K27ac signals [47], although they still have strong H3K4me1 signals in general. Finally, clear H3K27ac signals have also been previously reported at promoters in four of the five cell lines we are considering, in the ENCODE pilot regions based on ChIP-chip data [48], which suggests that this histone modification may also have a functional role at promoters.

One slightly surprising result is that, compared to the genomic background, PRMs and DRMs are not depleted of H3K9me3 signals, which were thought to be repressive marks. Previous studies reported the presence of H3K9me3 at transcribed regions of active genes [49,50]. Our results suggest the possibility that some active regulatory elements may have both classical active marks (such as H3K4me3) and H3K9me3 simultaneously. When two different amino acid residues (H3K4 and H3K9) are involved, it is also possible for the same histone protein to have both kinds of marks. Since PRMs are highly associated with transcribed genes, we hypothesize that having some strong active marks may be sufficient to counter the effects of repressive marks.

Both BIRs and LOT regions are depleted of most of the histone modifications relative to the whole genome. BIRs are slightly more enriched for open chromatin and repressive (H3K9me3 and H3K27me3) signals, which suggest that BIRs are more accessible to TRFs but transcriptional activities are repressed, while LOT regions in general have low DNA accessibility.

Comparing with the other five types of regions, HOT regions are characterized by strong enrichment for almost all kinds of open chromatin and histone modification signals. The enrichment over other types of regions is particularly strong for open chromatin signals, indicating high accessibility of DNA at these regions.

### TRFs that bind the six types of regions

**Figure 5:**
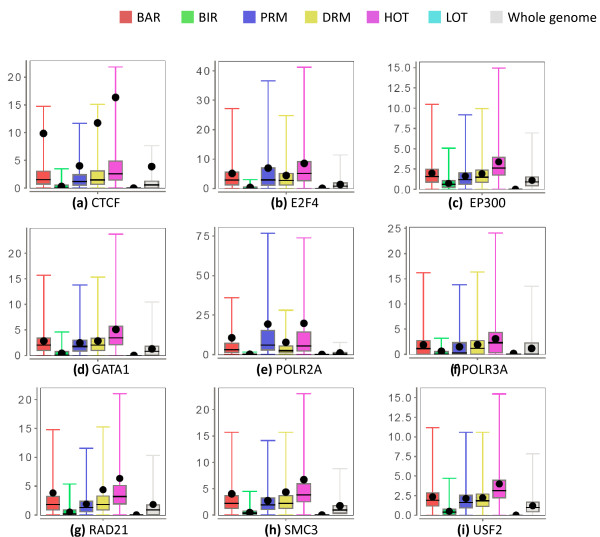
**TRF binding signals of the six types of regions in K562**. **(a) **CTCF signals from the dataset Uta.Tfbs.K562.Ctcf.Na. **(b) **E2F4 signals from the datasets Sydh.Tfbs.K562.E2f4.Ucd. **(c) **EP300 signals from the dataset Sydh.Tfbs.K562.P300f4.Iggrab. **(d) **GATA1 signals from the dataset Sydh.Tfbs.K562.Gata1.Ucd. **(e) **POLR2A signals from the dataset Sydh.Tfbs.K562.Pol2.Std. **(f) **POLR3G signals from the dataset Sydh.Tfbs.K562.Pol3.Std. **(g) **RAD21 signals from the dataset Sydh.Tfbs.K562.Rad21.Std. **(h) **SMC3 signals from the dataset Sydh.Tfbs.K562.Smc3ab9263.Iggrab. **(i) **USF2 signals from the dataset Sydh.Tfbs.K562.Usf2.Std. Each dataset ID has the format <Data source>.<Experiment type>.<Cell line>.<Open chromatin method/histone modification/TF>.<Experiment details>. The dot in each box-and-whisker plot is the average value. Some outlier values are not shown. See Materials and methods for details.

We further studied the TRFs that bind the different types of regions by examining their binding signals (Materials and methods). The whole set of results is shown in Figure S8 in Additional file 2. The binding signals of some selected TRFs in K562 are shown in Figure 5.

As expected, the binding signal of RNA polymerase II (POL2RA) is strongly enriched at PRMs compared to the genomic background, and at DRMs to a lesser extent. In contrast, the binding signal of RNA polymerase III (POL3RA), which transcribes some non-coding RNAs, such as rRNAs and tRNAs, is not enriched at PRMs and only slightly enriched at DRMs.

DRMs have stronger binding signals of CTCF and the cohesin proteins RAD21 and SMC3 than PRMs, which in turn have stronger binding signals than the whole genome in general. The stronger signals at DRMs than PRMs is consistent with the known role of CTCF in binding insulators [51,52] and the frequent co-occurrence of the binding sites of CTCF and the cohesin complex [53,54]. On the other hand, the stronger signals at PRMs than the genomic background suggest that CTCF also binds some proximal regions, which may reflect the ability of it to act as a transcriptional insulator, repressor or activator depending on the context of the binding site [55,56]. A recent study also found that, contrary to the enhancer blocking model, CTCF may actually promote communication between functional regulatory elements by connecting promoters and enhancers through long-range DNA interactions [57].

EP300, which is found at some enhancers [58], has a slight enrichment at DRMs. The same trend is also observed for GATA1 and GATA2 (Figure 5d; Figure S8 in Additional file 2), which were reported to enhance the expression of some genes [59,60]. In comparison, some TRFs (such as E2F4) are strongly enriched at PRMs compared to DRMs, and some (such as USF2) have almost the same enrichment at PRMs and DRMs.

As defined, HOT regions have strong binding signals of many TRFs, a lot of which do not usually bind the same sites. LOT regions, on the other hand, have only weak binding signals.

In addition to binding signals measured from ChIP-seq experiments, we also studied binding peaks of the TRFs called by the ENCODE procedure, which can be considered as the locations with the strongest binding signals compared to the local genomic background. For each TRF binding experiment, we computed the fraction of peaks within each of the six types of regions and the intergenic portions of HOT and LOT regions (Figure S9 in Additional file 2). In most cases, most binding peaks are within BARs. Specifically, considering all five cell lines, in about half of the experiments more than 90% of the binding peaks are within BARs. The distribution of binding peaks between PRMs and DRMs generally agrees with our observations in the analysis of binding signals. In K562, for example, E2F4 has 52% binding peaks at PRMs and only 11% at DRMs, while GATA2 has the reverse trend, with 14% binding peaks at PRMs and 26% at DRMs.

Some TRFs preferentially bind intergenic HOT regions. In K562, for example, 17% of EP300 binding sites are at intergenic HOT regions, which is likely due to enhancers in these regions. On the other hand, the RNA polymerase III protein POLR3G and the TFIIIB transcription initiation complex subunits BDP1, BRF1 and BRF2 have, respectively, 29%, 24%, 30% and 24% of their binding sites at intergenic HOT regions, which may mark promoters of yet unannotated non-coding genes.

### Identification and validation of potential enhancers

To explore potential functional roles of our identified DRMs, we derived computational methods for predicting distal enhancers and tested these predictions using reporter assays.

#### First round of validation: human enhancers active in mouse embryos

We first predicted potential human enhancers that are active in mouse embryos on embryonic day 11.5. Specifically, from the list of BARs, we selected those that are far away from TSSs and exons, and scored them based on both their sequence conservation and the presence of motifs of TRFs known to be expressed in mouse embryos (Materials and methods). We then took the top 50 predictions, and randomly chose 6 of them for experimental validation (Table S3 in Additional file 1). These six regions were extended according to some experimental requirements, and tested for enhancer activities in a mouse assay previously established [61]. These experiments were performed by Dr Len Pennacchio's group, for testing a larger cohort of, in total, 33 potential enhancers identified by several sub-groups of the ENCODE consortium using different prediction methods (Pennacchio and The ENCODE Project Consortium, unpublished data).

Among our 6 tested predictions, 5 (83%) were found to have enhancer activities in various tissues with good reproducibility (Table 2; data available at the VISTA database [6]). Interestingly, most predicted enhancers were found to be active in tissues related to neurodevelopment, which is likely due to the particular set of development-related TRFs we considered in our method.

**Table 2:** Results of the predicted enhancers for experimental validation in the first round of mouse reporter assays

Coordinates (hg19)			VISTA ID	Enhancer activity	Tissues with enhancer activity	Reproducibility
Chr2	145339602	145341530	hs1802	Positive	Midbrain (mesencephalon)	8/8
Chr7	115451531	115454796	hs1798	Positive	Eye	6/9
					Forebrain	9/9
					Hindbrain (rhombencephalon)	6/9
					Midbrain (mesencephalon)	6/9
					Neural tube	6/9
Chr7	121967528	121971078	hs1809	Positive	Forebrain	9/9
					Hindbrain (rhombencephalon)	7/9
					Midbrain (mesencephalon)	7/9
					Neural tube	5/9
Chr8	106602865	106607408	hs1800	Positive	Cranial nerve	8/10
					Dorsal root ganglion	6/10
					Midbrain (mesencephalon)	9/10
					Trigeminal V (ganglion, cranial)	8/10
Chr11	118308306	118311240	hs1793	Negative		
Chr14	57474144	57478090	hs1791	Positive	Midbrain (mesencephalon)	15/15

#### Second round of validation: General human enhancers in the whole genome

With the initial success in the first round of small-scale experimental validations, we set out to take on the more difficult task of predicting all enhancers in the human genome. It was part of a larger effort of ENCODE to predict and experimentally validate various types of DNA elements, including promoters, enhancers and insulators. The predictions were made by different methods and validated by *in vivo *assays in transgenic mouse embryos and Medaka fish [20].

In order to identify general enhancers, we modified our prediction procedure to replace information specific to the mouse assay, such as the binding motifs of TRFs expressed in mouse embryos, by some general features of enhancers, such as signals of the histone modification H3K4me1. We developed two complementary methods, and took the intersection of them as our high-confidence predictions (Materials and methods). In total, we identified 13,539 potential enhancers (full list available in Additional file 1), among which 50 were randomly chosen; 20 of them were tested by the mouse assay, and an independent set of 27 were tested by the Medaka fish assay (Materials and methods).

The validation results for the mouse and fish assays are shown in Tables 3 and 4, respectively. In the mouse experiments, 6 of the 20 (30%) tested sequences showed enhancer activities in various types of tissues in the nose, heart, limb and tail. In the fish experiments, 19 of the 27 (70%) tested sequences showed some enhancer activities, out of which 15 (56%) had strong activities.

**Table 3:** Results of the predicted enhancers for experimental validation in the second round of mouse reporter assays

Coordinates (hg19)			VISTA ID	Corresponding ID in mouse assay	Enhancer activity	Tissues with enhancer activity	Reproducibility
Chr1	39,629,409	39,631,707	hs1999	ENH_DISCR_2	Negative		
Chr2	7,025,512	7,027,025	hs2015	ENH_DISCR_18	Negative		
Chr2	68,419,973	68,421,991	hs2040		Positive	Nose	3/5
Chr2	159,885,988	159,889,012	hs2027		Positive	Heart	11/11
Chr2	169,971,561	169,974,373	hs2034	ENH_DISCR_37	Negative		
Chr3	65,589,981	65,591,565	hs2038	ENH_DISCR_41	Negative		
Chr3	72,368,145	72,370,230	hs2006		Negative		
Chr3	124,304,700	124,307,917	hs2031	ENH_DISCR_34	Negative		
Chr3	141,579,463	141,580,810	hs2041	ENH_DISCR_44	Positive	Limb	3/7
Chr4	109,893,210	109,895,294	hs2021	ENH_DISCR_24	Negative		
Chr5	176,076,732	176,078,530	hs2007		Positive	Heart	3/7
Chr8	59,769,794	59,772,587	hs2029	ENH_DISCR_32	Negative		
Chr9	75,758,359	75,760,288	hs2000		Negative		
Chr11	74,781,340	74,785,285	hs2047		Negative		
Chr12	31,867,650	31,868,817	hs2043		Negative		
Chr16	89,384,737	89,387,643	hs2036		Negative		
Chr17	45,368,528	45,369,514	hs2033		Positive		
Chr17	73,347,819	73,348,933	hs2023	ENH_DISCR_26	Negative		
Chr20	48,291,612	48,294,178	hs2045	ENH_DISCR_48	Negative		
Chr22	21,953,368	21,954,302	hs2026	ENH_DISCR_29	Positive	Tail	9/17

**Table 4:** Results of the predicted enhancers for experimental validation in the Medaka fish reporter assays

Coordinates (hg19)				ID	Enhancer activity	Tissues with patterns
Chr1	39630305	39631117	+	ENH_DISCR_2	Positive	Tectum, fin
Chr1	27448948	27449785	-	ENH_DISCR_38	Negative	
Chr2	64877588	64878573	-	ENH_DISCR_16	Negative	Not consistent
Chr2	7025939	7026794	+	ENH_DISCR_18	Positive	Telencephalon
Chr2	169972473	169973432	-	ENH_DISCR_37	Positive	Epidermis
Chr3	20009087	20009933	+	ENH_DISCR_14	Negative	Not consistent
Chr3	71276246	71277150	-	ENH_DISCR_19	Negative	Not consistent
Chr3	124305687	124306362	+	ENH_DISCR_34	Positive	Epidermis
Chr3	65590259	65591167	-	ENH_DISCR_41	Positive	Blood_heart
Chr3	141579681	141580471	+	ENH_DISCR_44	Weak	Blood
Chr4	109893826	109894623	-	ENH_DISCR_24	Negative	Not consistent
Chr6	158653651	158654413	+	ENH_DISCR_1	Negative	Not consistent/heart
Chr8	91239118	91239934	-	ENH_DISCR_17	Positive	Telencephalon
Chr8	59770666	59771377	-	ENH_DISCR_32	Positive	Telencephalon
Chr10	97054745	97055495	-	ENH_DISCR_47	Positive	Epidermis
Chr12	95567438	95568125	-	ENH_DISCR_35	Positive	Blood, ear
Chr12	755392	756170	-	ENH_DISCR_45	Weak	Epidermis
Chr14	35805596	35806453	+	ENH_DISCR_21	Positive	Epidermis, late
Chr15	89638466	89639233	+	ENH_DISCR_12	Negative	
Chr17	46503536	46504314	-	ENH_DISCR_13	Positive	Telencephalon
Chr17	34953545	34954303	+	ENH_DISCR_22	Weak	Epidermis, blood
Chr17	73347806	73348761	-	ENH_DISCR_26	Negative	Not consistent
Chr17	76254538	76255291	+	ENH_DISCR_31	Weak	Blood
Chr19	33162656	33163445	-	ENH_DISCR_40	Positive	Blood
Chr20	48293080	48293844	-	ENH_DISCR_48	Positive	Epidermis, blood
Chr22	28430853	28431678	-	ENH_DISCR_25	Positive	Tectum
Chr22	21953421	21954149	+	ENH_DISCR_29	Positive	Telencephalon

Eleven predictions were tested in both types of assays (Table 3). In seven cases, enhancer activities were detected only in the fish experiments, which highlights the condition specificity of enhancers and the benefits of combining results of multiple types of experiments.

Our predictions achieved a higher success rate in the fish assay than a random background set (1/10 = 10% with weak activities), a set of baseline predictions picked from repeat-free regions with binding motifs in Transfac [62] (14/26 = 54% with some activities, out of which 8/26 = 31% had strong activities), and a computational method that segments the whole genome into different classes based on chromatin features (17/29 = 59% with some activities, out of which 15/29 = 52% had strong activities) [20].

Comparing the results of the two rounds of experimental validations, while it is hard to draw a definitive conclusion due to the small number of predictions tested, the success rate of our predictions in the first round appears to be higher. This is expected as the problem settings for the two rounds are very different. In the first round, we made only a small number of predictions, which correspond to the most confident cases with the strongest signals. In contrast, in the second round, we made a much larger number of predictions in order to identify all potential enhancers in the human genome. The lower precision is at least partially compensated for by a higher recall rate. Furthermore, in the first round of predictions we optimized our method for a particular assay, while in the second round we adopted a more general procedure. Some of our predicted enhancers in the second round may only be bound by TRFs that are not expressed at the particular stages of the tested animals. Indeed, the diversity of tissues in which some of our predictions were shown to be positive suggests that they were targeted by a heterogeneous set of TRFs.

In summary, in the two rounds of validation experiments, 42 unique regions were tested and 28 of them (67%) showed enhancer activities in at least one assay.

### Identification of potential long-range TRF regulation through DRMs

As a next step to identifying distal regulatory elements with functional roles, we studied potential target genes of the identified DRMs, and the TRFs that regulate these genes through the DRMs. A method for associating potential target genes and predicted enhancers identified by a genome segmentation approach has been recently proposed [63]. The main idea was to look for pairs of predicted enhancers and genes where the signals of some histone modifications characteristic for enhancers (such as H3K4me1 and H3K27ac) at the enhancer could predict the expression level of the gene in the same pair across multiple cell lines. We used a similar approach to associate our DRMs with potential target transcripts (Materials and methods; Figure S10 in Additional file 2). However, instead of manually picking histone modifications known to be related to a particular type of DRMs, we correlated all types of histone modifications in our dataset with expression of transcripts in an exhaustive manner, so that previously unknown functions of histone modifications at DRM sites may also be discovered. To minimize false positives, we used a stringent correlation threshold after correcting for multiple hypothesis testing. Subsequently, for each identified DRM-target transcript pair, we associated TRFs that may be involved in the long-range regulation by looking for TRFs with a binding peak at the DRM in a cell line where there was a strong signal of the histone modification used in correlating the pair. We also used these TRF-potential target gene pairs to form a distal regulatory network and performed some additional analyses in a separate study [31].

For this set of analyses, we also used other ENCODE cell lines with both histone modification and expression data in our dataset in addition to the five focused on in this paper in order to increase statistical power (Materials and methods).

**Figure 6:**
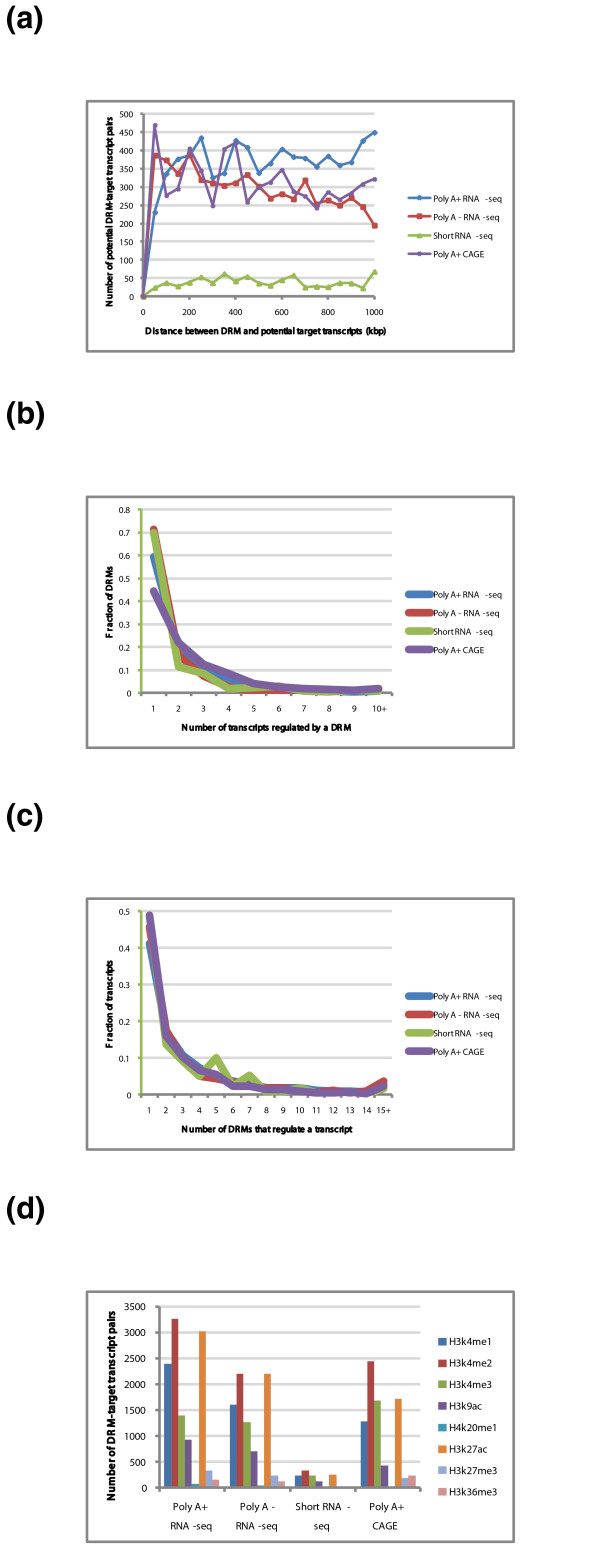
**Associating DRMs with potential target transcripts and TRFs involved**. **(a) **Distance distribution between DRMs and potential target transcripts for four different types of gene expression experiments. **(b) **Distributions of the number of transcripts that each DRM potentially regulates; 10+ denotes 10 or more transcripts. **(c) **Distributions of the number of DRMs that each transcript is potentially regulated by; 15+ denotes 15 or more DRMs. **(d) **Distributions of the number of DRM-target transcript pairs with which each type of histone modification is involved.

From the different types of histone modification and gene expression experiments, we identified between 8 and 3,270 pairs of potential DRM-target transcripts. The distance distributions between DRMs and target transcripts show some interesting patterns (Figure 6a). For expression values measured by Poly A+ (Poly A enriched) RNA-seq or Poly A+ CAGE, many of which are expected to be mRNAs of protein-coding genes, DRMs as far away as 1 Mbp from the potential target transcript are as common as those only about 100 kbp apart. In contrast, for transcripts measured by Poly A- (Poly A depleted) RNA-seq, more of which are expected to be non-coding RNAs, the frequency of DRM-target transcript pairs decreases as the distance between them increases. For small RNAs, the number of DRM-target transcript pairs is much lower than for long RNAs, but this is mainly due to a smaller number of available datasets for small RNAs so that fewer transcripts survive the filtering conditions (Materials and methods).

While some of the identified pairs may be false positives, there is no apparent systematic bias in our procedure that may cause the observed difference between the Poly A+ and Poly A- cases. We propose that the difference could be related to the number of transcripts each DRM regulates. We observed that, in general, each DRM regulates a larger number of Poly A+ transcripts than Poly A- transcripts (Figure 6b). For example, on average, each DRM regulates 2.5 transcripts according to Poly A+ CAGE, but only 1.8 and 1.5 transcripts according to short RNA-seq and Poly A- RNA-seq, respectively. Some of these cases are caused by single DRMs regulating multiple transcripts of the same gene, due to protein-coding genes with many isoforms. In some other cases, the difference is due to the regulation of more genes by one DRM. As the distance between different genes is, on average, larger than the distance between different transcripts of the same gene, it is the latter case that helps explain the longer distance between DRMs and their potential target genes for Poly A+ transcripts.

This explanation is consistent with a recent finding that DNA sometimes forms loops through long-range interactions, to bring multiple anchor genes into close physical proximity [64]. Such anchor genes were found to be more active than genes in loops that involve only two DNA regions in terms of binding signals of RNA polymerase II.

We also checked the number of DRMs by which each transcript is regulated. The trends are similar for the different types of expression experiments (Figure 6c). About 40 to 50% of transcripts are regulated by only one DRM, but there is also a significant portion of transcripts regulated by two or more DRMs. As we have used a very conservative procedure for calling DRM-target transcript pairs, we believe this is an underestimate of the actual number of regulating DRMs per transcript.

Our procedure for associating DRMs and target transcripts could, in principle, detect both statistically significant positive and negative correlations between the histone modification signals at the DRM and the expression level of the target transcript. In reality the vast majority (almost 100%) of our identified pairs have a positive correlation. When we examined the actual types of histone modifications, we found that enhancer-related marks, including H3K4me1, H3k4me2 and H3K27ac, are involved in a large fraction of the significant correlations (Figure 6d). The active promoter mark H3K4me3 is also involved in a large fraction of cases, which may indicate unannotated transcripts (for example, non-coding transcripts) or a role of the mark at some DRMs. We also observed the involvement of the active chromatin mark H3K9ac in a smaller yet significant fraction of the pairs. Indeed, while H3K9ac is most enriched at PRMs, it also has a clear enrichment at DRMs compared to the genomic background (Figure S8 in Additional file 2). Finally, the presence of the repressive mark H3K27me3 and active mark H3K36me3, usually found at gene bodies in a small fraction of our cases, may be used to estimate the amount of false positives on our list, although we cannot eliminate the possibility of their potential roles in gene regulation at DRMs.

We then examined the TRFs associated with the DRM-target transcript pairs. We found that DRMs potentially regulating Poly A+ transcripts have a higher fraction of EP300 binding than both the set of all DRMs and the whole genome (except in H1-hESC, which has too few DRMs to compute the fraction accurately; Table S4 in Additional file 1). This observation suggests that the correlation method for associating DRMs and target transcripts could help identify DRMs that have stronger activities.

We also studied if there are CTCF binding sites between our DRMs and potential target transcripts. Traditionally, CTCF is assumed to play a role in blocking enhancers [65]. We found that in 97% of our DRM-target transcript pairs, there is at least one CTCF binding peak between them, which suggests that CTCF is not generally blocking long-range interactions for our set of identified cases. We hypothesize that CTCF blocking may have a stronger effect for enhancers just a few kilo-base pairs from TSSs due to space constraints, but for our DRMs, which are more distal from TSSs, there is a higher flexibility of the DNA three-dimensional structure between the DRM and the target transcript so that CTCF may play a smaller blocking role. In addition, a recent study of CTCF-mediated chromatin interactions has suggested that CTCF may actually facilitate the cross-talk between promoters and regulatory elements [57], which may also explain some of our cases.

### Motifless binding at HOT regions

In a separate analysis we have found that some ChIP-seq binding peaks do not have strong DNA sequence motifs of the corresponding TRFs [20]. They also have lower binding affinity in general. In the current study we explored a potential relationship between these regions and our identified HOT regions.

For each TRF, we examined its binding peaks and identified those that do not contain any previously known or newly discovered DNA binding motifs of it (Materials and methods). We called them motifless binding peaks of the TRF. We then collected the motifless binding peaks of all TRFs for each cell line, and compared them with our HOT regions.

Using the whole set of binding peaks of all TRFs in each cell line as background, we found that motifless binding peaks have very significant overlaps with our HOT regions (Table 5). This is true no matter whether we consider all TRF peaks in the whole genome, or only those in intergenic regions. In all cases, the z-score is more than 25, which corresponds to a *P*-value <3 × 10^-138^. A substantial portion of binding at HOT regions is thus attributed to non-sequence-specific binding. In our separate study, we found that motifless binding peaks have stronger DNase I hypersensitivity signals [20], which is also a signature of our HOT regions (Figure 4).

**Table 5:** Comparisons of motifless binding peaks and our HOT regions

Cell line		1. TF binding peaks	2. HOT regions	3. HOT regions within TF binding peaks	4. Motifless binding peaks	5. Intersection of #3 and #4	6. Z-score of intersection
GM12878	WG	123,982 (125 Mbp)	92,592 (25.9 Mbp)	77,583 (22.9 Mbp)	69,568 (17.4 Mbp)	43,581 (8.68 Mbp)	86.5
	IR	37,746 (29.5 Mbp)	21,465 (5.82 Mbp)	17,647 (5.02 Mbp)	18,973 (4.67 Mbp)	10,568 (2.07 Mbp)	49.8
H1-hESC	WG	98,963 (81.8 Mbp)	105,036 (26.4 Mbp)	74,727 (20.4 Mbp)	38,631 (8.91 Mbp)	25,042 (4.59 Mbp)	42.0
	IR	32,540 (20.8 Mbp)	27,212 (6.43 Mbp)	18,399 (4.73 Mbp)	11,242 (2.55 Mbp)	6,267 (1.13 Mbp)	25.3
HeLa-S3	WG	112,657 (100 Mbp)	95,054 (25.6 Mbp)	57,045 (17.0 Mbp)	47,556 (13.4 Mbp)	26,112 (5.18 Mbp)	46.5
	IR	36,252 (26.1 Mbp)	25,733 (6.70 Mbp)	14,822 (4.30 Mbp)	13,872 (3.81 Mbp)	7,023 (1.40 Mbp)	38.2
Hep-G2	WG	193,990 (131 Mbp)	95,998 (26.3 Mbp)	64,147 (19.1 Mbp)	63,523 (16.1 Mbp)	31,179 (6.32 Mbp)	75.4
	IR	67,013 (36.9 Mbp)	25,402 (6.74 Mbp)	15,565 (4.50 Mbp)	18,162 (4.54 Mbp)	7,492 (1.52 Mbp)	58.2
K562	WG	159,029 (140 Mbp)	81,436 (26.6 Mbp)	59,716 (19.8 Mbp)	71,912 (19.8 Mbp)	37,108 (8.03 Mbp)	63.4
	IR	48,966 (34.9 Mbp)	17,994 (5.18 Mbp)	12,884 (3.84 Mbp)	18,194 (4.80 Mbp)	8,057 (1.67 Mbp)	53.4

In each cell, the number of regions is given, followed by the total length of DNA covered in parentheses. IR, intergenic regions; WG, whole genome.

Our analysis also highlights the need for a more comprehensive catalog of sequence motifs of DNA binding proteins. If we instead define a TRF binding peak as motifless as long as it lacks either a previously known motif or a newly discovered one - that is, it could still have a motif from the other source - the overlap of the resulting 'motifless' peaks with our HOT regions becomes statistically insignificant. Requiring a motifless binding peak to lack both types of motifs is likely more reliable.

## Discussion

### Methods for identifying regulatory modules *in silico*

There have been a lot of efforts in the past few years to identify transcriptional regulatory modules computationally [8,9]. The majority of the methods rely on evolutionary conservation and sequence-based features such as degenerate binding motifs of TRFs. It is now well-accepted that protein-DNA binding depends not only on these static features, but also on other dynamic factors such as chromatin states. Recently, cell-specific chromatin features have been used to segment the human genome into different types of regions [63], which marks an important step forward towards the identification of cell-specific regulatory modules. In the current study a lot of protein binding data are used as examples to learn statistical models for TRF binding sites, taking even more chromatin features into account. We hope the six types of regions defined in this study will serve as a good reference for future studies of regulatory modules and for further improving computational methods for identifying them.

### Supervised and semi-supervised prediction of enhancers

Our procedure for identifying enhancers involved the use of 'supervised' machine learning methods - methods that learn model parameters from known examples. However, our overall pipeline is not truly supervised in that we used only supervised models to learn regions needed by the procedure to identify enhancers, such as BARs and PRMs. These regions were then used in an unsupervised manner in the final prediction of enhancers. This design was driven by an insufficient number of cell-type-specific positive and negative examples of enhancers. While there are large enhancer catalogs, such as the VISTA database [6], most of the validation experiments were done in specific assays (such as embryos of transgenic mouse) that may not be appropriate as examples for other cell types due to the dynamic nature of protein binding and gene regulation. In fact, when we tried to use data from VISTA to learn direct supervised models for enhancers using chromatin data from our cell lines as features, the prediction accuracy was low according to some left-out data not used in model training. We hope that with the larger-scale validation efforts of ENCODE [20] and other groups, more cell-type-specific data will become available and the construction of highly reliable, supervised predictive models of enhancers will become possible.

It is also useful to consider semi-supervised methods [66], which consider data patterns of both regions of known types and other regions. For instance, one approach worth investigating is combining the information captured by our method and some segmentation methods [63,67]. As a first step towards this direction, we have taken the intersection of the predicted enhancers produced by the two approaches, and provide the files in Additional files 1 and 2.

### Accurate association of DRMs and target genes

Our procedure for associating DRMs and potential target genes is currently constrained by a small number of cell types for which both histone modification and gene expression data are available. Simply by chance it is possible to have a DRM that appears highly correlated with a gene. It is also difficult to distinguish between direct regulation and indirect correlations due to co-expressed genes. As a result, we decided to use a very stringent procedure based on the Bonferroni correction method for multiple-hypothesis testing, which is known to be too conservative. While the procedure gives us some associations that are of higher confidence than ones possibly called by a less stringent procedure, one obvious drawback is an expected high false negative rate. Our analysis may also be biased, since the DRM-target transcript pairs that survive the stringent criteria are likely the most extreme cases. We believe one direct consequence is the lack of negatively correlated pairs on our identified list. It appears that positive regulation events at enhancers result in more extreme positive correlations than the negative correlations caused by negative regulation events at DRMs such as silencers. We expect that a more complete picture of gene regulation through DRMs will be drawn when data from more cell types become available.

Another promising direction for associating DRMs with target genes is by using whole-genome DNA long-range interaction data, either involving a target protein that mediates the interaction (such as ChIA-PET [28]) or without (such as Hi-C [68]). Currently, there are few datasets available, and among these, some suffer low reproducibility [64] and low resolution [68]. Some technological advancements that lead to better data quality are already underway [69]. We hope that the study of long-range gene regulation will be facilitated by large-scale, high-quality DNA interaction data in the coming years.

### Some possible interpretations of HOT regions and improvements of the calling procedure

We have found that there are regions bound by many different TRFs in the same cell line, which we call HOT regions. As discussed, the observed binding of many TRFs at a small region may be due to the average of a cell population. We found that these regions have high DNase I hypersensitivity in general, as well as high signals of almost all types of histone modification (Figure 4). The strong signals suggest that they could be regions with general open and accessible chromatin, where TRFs can easily bind them even without cognate sequence motifs.

It has also been shown that the binding of a TRF may promote steady-state binding of other TRFs, even for those that share the same DNA response elements [70]. This observation was explained by an 'assisted loading' mechanism, where the binding of a TRF increases local chromatin accessibility, and makes it easier for other TRFs to bind regions nearby. HOT regions could be extreme examples of such assisted loading.

To further study HOT regions, it is of utmost importance to make sure that the co-occurrence of binding of different TRFs is not due to experimental or computational artifacts, such as erroneous read mapping (for example, by mapping all reads of a broad repeat region to the same copy of the repeats, which would result in an artificially strong binding signal of the region), or natural co-binding of TRF co-factors. We have applied a rigorous procedure to eliminate as many of the issues in data quality, reproducibility, mapping, and global co-binding as possible. We have also partially taken into account the non-uniform nature of TRF binding in the whole genome, by using a co-occurrence matrix of TRF binding peaks produced by a method based on Genome Structure Correction [20,71]. We propose that the procedure for calling HOT regions can be further improved by directly applying Genome Structure Correction in evaluating the statistical significance of binding profiles, and considering the local context of different regions. For example, it may be more biologically interesting to see the binding of many TRFs at an unannotated intergenic region than at the promoter of a highly expressed gene. To give a higher HOT score to the former, the HOT region identification method needs to evaluate the statistical significance based on a background distribution specific to the type of regions of interest. It can be roughly done by calling HOT regions of different classes of annotated elements (for example, promoters versus gene bodies versus intergenic regions) separately. To deal with the large fraction of intergenic regions in the genome, the functions of which are still not well understood, the unsupervised segmentation approach [63,67] provides one systematic way to define the different element classes at the genome scale.

### Identified regions as a resource

We make available our three paired types of regions from the five cell lines as supplementary files [26], in standard formats that can be easily loaded into genome browsers as data tracks. We also provide some additional files, such as predicted DRM-target transcript pairs and the TRFs involved. Details of all these files can be found in Additional files 1 and 2.

## Materials and methods

### Source of ENCODE data

The raw sequencing data for TRF binding (Table S1 in Additional file 1), histone modification (Table S2 in Additional file 1), open chromatin signals and expression values used in this study can be downloaded from the UCSC Genome Browser [72]. The complete list of datasets, their unique identifiers and download paths can be found in Table S5 in Additional file 1.

### Identifying BARs and BIRs

The human reference genome (build hg19) was divided into 100 bp bins. For each cell line, we collected chromatin features from ENCODE and computed the average signal of each feature across the 100 bp of each bin. The features include DNase I hypersensitivity, FAIRE, and histone modifications [20]. Bins that overlap with the binding peak of a TRF were collected as positive examples of TRF binding sites. To avoid long running time of computer programs, 5,000 of these positive bins were randomly sampled; 5,000 non-positive bins were randomly sampled from the whole genome as negative examples. These two sets of examples were used to train random forest classifiers using Weka [73] as follows. The examples were divided into ten disjoint subsets with equal size. A ten-fold cross-validation procedure was applied, with nine subsets used to train a classifier and the remaining subset used to test its performance, where each of the ten subsets acted as the testing set in turn. Each time a BAR score was given for each bin, and the order of these scores was used to construct the receiver-operator-characteristic (ROC) and precision-recall (PR) curves. The final accuracy values were computed as the average areas under the curves of the ten test sets. Since the negative examples may contain binding peaks of TRFs not included in the dataset and binding sites of the included TRFs that are not strong enough to be called as peaks, the reported accuracy values are only rough estimates of the ability of the learned models to identify binding active regions. The final list of BARs was composed of bins with an average BAR score from the ten folds larger than 0.9. Bins with an average BAR score <0.1 and not overlapping binding peaks of any TRFs in the dataset were collected to form the list of BIRs.

### Identifying PRMs and DRMs

A machine-learning procedure similar to the one for identifying BARs was applied to identify PRMs. The same datasets were used as features of 100 bp bins. In this case, the positive set was composed of bins at the TSSs of expressed genes, defined as genes with at least one read per kilobase per million mapped reads (RPKM) [29] in an RNA-seq experiment or at least 1 read per million mapped reads (RPM) in a CAGE or diTag experiment conducted for the cell line. The negative examples were composed of random bins from three different sets: 1) bins not overlapping with TRF binding bins in the whole genome; 2) non-POL2RA TRF binding peaks at least 10,000 bp away from any coding and non-coding gene annotated in Gencode version 7 level 1 and level 2; and 3) bins not overlapping with TRF binding peaks between 1,000 and 5,000 bp upstream or between 200 and 1,000 bp downstream of a TSS. The three subsets ensure that the negative set contains bins that are non-TRF binding, TRF binding but not close to annotated genes, and promoter-proximal but with a lower chance of TRF biding. The third subset was specifically included so that the resulting models do not simply use open chromatin as the single most important feature to identify PRMs. For each cell line, a model was trained to give a PRM score for each bin. The average PRM score with exactly 1% negative examples higher than it was used as the threshold. The final list of PRMs consists of bins with an average PRM score higher than the threshold. The DRM bins were then defined as non-PRM BAR bins at least 10 kbp from any Gencode version 7 level 1 and level 2 coding and non-coding genes.

### Identifying HOT and LOT regions

For each cell line, we grouped different experiments for the same TRF together and computed the average binding signal for each 100 bp bin. The values were then discretized into five values: top, second, and third 25 percentiles, fourth 25th percentile that are not zeros, and zeros. The extra group for zeros was to handle the large number of zeros in a typical ChIP-seq experiment for TRF binding. For each bin, we then computed a degree of region-specific co-occurrence, which is a weighted sum of the discretized values of the bin from the different TRFs. The weight of each TRF was computed as follows. First, we took the global co-occurrence z-score matrix of TRF binding peaks computed by using Genome Structure Correction [20,71]. A raw score of each TRF was computed as the average z-score with all other TRFs in the matrix. The raw score was then normalized linearly so that the TRF with the lowest score received a weight of 1 and the TRF with the highest score received a weight of 1/n, where n is the total number of TRFs with ChIP-seq data from the cell line. This weighting scheme de-emphasizes TRFs that are globally co-associating with other TRFs in the counting of region-specific co-occurrence of binding. The HOT and LOT regions were then defined as the bins with the top 1% degrees of region-specific co-occurrence and the bins with the bottom 1% non-zero degrees of region-specific co-occurrence, respectively.

### Constructing box-and-whisker plots for open chromatin, histone modification and TRF binding signals

For each 100 bp bin within a type of regions and each open chromatin, histone modification or TRF binding dataset, we computed the average signal value of the dataset within the 100 bp bin. We represent the resulting distributions by box-and-whisker plots. To prevent extreme outliers from dominating the scales of the plots, we excluded outliers smaller than Q1 - 5 IQR and those larger than Q3 + 5 IQR, where Q1 is the bottom 25th percentile, Q3 is the top 25th percentile, and IQR is the inter-quartile range, defined as Q3 - Q1.

### First round identification and validation of potential enhancers in mouse embryos

We combined the ENCODE chromatin data available for GM12878 and K562 as of January 2010 to predict binding active regions using a pipeline similar to the one for the BARs in Figure 1. We removed bins within 2 kbp upstream or 500 bp downstream of Gencode TSSs, and bins within 1 kbp from Gencode and Refseq exons. We then downloaded the phyloP conservation scores [74] of the resulting bins from the UCSC Genome Browser [72] based on multiple sequence alignments of 44 vertebrate genomes, and took the top 2% of the bins with the highest scores, corresponding to a cutoff score of 1.2. We merged adjacent bins into longer regions, and kept only those merged regions with a size between 0.8 and 4 kbp. After that, for each merged region we counted the number of binding motifs of a set of TRFs known to be highly expressed in mouse embryos based on a gene expression atlas [75]. The genes include members of the OCT and SOX families among others. The motifs of these genes were taken from Transfac [62]. The top 50 predictions with the highest binding motif density were then used as candidates of potential enhancers.

The predictions were originally made according to human reference genome build hg18. We used the LiftOver tool [76] at the UCSC genome browser to convert the coordinates into human reference genome build hg19.

The enhancers were tested in embryos of transgenic mice on day E11.5 with a lacZ reporter gene fused with an hsp68 promoter as previously described [61].

### Second round, whole-genome identification and validation of potential enhancers in mouse and Medaka fish embryos

We developed two methods to identify potential enhancers in the whole human genome, and took the intersection of their predictions to form our candidate set for experimental validation. We used data from K562, as the initial plan was to test the enhancers *in vitro *in K562 cells.

The first method is a variation of the method for the first round of enhancer prediction. We took the BARs and removed from them all bins either with a promoter score >0.8, within 2 kbp from a Gencode version 3c TSS, intersecting with a Gencode exon, or with a phastCons primate score <0.1 downloaded from the UCSC Genome Browser. We then merged adjacent bins in the resulting set into longer regions, and removed regions with no binding motifs of TRFs expressed in K562. The final list contains 55,857 regions.

The second method used a two-stage method to learn locations of TRF binding sites from chromatin, conservation, sequence and gene annotation features. In the first stage, large windows of 1 kbp were made and feature values were aggregated to learn statistical models for distinguishing TRF binding peaks from random locations. In the second stage, the shapes of TRF binding signals around binding peaks were used to construct features for learning models that distinguish binding peaks from flanking regions. From the resulting list of regions predicted to have active TRF binding, repeats were removed and the high-scoring ones were kept. The list was then further filtered by removing regions that overlap Gencode version 3c exons or within 2 kbp from a Gencode TSS. Finally, we considered only candidate regions that involve H3K4me1 or H3k4me3 in their prediction process. The resulting list contains 56,256 regions.

We then combined the two lists by taking their intersection, and refined the boundaries of each region so that each has a minimum length of 100 bp and a maximum length of 700 bp. We further considered the high-confidence ones with median H3K4me1 or H3K4me3 signals >5. The final list contains 13,539 sequences of potential enhancers.

The mouse assay was performed in same way as in the first round of validation. The Medaka fish assay was performed over the first three days of development, as described [20].

### Associating DRMs with target transcripts and the TRFs involved

We took the union of the DRM bins identified from the five cell lines to form a comprehensive set of non cell-line-specific potential DRM bins. We merged adjacent bins into modules, allowing 100 bp gaps between any two DRM bins, resulting in 129,326 modules (Figure S10A in Additional file 2). We then took all Gencode version 7 level 1 and level 2 transcripts, and filtered out those with <2 RPM/RPKM in all cell lines with expression data of the transcript or less than two-fold expression level difference among the cell lines. The resulting set contains 64,075 transcripts.

We considered four types of gene expression experiments in whole cells: Poly A+ RNA-seq, Poly A- RNA-seq, RNA-seq of short RNAs, and Poly A+ CAGE (Figure S10B in Additional file 2). For each DRM, we considered only histone modifications with at least a signal value of 2 in one or more cell lines, and at least a two-fold signal difference among the cell lines. For the DRMs and transcripts that pass the above selection criteria, we considered only pairs with at least seven matching cell lines for both histone modification and gene expression data, and are on the same chromosome no more than 1 Mbp apart, where this distance threshold was based on a recent finding that there are few long-range DNA interactions that span more than 1 Mbp for a TRF according to some ChIA-PET experiments [64]. Finally, we computed Pearson correlations for these pairs, and kept the ones with a Bonferroni-corrected *P*-value <0.01 based on Fisher's transformation. Depending on the type of histone modifications and RNA experiments, 8 to 3,270 DRM-target transcript pairs were identified (Figure S10B in Additional file 2).

We next associated TRFs with each DRM-target transcript pair by considering TRFs with a binding peak at the DRM in a cell line with a signal value of 2 or more for the histone modification involved, which resulted in 4 to 2,129 potential TRF-target transcript pairs connected by the DRMs.

### Defining motifless binding peaks and comparing them with HOT regions

For each cell line and each TRF with ChIP-seq experiments in the cell line, we collected the binding peaks of the TRF, and identified the ones that do not contain a binding sequence motif of it. This requires that the binding peak contains neither a previously known motif nor a motif newly discovered from ENCODE data. These two lists of motifs and their occurrences in the human genome were produced by a separate pipeline [77]. For each cell line, we then collected all these regions to form the set of motifless binding peaks for the cell line. In this procedure, a region is defined as a motifless binding peak as long as one TRF has a binding peak there without a corresponding sequence motif, but the region is allowed to contain sequence motifs of other TRFs.

We then intersected the motifless binding peaks with our HOT regions. Since our HOT regions were identified from the whole human genome but the motifless binding peaks were all from ChIP-seq binding peaks, we first identified the subset of HOT regions within these peaks. We then determined their intersection with the motifless binding peaks, and evaluated the statistical significance of the intersection by block sampling [71], using the whole set of binding peaks as the domain. For each cell line, we took 100,000 random block samples and computed the intersection in each of them in terms of base overlap ratios. The resulting distribution of intersection values is expected to follow a Gaussian distribution, and we used the fitted Gaussian to compute a z-score of our observed intersection value for each cell line. We also repeated the whole procedure for only the intergenic regions, defined as regions at least 10,000 bp from any Gencode version 7 level 1 and level 2 genes.

## Abbreviations

ac: acetylation; BAR: binding active region; BIR: binding inactive region; bp: base pair; CAGE: cap-analysis of gene expression; ChIP-seq: chromatin immunoprecipitation followed by sequencing; DRM: gene-distal regulatory module; ENCODE: Encyclopedia of DNA Elements; FAIRE: formaldehyde-assisted isolation of regulatory elements; H3: histone 3; HOT: high occupancy of TRF; K: lysine; LOT: low occupancy of TRF; me: mono-methylation; me2: di-methylation; me3: tri-methylation; PET: paired-end diTag; PRM: promoter-proximal regulatory module; RNA-seq: RNA sequencing; RPM: reads per million mapped reads; RPKM: reads per kilobase per million mapped reads; TF: transcription factor; TRF: transcription-related factor; TSS: transcription start site.

## Competing interests

The authors declare that they have no competing interests.

## Authors' contributions

CC, JR, KYY, MG and MS conceived the study. CC and KYY defined and produced BARs, BIRs, DRMs and PRMs. JBB, KYY, MG, NB and PJB defined and produced HOT and LOT regions. KYY performed feature analysis of the six types of regions. JL and KKY defined and analyzed potential targets of DRMs. AK, CC and KYY performed enhancer predictions. EB, JBB and KYY defined and analyzed motifless regions. KYY and MG wrote the manuscript. All authors read and approved the final manuscript.

## Supplementary Material

Additional file 1 **Supplementary materials**. This file contains supplementary tables, legends of supplementary figures, and information about a supplementary web site. Click here for file

Additional file 2 **Supplementary figures**. This file contains supplementary figures. Click here for file
