# Supplementary material for: Classification of human genomic regions based on experimentally determined binding sites of more than 100 transcription-related factors
Source: Genome Biol. 2012 Sep 5;13(9):R48. doi: 10.1186/gb-2012-13-9-r48 (PMC3491392; doi:10.1186/gb-2012-13-9-r48)
Supplement: Additional file 1 — Supplementary materials. This file contains supplementary tables, legends of supplementary figures, and information about a supplementary web site. [file gb-2012-13-9-r48-S1.pdf]

Supplementary materials

Supplementary tables

Table S1: Cell lines and transcription-related factors (TRFs) involved in the current study.

Cell line		Number of TRFs	List of TRFs in HGNC (HUGO Gene Nomenclature Committee) gene symbols
GM12878	B-lymphoblastoid cell line	51	ATF3, BATF, BCL11A, BCL3, BCLAF1, BRCA1, CHD2, CTCF, EBF1, EGR1, ELF1, EP300, ETS1, FOS, GABPA, IRF4, JUND, MAX, MEF2A, MEF2C, NFE2, NFKB1, NR2C2, NRF1, PAX5, PBX3, POLR2A, POLR3G, POU2F2, RAD21, REST, RFX5, RXRA, SIN3A, SIX5, SMC3, SP1, SPI1, SRF, STAT3, TAF1, TBP, TCF12, USF1, USF2, WRNIP1, YY1, ZBTB33, ZEB1, ZNF143, ZZZ3
H1-hESC	Human embryonic stem cells, line H1	32	ATF3, BCL11A, BCL3, CTBP2, CTCF, EGR1, EP300, GABPA, HDAC2, JUN, JUND, MAX, NANOG, NRF1, POLR2A, POU5F1, RAD21, REST, RFX5, RXRA, SIN3A, SIX5, SP1, SRF, SUZ12, TAF1, TAF7, TBP, TCF12, USF1, USF2, YY1
HeLa-S3	Cervical carcinoma cell line	43	BDP1, BRCA1, BRF1, BRF2, CEBPB, CTCF, E2F1, E2F4, E2F6, ELK4, EP300, FOS, GABPA, GTF3C2, GTF2F1, IRF3, JUN, JUND, MAX, MXI1, MYC, NR2C2, NRF1, POLR2A, POLR3A, PRDM1, RAD21, REST, RFX5, SMARCA4, SMARCB1, SMARCC1, SMARCC2, SMC3, STAT1, STAT3, TAF1, TBP, TFAP2A, TFAP2C, USF2, ZNF274, ZZZ3
Hep-G2	Hepatoblastoma cell line	42	ATF3, BHLHE40, CEBPB, CHD2, CTCF, ELF1, EP300, ESRRA, FOSL2, FOXA1, FOXA2, GABPA, HDAC2, HNF4A, HNF4G, HSF1, JUN, JUND, MAFF, MAFK, MYC, NR2C2, NR3C1, NRF1, POLR2A, PPARGC1A, RAD21, REST, RFX5, RXRA, SIN3A, SP1, SREBF1, SRF, TAF1,

			TBP, TCF12, TCF7L2, USF1, USF2, ZBTB33, ZNF274
K562	Chronic myelogenous/ erythroleukemia cell line	73	ATF3, BCL3, BCLAF1, BDP1, BRF1, BRF2, CCNT2, CEBPB, CHD2, CTCF, CTCFL, E2F4, E2F6, EGR1, ELF1, EP300, ETS1, FOS, FOSL1, GABPA, GATA1, GATA2, GTF2B, GTF2F1, GTF3C2, HDAC2, HDAC8, HMGN3, IRF1, JUN, JUNB, JUND, MAFK, MAX, MXI1, MYC, NFE2, NFYA, NFYB, NR2C2, NRF1, POLR2A, POLR3A, POLR3G, RAD21, RDBP, REST, SETDB1, SIN3A, SIRT6, SIX5, SMARCA4, SMARCB1, SMC3, SP1, SP2, SPI1, SRF, STAT1, STAT2, TAF1, TAF7, TAL1, TBP, THAP1, TRIM28, USF1, USF2, YY1, ZBTB33, ZBTB7A, ZNF263, ZNF274

Table S2: Types of histone modifications included in our dataset. A cell in the table is marked with "Yes" if experimental data for the histone modification represented by that row is available for the cell line represented by that column.

Histone modification	GM12878	H1-hESC	HeLa-S3	Hep-G2	K562
H2az	Yes			Yes	Yes
H3K27ac	Yes	Yes	Yes	Yes	Yes
H3K27me3	Yes		Yes	Yes	Yes
H3K36me3	Yes	Yes	Yes	Yes	Yes
H3K4me1	Yes	Yes	Yes	Yes	Yes
H3K4me2	Yes	Yes	Yes	Yes	Yes
H3K4me3	Yes	Yes	Yes	Yes	Yes
H3K79me2	Yes		Yes	Yes	Yes
H3K9ac	Yes	Yes	Yes	Yes	Yes
H3K9me1					Yes
H3K9me3	Yes				Yes
H4K20me1	Yes	Yes	Yes	Yes	Yes

Table S3: The 50 predicted enhancers for the first round of experimental validation in mouse embryos. The 6 included into the reporter assays are marked with a gray background. Coordinates are in human reference genome build hg19.

Chromosome	Start position	End position

chr2	119067130	119068029
chr2	133011930	133013629
chr2	145253130	145253929
chr2	145338030	145339229
chr2	145340030	145340929
chr2	145353230	145354329
chr2	172957754	172959053
chr3	71257310	71258209
chr3	114170010	114170809
chr3	149212610	149213509
chr3	169385206	169386005
chr4	24473902	24474701
chr4	81082276	81083175
chr4	146857350	146858449
chr5	139088816	139089715
chr5	139090216	139091215
chr5	139487116	139487915
chr7	26524775	26525574
chr7	70037764	70038763
chr7	115452664	115453463
chr7	121968764	121969663
chr8	106604924	106605723
chr8	116463723	116464622
chr9	126596879	126597678
chr9	128521379	128522378
chr10	23487194	23488193
chr10	63546094	63546893
chr10	63663194	63663993
chr10	74007994	74008793
chr10	103484910	103485709
chr11	74951752	74952751
chr11	85906452	85907351
chr11	118308990	118309889
chr12	20704433	20705432
chr12	49452933	49454732
chr12	74564833	74565632
chr14	32953349	32954348

chr14	57476147	57476946
chr14	68773647	68774546
chr15	30515508	30516407
chr15	40574008	40574807
chr17	35084087	35085086
chr17	44269323	44270422
chr18	29542202	29543001
chr19	47613060	47614259
chrX	25008379	25009178
chrX	39965456	39966255

Table S4: Number of bins within EP300 binding peaks at different types of genomic regions. The percentages are computed using the total number of bins in the corresponding regions (e.g., DRMs in the particular cell line for the second column) regardless of EP300 binding as denominators.

	Whole genome	DRMs	DRMs in identified DRM-target transcript pairs			
			Poly A+ RNA-seq	Poly A- RNA-seq	Short RNA-seq	Poly-A+ CAGE
GM12878	27,494 (0.09%)	6,772 (2.7%)	119 (6.4%)	90 (4.4%)	5 (4.2%)	296 (8.8%)
H1-hESC	18,321 (0.06%)	3,902 (2.1%)	24 (1.6%)	42 (2.3%)	14 (5.1%)	50 (2.4%)
HeLa-S3	86,286 (0.28%)	28,688 (11.3%)	133 (17.7%)	95 (11.0%)	1 (1.9%)	75 (18.3%)
Hep-G2 (protocol 1)	68,233 (0.22%)	17,773 (8.4%)	316 (25.6%)	427 (26.2%)	6 (11.3%)	213 (31.3%)
Hep-G2 (protocol 2)	135,551 (0.44%)	36,087 (17.1%)				
K562	12,576 (0.04%)	3,359 (1.5%)	78 (5.4%)	98 (3.8%)	18 (4.5%)	9 (2.0%)

Table S5: List of datasets used in this study. All data files can be downloaded from the stated sub-directories of the following URL: http://hgdownload.cse.ucsc.edu/goldenPath/hg19/encodeDCC/.

Type	Sub-directory	Dataset ID
TRF binding	wgEncodeBroadHistone	wgEncodeBroadHistoneGm12878CtcfStd
TRF binding	wgEncodeBroadHistone	wgEncodeBroadHistoneH1hescCtcfStd
TRF binding	wgEncodeBroadHistone	wgEncodeBroadHistoneHelas3CtcfStd
TRF binding	wgEncodeBroadHistone	wgEncodeBroadHistoneHelas3Pol2bStd
TRF binding	wgEncodeBroadHistone	wgEncodeBroadHistoneHepg2CtcfStd
TRF binding	wgEncodeBroadHistone	wgEncodeBroadHistoneK562CtcfStd
TRF binding	wgEncodeBroadHistone	wgEncodeBroadHistoneK562Pol2bStd
TRF binding	wgEncodeHaibTfbs	wgEncodeHaibTfbsGm12878Atf3Pcr1x

TRF binding	wgEncodeHaibTfbs	wgEncodeHaibTfbsGm12878BatfPcr1x
TRF binding	wgEncodeHaibTfbs	wgEncodeHaibTfbsGm12878Bcl11aPcr1x
TRF binding	wgEncodeHaibTfbs	wgEncodeHaibTfbsGm12878Bcl3Pcr1x
TRF binding	wgEncodeHaibTfbs	wgEncodeHaibTfbsGm12878Bclaf1m33V0416101
TRF binding	wgEncodeHaibTfbs	wgEncodeHaibTfbsGm12878EbfPcr1x
TRF binding	wgEncodeHaibTfbs	wgEncodeHaibTfbsGm12878Ebf1c8Pcr1x
TRF binding	wgEncodeHaibTfbs	wgEncodeHaibTfbsGm12878Egr1V0416101
TRF binding	wgEncodeHaibTfbs	wgEncodeHaibTfbsGm12878Elf1sc631V0416101
TRF binding	wgEncodeHaibTfbs	wgEncodeHaibTfbsGm12878Ets1Pcr1x
TRF binding	wgEncodeHaibTfbs	wgEncodeHaibTfbsGm12878GabpPcr2x
TRF binding	wgEncodeHaibTfbs	wgEncodeHaibTfbsGm12878Irf4Pcr1x
TRF binding	wgEncodeHaibTfbs	wgEncodeHaibTfbsGm12878Irf4m17Pcr1x
TRF binding	wgEncodeHaibTfbs	wgEncodeHaibTfbsGm12878Mef2aPcr1x
TRF binding	wgEncodeHaibTfbs	wgEncodeHaibTfbsGm12878Mef2csc13268Pcr1x
TRF binding	wgEncodeHaibTfbs	wgEncodeHaibTfbsGm12878NrsfPcr2x
TRF binding	wgEncodeHaibTfbs	wgEncodeHaibTfbsGm12878Oct2Pcr1x
TRF binding	wgEncodeHaibTfbs	wgEncodeHaibTfbsGm12878P300Pcr1x
TRF binding	wgEncodeHaibTfbs	wgEncodeHaibTfbsGm12878Pax5c20Pcr1x
TRF binding	wgEncodeHaibTfbs	wgEncodeHaibTfbsGm12878Pax5n19Pcr1x
TRF binding	wgEncodeHaibTfbs	wgEncodeHaibTfbsGm12878Pbx3Pcr1x
TRF binding	wgEncodeHaibTfbs	wgEncodeHaibTfbsGm12878Pol2Pcr2x
TRF binding	wgEncodeHaibTfbs	wgEncodeHaibTfbsGm12878Pol24h8Pcr1x
TRF binding	wgEncodeHaibTfbs	wgEncodeHaibTfbsGm12878Pou2f2Pcr1x
TRF binding	wgEncodeHaibTfbs	wgEncodeHaibTfbsGm12878Pu1Pcr1x
TRF binding	wgEncodeHaibTfbs	wgEncodeHaibTfbsGm12878Rad21V0416101
TRF binding	wgEncodeHaibTfbs	wgEncodeHaibTfbsGm12878RxraPcr1x
TRF binding	wgEncodeHaibTfbs	wgEncodeHaibTfbsGm12878Sin3ak20Pcr1x
TRF binding	wgEncodeHaibTfbs	wgEncodeHaibTfbsGm12878Six5Pcr1x
TRF binding	wgEncodeHaibTfbs	wgEncodeHaibTfbsGm12878Sp1Pcr1x
TRF binding	wgEncodeHaibTfbs	wgEncodeHaibTfbsGm12878SrfPcr2x
TRF binding	wgEncodeHaibTfbs	wgEncodeHaibTfbsGm12878SrfV0416101
TRF binding	wgEncodeHaibTfbs	wgEncodeHaibTfbsGm12878Taf1Pcr1x
TRF binding	wgEncodeHaibTfbs	wgEncodeHaibTfbsGm12878Tcf12Pcr1x
TRF binding	wgEncodeHaibTfbs	wgEncodeHaibTfbsGm12878Usf1Pcr2x
TRF binding	wgEncodeHaibTfbs	wgEncodeHaibTfbsGm12878Yy1V0416101
TRF binding	wgEncodeHaibTfbs	wgEncodeHaibTfbsGm12878Zbtb33Pcr1x
TRF binding	wgEncodeHaibTfbs	wgEncodeHaibTfbsGm12878Zeb1sc25388V0416102

TRF binding	wgEncodeHaibTfbs	wgEncodeHaibTfbsH1hescAtf3Pcr1x
TRF binding	wgEncodeHaibTfbs	wgEncodeHaibTfbsH1hescBcl11aPcr1x
TRF binding	wgEncodeHaibTfbs	wgEncodeHaibTfbsH1hescBcl3Pcr1x
TRF binding	wgEncodeHaibTfbs	wgEncodeHaibTfbsH1hescCtcfsc5916V0416102
TRF binding	wgEncodeHaibTfbs	wgEncodeHaibTfbsH1hescEgr1V0416101
TRF binding	wgEncodeHaibTfbs	wgEncodeHaibTfbsH1hescEgr1V0416102
TRF binding	wgEncodeHaibTfbs	wgEncodeHaibTfbsH1hescGabpPcr1x
TRF binding	wgEncodeHaibTfbs	wgEncodeHaibTfbsH1hescHdac2sc6296V0416102
TRF binding	wgEncodeHaibTfbs	wgEncodeHaibTfbsH1hescJundV0416102
TRF binding	wgEncodeHaibTfbs	wgEncodeHaibTfbsH1hescNanogsc33759V0416102
TRF binding	wgEncodeHaibTfbs	wgEncodeHaibTfbsH1hescNrsfPcr1x
TRF binding	wgEncodeHaibTfbs	wgEncodeHaibTfbsH1hescNrsfV0416102
TRF binding	wgEncodeHaibTfbs	wgEncodeHaibTfbsH1hescP300Pcr1x
TRF binding	wgEncodeHaibTfbs	wgEncodeHaibTfbsH1hescPol2Pcr1x
TRF binding	wgEncodeHaibTfbs	wgEncodeHaibTfbsH1hescPol2V0416102
TRF binding	wgEncodeHaibTfbs	wgEncodeHaibTfbsH1hescPol24h8Pcr1x
TRF binding	wgEncodeHaibTfbs	wgEncodeHaibTfbsH1hescPol24h8V0416102
TRF binding	wgEncodeHaibTfbs	wgEncodeHaibTfbsH1hescPou5f1sc9081V0416102
TRF binding	wgEncodeHaibTfbs	wgEncodeHaibTfbsH1hescRad21V0416102
TRF binding	wgEncodeHaibTfbs	wgEncodeHaibTfbsH1hescRxraV0416102
TRF binding	wgEncodeHaibTfbs	wgEncodeHaibTfbsH1hescSin3ak20Pcr1x
TRF binding	wgEncodeHaibTfbs	wgEncodeHaibTfbsH1hescSix5Pcr1x
TRF binding	wgEncodeHaibTfbs	wgEncodeHaibTfbsH1hescSp1Pcr1x
TRF binding	wgEncodeHaibTfbs	wgEncodeHaibTfbsH1hescSrfPcr1x
TRF binding	wgEncodeHaibTfbs	wgEncodeHaibTfbsH1hescTaf1Pcr1x
TRF binding	wgEncodeHaibTfbs	wgEncodeHaibTfbsH1hescTaf1V0416102
TRF binding	wgEncodeHaibTfbs	wgEncodeHaibTfbsH1hescTaf7sq8V0416102
TRF binding	wgEncodeHaibTfbs	wgEncodeHaibTfbsH1hescTcf12Pcr1x
TRF binding	wgEncodeHaibTfbs	wgEncodeHaibTfbsH1hescUsf1Pcr1x
TRF binding	wgEncodeHaibTfbs	wgEncodeHaibTfbsH1hescYy1c20Pcr1x
TRF binding	wgEncodeHaibTfbs	wgEncodeHaibTfbsHelas3GabpPcr1x
TRF binding	wgEncodeHaibTfbs	wgEncodeHaibTfbsHelas3NrsfPcr1x
TRF binding	wgEncodeHaibTfbs	wgEncodeHaibTfbsHelas3Pol2Pcr1x
TRF binding	wgEncodeHaibTfbs	wgEncodeHaibTfbsHelas3Taf1Pcr1x
TRF binding	wgEncodeHaibTfbs	wgEncodeHaibTfbsHepg2Atf3Pcr1x
TRF binding	wgEncodeHaibTfbs	wgEncodeHaibTfbsHepg2Bhlhe40V0416101
TRF binding	wgEncodeHaibTfbs	wgEncodeHaibTfbsHepg2CtcfV0416101

TRF binding	wgEncodeHaibTfbs	wgEncodeHaibTfbsHepg2Elf1sc631V0416101
TRF binding	wgEncodeHaibTfbs	wgEncodeHaibTfbsHepg2Fosl2Pcr1x
TRF binding	wgEncodeHaibTfbs	wgEncodeHaibTfbsHepg2Foxa1c20Pcr1x
TRF binding	wgEncodeHaibTfbs	wgEncodeHaibTfbsHepg2Foxa1sc101058Pcr1x
TRF binding	wgEncodeHaibTfbs	wgEncodeHaibTfbsHepg2Foxa2sc6554V0416101
TRF binding	wgEncodeHaibTfbs	wgEncodeHaibTfbsHepg2GabpPcr2x
TRF binding	wgEncodeHaibTfbs	wgEncodeHaibTfbsHepg2Hdac2sc6296V0416101
TRF binding	wgEncodeHaibTfbs	wgEncodeHaibTfbsHepg2Hnf4ah171Pcr1x
TRF binding	wgEncodeHaibTfbs	wgEncodeHaibTfbsHepg2Hnf4gsc6558V0416101
TRF binding	wgEncodeHaibTfbs	wgEncodeHaibTfbsHepg2JundPcr1x
TRF binding	wgEncodeHaibTfbs	wgEncodeHaibTfbsHepg2NrsfPcr2x
TRF binding	wgEncodeHaibTfbs	wgEncodeHaibTfbsHepg2P300Pcr1x
TRF binding	wgEncodeHaibTfbs	wgEncodeHaibTfbsHepg2P300V0416101
TRF binding	wgEncodeHaibTfbs	wgEncodeHaibTfbsHepg2Pol2Pcr2x
TRF binding	wgEncodeHaibTfbs	wgEncodeHaibTfbsHepg2Rad21V0416101
TRF binding	wgEncodeHaibTfbs	wgEncodeHaibTfbsHepg2RxraPcr1x
TRF binding	wgEncodeHaibTfbs	wgEncodeHaibTfbsHepg2Sin3ak20Pcr1x
TRF binding	wgEncodeHaibTfbs	wgEncodeHaibTfbsHepg2Sp1Pcr1x
TRF binding	wgEncodeHaibTfbs	wgEncodeHaibTfbsHepg2SrfV0416101
TRF binding	wgEncodeHaibTfbs	wgEncodeHaibTfbsHepg2Taf1Pcr2x
TRF binding	wgEncodeHaibTfbs	wgEncodeHaibTfbsHepg2Tcf12Pcr1x
TRF binding	wgEncodeHaibTfbs	wgEncodeHaibTfbsHepg2Usf1Pcr1x
TRF binding	wgEncodeHaibTfbs	wgEncodeHaibTfbsHepg2Zbtb33Pcr1x
TRF binding	wgEncodeHaibTfbs	wgEncodeHaibTfbsHepg2Zbtb33V0416101
TRF binding	wgEncodeHaibTfbs	wgEncodeHaibTfbsK562Bcl3Pcr1x
TRF binding	wgEncodeHaibTfbs	wgEncodeHaibTfbsK562Bclaf1m33Pcr1x
TRF binding	wgEncodeHaibTfbs	wgEncodeHaibTfbsK562Ctcflsc98982V0416101
TRF binding	wgEncodeHaibTfbs	wgEncodeHaibTfbsK562E2f6h50V0416102
TRF binding	wgEncodeHaibTfbs	wgEncodeHaibTfbsK562Egr1V0416101
TRF binding	wgEncodeHaibTfbs	wgEncodeHaibTfbsK562Elf1sc631V0416102
TRF binding	wgEncodeHaibTfbs	wgEncodeHaibTfbsK562Ets1V0416101
TRF binding	wgEncodeHaibTfbs	wgEncodeHaibTfbsK562Fosl1sc183V0416101
TRF binding	wgEncodeHaibTfbs	wgEncodeHaibTfbsK562GabpV0416101
TRF binding	wgEncodeHaibTfbs	wgEncodeHaibTfbsK562Gata2cg2Pcr1x
TRF binding	wgEncodeHaibTfbs	wgEncodeHaibTfbsK562Hdac2sc6296V0416102
TRF binding	wgEncodeHaibTfbs	wgEncodeHaibTfbsK562MaxV0416102
TRF binding	wgEncodeHaibTfbs	wgEncodeHaibTfbsK562NrsfV0416102

TRF binding	wgEncodeHaibTfbs	wgEncodeHaibTfbsK562Pol2V0416101
TRF binding	wgEncodeHaibTfbs	wgEncodeHaibTfbsK562Pol24h8Pcr1x
TRF binding	wgEncodeHaibTfbs	wgEncodeHaibTfbsK562Pol24h8V0416101
TRF binding	wgEncodeHaibTfbs	wgEncodeHaibTfbsK562Pu1Pcr1x
TRF binding	wgEncodeHaibTfbs	wgEncodeHaibTfbsK562Rad21V0416102
TRF binding	wgEncodeHaibTfbs	wgEncodeHaibTfbsK562Sin3ak20V0416101
TRF binding	wgEncodeHaibTfbs	wgEncodeHaibTfbsK562Six5Pcr1x
TRF binding	wgEncodeHaibTfbs	wgEncodeHaibTfbsK562Sp1Pcr1x
TRF binding	wgEncodeHaibTfbs	wgEncodeHaibTfbsK562Sp2sc643V0416102
TRF binding	wgEncodeHaibTfbs	wgEncodeHaibTfbsK562SrfV0416101
TRF binding	wgEncodeHaibTfbs	wgEncodeHaibTfbsK562Taf1Pcr1x
TRF binding	wgEncodeHaibTfbs	wgEncodeHaibTfbsK562Taf1V0416101
TRF binding	wgEncodeHaibTfbs	wgEncodeHaibTfbsK562Taf7sq8V0416101
TRF binding	wgEncodeHaibTfbs	wgEncodeHaibTfbsK562Thap1sc98174V0416101
TRF binding	wgEncodeHaibTfbs	wgEncodeHaibTfbsK562Usf1V0416101
TRF binding	wgEncodeHaibTfbs	wgEncodeHaibTfbsK562Yy1V0416101
TRF binding	wgEncodeHaibTfbs	wgEncodeHaibTfbsK562Yy1V0416102
TRF binding	wgEncodeHaibTfbs	wgEncodeHaibTfbsK562Zbtb33Pcr1x
TRF binding	wgEncodeHaibTfbs	wgEncodeHaibTfbsK562Zbtb7asc34508V0416101
TRF binding	wgEncodeSydhTfbs	wgEncodeSydhTfbsGm12878Brca1cIggmus
TRF binding	wgEncodeSydhTfbs	wgEncodeSydhTfbsGm12878CfosStd
TRF binding	wgEncodeSydhTfbs	wgEncodeSydhTfbsGm12878Chd21250Iggmus
TRF binding	wgEncodeSydhTfbs	wgEncodeSydhTfbsGm12878Ctcfc20Std
TRF binding	wgEncodeSydhTfbs	wgEncodeSydhTfbsGm12878EbfStd
TRF binding	wgEncodeSydhTfbs	wgEncodeSydhTfbsGm12878JundStd
TRF binding	wgEncodeSydhTfbs	wgEncodeSydhTfbsGm12878MaxStd
TRF binding	wgEncodeSydhTfbs	wgEncodeSydhTfbsGm12878Nfe2hStd
TRF binding	wgEncodeSydhTfbs	wgEncodeSydhTfbsGm12878NfkbIggrab
TRF binding	wgEncodeSydhTfbs	wgEncodeSydhTfbsGm12878Nrf1Iggmus
TRF binding	wgEncodeSydhTfbs	wgEncodeSydhTfbsGm12878Pol2Iggmus
TRF binding	wgEncodeSydhTfbs	wgEncodeSydhTfbsGm12878Pol2Std
TRF binding	wgEncodeSydhTfbs	wgEncodeSydhTfbsGm12878Pol3Std
TRF binding	wgEncodeSydhTfbs	wgEncodeSydhTfbsGm12878Rad21Iggrab
TRF binding	wgEncodeSydhTfbs	wgEncodeSydhTfbsGm12878Rfx5n494Iggmus
TRF binding	wgEncodeSydhTfbs	wgEncodeSydhTfbsGm12878Smc3ab9263Iggmus
TRF binding	wgEncodeSydhTfbs	wgEncodeSydhTfbsGm12878Stat3Iggmus
TRF binding	wgEncodeSydhTfbs	wgEncodeSydhTfbsGm12878TbpIggmus

TRF binding	wgEncodeSydhTfbs	wgEncodeSydhTfbsGm12878Tr4Std
TRF binding	wgEncodeSydhTfbs	wgEncodeSydhTfbsGm12878Usf2Iggmus
TRF binding	wgEncodeSydhTfbs	wgEncodeSydhTfbsGm12878WhipIggmus
TRF binding	wgEncodeSydhTfbs	wgEncodeSydhTfbsGm12878Yy1Std
TRF binding	wgEncodeSydhTfbs	wgEncodeSydhTfbsGm12878Znf143166181apStd
TRF binding	wgEncodeSydhTfbs	wgEncodeSydhTfbsGm12878Zzz3Std
TRF binding	wgEncodeSydhTfbs	wgEncodeSydhTfbsH1hescCjunIggrab
TRF binding	wgEncodeSydhTfbs	wgEncodeSydhTfbsH1hescCtbp2Ucd
TRF binding	wgEncodeSydhTfbs	wgEncodeSydhTfbsH1hescMaxUcd
TRF binding	wgEncodeSydhTfbs	wgEncodeSydhTfbsH1hescNrf1Iggrab
TRF binding	wgEncodeSydhTfbs	wgEncodeSydhTfbsH1hescRad21Iggrab
TRF binding	wgEncodeSydhTfbs	wgEncodeSydhTfbsH1hescRfx5n494Iggrab
TRF binding	wgEncodeSydhTfbs	wgEncodeSydhTfbsH1hescSuz12Ucd
TRF binding	wgEncodeSydhTfbs	wgEncodeSydhTfbsH1hescTbpStd
TRF binding	wgEncodeSydhTfbs	wgEncodeSydhTfbsH1hescUsf2Iggrab
TRF binding	wgEncodeSydhTfbs	wgEncodeSydhTfbsHelas3Ap2alphaStd
TRF binding	wgEncodeSydhTfbs	wgEncodeSydhTfbsHelas3Ap2gammaStd
TRF binding	wgEncodeSydhTfbs	wgEncodeSydhTfbsHelas3Baf155Iggmus
TRF binding	wgEncodeSydhTfbs	wgEncodeSydhTfbsHelas3Baf170Iggmus
TRF binding	wgEncodeSydhTfbs	wgEncodeSydhTfbsHelas3Bdp1Std
TRF binding	wgEncodeSydhTfbs	wgEncodeSydhTfbsHelas3Brca1cIggrab
TRF binding	wgEncodeSydhTfbs	wgEncodeSydhTfbsHelas3Brf1Std
TRF binding	wgEncodeSydhTfbs	wgEncodeSydhTfbsHelas3Brf2Std
TRF binding	wgEncodeSydhTfbs	wgEncodeSydhTfbsHelas3Brg1Iggmus
TRF binding	wgEncodeSydhTfbs	wgEncodeSydhTfbsHelas3CebpbIggrab
TRF binding	wgEncodeSydhTfbs	wgEncodeSydhTfbsHelas3CfosStd
TRF binding	wgEncodeSydhTfbs	wgEncodeSydhTfbsHelas3CjunIggrab
TRF binding	wgEncodeSydhTfbs	wgEncodeSydhTfbsHelas3CmycStd
TRF binding	wgEncodeSydhTfbs	wgEncodeSydhTfbsHelas3E2f1Std
TRF binding	wgEncodeSydhTfbs	wgEncodeSydhTfbsHelas3E2f4Std
TRF binding	wgEncodeSydhTfbs	wgEncodeSydhTfbsHelas3E2f6Std
TRF binding	wgEncodeSydhTfbs	wgEncodeSydhTfbsHelas3Elk4Ucd
TRF binding	wgEncodeSydhTfbs	wgEncodeSydhTfbsHelas3Gtf2f1rapIggrab
TRF binding	wgEncodeSydhTfbs	wgEncodeSydhTfbsHelas3Hae2f1Std
TRF binding	wgEncodeSydhTfbs	wgEncodeSydhTfbsHelas3Ini1Iggmus
TRF binding	wgEncodeSydhTfbs	wgEncodeSydhTfbsHelas3Irf3Iggrab
TRF binding	wgEncodeSydhTfbs	wgEncodeSydhTfbsHelas3JundIggrab

TRF binding	wgEncodeSydhTfbs	wgEncodeSydhTfbsHelas3MaxStd
TRF binding	wgEncodeSydhTfbs	wgEncodeSydhTfbsHelas3Mxi1bhlhIggrab
TRF binding	wgEncodeSydhTfbs	wgEncodeSydhTfbsHelas3Nrf1Iggmus
TRF binding	wgEncodeSydhTfbs	wgEncodeSydhTfbsHelas3P300n15Iggrab
TRF binding	wgEncodeSydhTfbs	wgEncodeSydhTfbsHelas3Pol2Std
TRF binding	wgEncodeSydhTfbs	wgEncodeSydhTfbsHelas3Pol2s2Iggrab
TRF binding	wgEncodeSydhTfbs	wgEncodeSydhTfbsHelas3Prdm1vIggrab
TRF binding	wgEncodeSydhTfbs	wgEncodeSydhTfbsHelas3Rad21Iggrab
TRF binding	wgEncodeSydhTfbs	wgEncodeSydhTfbsHelas3Rfx5n494Iggrab
TRF binding	wgEncodeSydhTfbs	wgEncodeSydhTfbsHelas3Rpc155Std
TRF binding	wgEncodeSydhTfbs	wgEncodeSydhTfbsHelas3Smc3ab9263Iggrab
TRF binding	wgEncodeSydhTfbs	wgEncodeSydhTfbsHelas3Stat1Ifng30Std
TRF binding	wgEncodeSydhTfbs	wgEncodeSydhTfbsHelas3Stat3Iggrab
TRF binding	wgEncodeSydhTfbs	wgEncodeSydhTfbsHelas3TbpIggrab
TRF binding	wgEncodeSydhTfbs	wgEncodeSydhTfbsHelas3Tf3c110Std
TRF binding	wgEncodeSydhTfbs	wgEncodeSydhTfbsHelas3Tr4Std
TRF binding	wgEncodeSydhTfbs	wgEncodeSydhTfbsHelas3Usf2Iggmus
TRF binding	wgEncodeSydhTfbs	wgEncodeSydhTfbsHelas3Znf274Ucd
TRF binding	wgEncodeSydhTfbs	wgEncodeSydhTfbsHelas3Zzz3Std
TRF binding	wgEncodeSydhTfbs	wgEncodeSydhTfbsHepg2CebpbForsklnStd
TRF binding	wgEncodeSydhTfbs	wgEncodeSydhTfbsHepg2CebpbIggrab
TRF binding	wgEncodeSydhTfbs	wgEncodeSydhTfbsHepg2Chd21250Iggrab
TRF binding	wgEncodeSydhTfbs	wgEncodeSydhTfbsHepg2CjunIggrab
TRF binding	wgEncodeSydhTfbs	wgEncodeSydhTfbsHepg2ErraForsklnStd
TRF binding	wgEncodeSydhTfbs	wgEncodeSydhTfbsHepg2Grp20ForsklnStd
TRF binding	wgEncodeSydhTfbs	wgEncodeSydhTfbsHepg2Hnf4aForsklnStd
TRF binding	wgEncodeSydhTfbs	wgEncodeSydhTfbsHepg2Hsf1ForsklnStd
TRF binding	wgEncodeSydhTfbs	wgEncodeSydhTfbsHepg2JundIggrab
TRF binding	wgEncodeSydhTfbs	wgEncodeSydhTfbsHepg2Maffm8194Iggrab
TRF binding	wgEncodeSydhTfbs	wgEncodeSydhTfbsHepg2Mafkab50322Iggrab
TRF binding	wgEncodeSydhTfbs	wgEncodeSydhTfbsHepg2Mafksc477Iggrab
TRF binding	wgEncodeSydhTfbs	wgEncodeSydhTfbsHepg2Nrf1Iggrab
TRF binding	wgEncodeSydhTfbs	wgEncodeSydhTfbsHepg2Pgc1aForsklnStd
TRF binding	wgEncodeSydhTfbs	wgEncodeSydhTfbsHepg2Pol2ForsklnStd
TRF binding	wgEncodeSydhTfbs	wgEncodeSydhTfbsHepg2Pol2Iggrab
TRF binding	wgEncodeSydhTfbs	wgEncodeSydhTfbsHepg2Pol2PravastStd
TRF binding	wgEncodeSydhTfbs	wgEncodeSydhTfbsHepg2Rad21Iggrab

TRF binding	wgEncodeSydhTfbs	wgEncodeSydhTfbsHepg2Rfx5n494Iggrab
TRF binding	wgEncodeSydhTfbs	wgEncodeSydhTfbsHepg2Srebp1InslnStd
TRF binding	wgEncodeSydhTfbs	wgEncodeSydhTfbsHepg2TbpIggrab
TRF binding	wgEncodeSydhTfbs	wgEncodeSydhTfbsHepg2Tcf4Ucd
TRF binding	wgEncodeSydhTfbs	wgEncodeSydhTfbsHepg2Usf2Iggrab
TRF binding	wgEncodeSydhTfbs	wgEncodeSydhTfbsHepg2bTr4Ucd
TRF binding	wgEncodeSydhTfbs	wgEncodeSydhTfbsHepg2bZnf274Ucd
TRF binding	wgEncodeSydhTfbs	wgEncodeSydhTfbsK562Atf3Std
TRF binding	wgEncodeSydhTfbs	wgEncodeSydhTfbsK562Bdp1Std
TRF binding	wgEncodeSydhTfbs	wgEncodeSydhTfbsK562Brf1Std
TRF binding	wgEncodeSydhTfbs	wgEncodeSydhTfbsK562Brf2Std
TRF binding	wgEncodeSydhTfbs	wgEncodeSydhTfbsK562Brg1Iggmus
TRF binding	wgEncodeSydhTfbs	wgEncodeSydhTfbsK562Ccnt2Std
TRF binding	wgEncodeSydhTfbs	wgEncodeSydhTfbsK562CebpbIggrab
TRF binding	wgEncodeSydhTfbs	wgEncodeSydhTfbsK562CfosStd
TRF binding	wgEncodeSydhTfbs	wgEncodeSydhTfbsK562Chd21250Iggrab
TRF binding	wgEncodeSydhTfbs	wgEncodeSydhTfbsK562CjunIfna6hStd
TRF binding	wgEncodeSydhTfbs	wgEncodeSydhTfbsK562CjunIfng30Std
TRF binding	wgEncodeSydhTfbs	wgEncodeSydhTfbsK562CjunIfng6hStd
TRF binding	wgEncodeSydhTfbs	wgEncodeSydhTfbsK562CjunStd
TRF binding	wgEncodeSydhTfbs	wgEncodeSydhTfbsK562CmycIfna30Std
TRF binding	wgEncodeSydhTfbs	wgEncodeSydhTfbsK562CmycIfna6hStd
TRF binding	wgEncodeSydhTfbs	wgEncodeSydhTfbsK562CmycIfng30Std
TRF binding	wgEncodeSydhTfbs	wgEncodeSydhTfbsK562CmycIfng6hStd
TRF binding	wgEncodeSydhTfbs	wgEncodeSydhTfbsK562CmycStd
TRF binding	wgEncodeSydhTfbs	wgEncodeSydhTfbsK562Gtf2bStd
TRF binding	wgEncodeSydhTfbs	wgEncodeSydhTfbsK562Gtf2f1rapIggrab
TRF binding	wgEncodeSydhTfbs	wgEncodeSydhTfbsK562Hmgn3Std
TRF binding	wgEncodeSydhTfbs	wgEncodeSydhTfbsK562Ini1Iggmus
TRF binding	wgEncodeSydhTfbs	wgEncodeSydhTfbsK562Irf1Ifna30Std
TRF binding	wgEncodeSydhTfbs	wgEncodeSydhTfbsK562Irf1Ifng6hStd
TRF binding	wgEncodeSydhTfbs	wgEncodeSydhTfbsK562JundStd
TRF binding	wgEncodeSydhTfbs	wgEncodeSydhTfbsK562Mafkab50322Iggrab
TRF binding	wgEncodeSydhTfbs	wgEncodeSydhTfbsK562MaxStd
TRF binding	wgEncodeSydhTfbs	wgEncodeSydhTfbsK562Mxi1bhlhIggrab
TRF binding	wgEncodeSydhTfbs	wgEncodeSydhTfbsK562NelfeStd
TRF binding	wgEncodeSydhTfbs	wgEncodeSydhTfbsK562Nfe2Std

TRF binding	wgEncodeSydhTfbs	wgEncodeSydhTfbsK562NfyaStd
TRF binding	wgEncodeSydhTfbs	wgEncodeSydhTfbsK562NfybStd
TRF binding	wgEncodeSydhTfbs	wgEncodeSydhTfbsK562Nrf1Iggrab
TRF binding	wgEncodeSydhTfbs	wgEncodeSydhTfbsK562P300f4Iggrab
TRF binding	wgEncodeSydhTfbs	wgEncodeSydhTfbsK562Pol2Ifna30Std
TRF binding	wgEncodeSydhTfbs	wgEncodeSydhTfbsK562Pol2Ifna6hStd
TRF binding	wgEncodeSydhTfbs	wgEncodeSydhTfbsK562Pol2Ifng30Std
TRF binding	wgEncodeSydhTfbs	wgEncodeSydhTfbsK562Pol2Ifng6hStd
TRF binding	wgEncodeSydhTfbs	wgEncodeSydhTfbsK562Pol2Iggmus
TRF binding	wgEncodeSydhTfbs	wgEncodeSydhTfbsK562Pol2Std
TRF binding	wgEncodeSydhTfbs	wgEncodeSydhTfbsK562Pol3Std
TRF binding	wgEncodeSydhTfbs	wgEncodeSydhTfbsK562Rad21Std
TRF binding	wgEncodeSydhTfbs	wgEncodeSydhTfbsK562Rpc155Std
TRF binding	wgEncodeSydhTfbs	wgEncodeSydhTfbsK562Sirt6Std
TRF binding	wgEncodeSydhTfbs	wgEncodeSydhTfbsK562Smc3ab9263Iggrab
TRF binding	wgEncodeSydhTfbs	wgEncodeSydhTfbsK562Stat1Ifna30Std
TRF binding	wgEncodeSydhTfbs	wgEncodeSydhTfbsK562Stat1Ifna6hStd
TRF binding	wgEncodeSydhTfbs	wgEncodeSydhTfbsK562Stat1Ifng30Std
TRF binding	wgEncodeSydhTfbs	wgEncodeSydhTfbsK562Stat1Ifng6hStd
TRF binding	wgEncodeSydhTfbs	wgEncodeSydhTfbsK562Stat2Ifna30Std
TRF binding	wgEncodeSydhTfbs	wgEncodeSydhTfbsK562Stat2Ifna6hStd
TRF binding	wgEncodeSydhTfbs	wgEncodeSydhTfbsK562Tal1sc12984Iggmus
TRF binding	wgEncodeSydhTfbs	wgEncodeSydhTfbsK562TbpIggmus
TRF binding	wgEncodeSydhTfbs	wgEncodeSydhTfbsK562Tf3c110Std
TRF binding	wgEncodeSydhTfbs	wgEncodeSydhTfbsK562Usf2Std
TRF binding	wgEncodeSydhTfbs	wgEncodeSydhTfbsK562bE2f4Ucd
TRF binding	wgEncodeSydhTfbs	wgEncodeSydhTfbsK562bE2f6Ucd
TRF binding	wgEncodeSydhTfbs	wgEncodeSydhTfbsK562bGata1Ucd
TRF binding	wgEncodeSydhTfbs	wgEncodeSydhTfbsK562bGata2Ucd
TRF binding	wgEncodeSydhTfbs	wgEncodeSydhTfbsK562bKap1Ucd
TRF binding	wgEncodeSydhTfbs	wgEncodeSydhTfbsK562bSetdb1MnasedUcd
TRF binding	wgEncodeSydhTfbs	wgEncodeSydhTfbsK562bSetdb1Ucd
TRF binding	wgEncodeSydhTfbs	wgEncodeSydhTfbsK562bTr4Ucd
TRF binding	wgEncodeSydhTfbs	wgEncodeSydhTfbsK562bYy1Ucd
TRF binding	wgEncodeSydhTfbs	wgEncodeSydhTfbsK562bZnf263Ucd
TRF binding	wgEncodeSydhTfbs	wgEncodeSydhTfbsK562bZnf274Ucd
TRF binding	wgEncodeUchicagoTfbs	wgEncodeUchicagoTfbsK562EfosControl

TRF binding	wgEncodeUchicagoTfbs	wgEncodeUchicagoTfbsK562Egata2Control
TRF binding	wgEncodeUchicagoTfbs	wgEncodeUchicagoTfbsK562Ehdac8Control
TRF binding	wgEncodeUchicagoTfbs	wgEncodeUchicagoTfbsK562EjunbControl
TRF binding	wgEncodeUchicagoTfbs	wgEncodeUchicagoTfbsK562EjundControl
TRF binding	wgEncodeOpenChromChip	wgEncodeOpenChromChipGm12878Ctcf
TRF binding	wgEncodeOpenChromChip	wgEncodeOpenChromChipGm12878Pol2
TRF binding	wgEncodeOpenChromChip	wgEncodeOpenChromChipH1hescCmyc
TRF binding	wgEncodeOpenChromChip	wgEncodeOpenChromChipH1hescCtcf
TRF binding	wgEncodeOpenChromChip	wgEncodeOpenChromChipH1hescPol2
TRF binding	wgEncodeOpenChromChip	wgEncodeOpenChromChipHelas3Ctcf
TRF binding	wgEncodeOpenChromChip	wgEncodeOpenChromChipHelas3Pol2
TRF binding	wgEncodeOpenChromChip	wgEncodeOpenChromChipHepg2Cmyc
TRF binding	wgEncodeOpenChromChip	wgEncodeOpenChromChipHepg2Ctcf
TRF binding	wgEncodeOpenChromChip	wgEncodeOpenChromChipHepg2Pol2
TRF binding	wgEncodeOpenChromChip	wgEncodeOpenChromChipK562Cmyc
TRF binding	wgEncodeOpenChromChip	wgEncodeOpenChromChipK562Ctcf
TRF binding	wgEncodeOpenChromChip	wgEncodeOpenChromChipK562Pol2
TRF binding	wgEncodeUwTfbs	wgEncodeUwTfbsGm12878CtcfStd
TRF binding	wgEncodeUwTfbs	wgEncodeUwTfbsHelas3CtcfStd
TRF binding	wgEncodeUwTfbs	wgEncodeUwTfbsHepg2CtcfStd
TRF binding	wgEncodeUwTfbs	wgEncodeUwTfbsK562CtcfStd
Histone modification	wgEncodeBroadHistone	wgEncodeBroadHistoneGm12878H2azStd
Histone modification	wgEncodeBroadHistone	wgEncodeBroadHistoneGm12878H3k27acStd
Histone modification	wgEncodeBroadHistone	wgEncodeBroadHistoneGm12878H3k27me3Std
Histone modification	wgEncodeBroadHistone	wgEncodeBroadHistoneGm12878H3k36me3Std
Histone modification	wgEncodeBroadHistone	wgEncodeBroadHistoneGm12878H3k4me1Std
Histone modification	wgEncodeBroadHistone	wgEncodeBroadHistoneGm12878H3k4me2Std
Histone modification	wgEncodeBroadHistone	wgEncodeBroadHistoneGm12878H3k4me3Std
Histone modification	wgEncodeBroadHistone	wgEncodeBroadHistoneGm12878H3k79me2Std
Histone modification	wgEncodeBroadHistone	wgEncodeBroadHistoneGm12878H3k9acStd
Histone modification	wgEncodeBroadHistone	wgEncodeBroadHistoneGm12878H3k9me3Std
Histone modification	wgEncodeBroadHistone	wgEncodeBroadHistoneGm12878H4k20me1Std
Histone modification	wgEncodeBroadHistone	wgEncodeBroadHistoneH1hescH3k27acStd
Histone modification	wgEncodeBroadHistone	wgEncodeBroadHistoneH1hescH3k27me3Std
Histone modification	wgEncodeBroadHistone	wgEncodeBroadHistoneH1hescH3k36me3Std
Histone modification	wgEncodeBroadHistone	wgEncodeBroadHistoneH1hescH3k4me1Std
Histone modification	wgEncodeBroadHistone	wgEncodeBroadHistoneH1hescH3k4me2Std

Histone modification	wgEncodeBroadHistone	wgEncodeBroadHistoneH1hescH3k4me3Std
Histone modification	wgEncodeBroadHistone	wgEncodeBroadHistoneH1hescH3k9acStd
Histone modification	wgEncodeBroadHistone	wgEncodeBroadHistoneH1hescH4k20me1Std
Histone modification	wgEncodeBroadHistone	wgEncodeBroadHistoneHelas3H3k27acStd
Histone modification	wgEncodeBroadHistone	wgEncodeBroadHistoneHelas3H3k27me3Std
Histone modification	wgEncodeBroadHistone	wgEncodeBroadHistoneHelas3H3k36me3Std
Histone modification	wgEncodeBroadHistone	wgEncodeBroadHistoneHelas3H3k4me1Std
Histone modification	wgEncodeBroadHistone	wgEncodeBroadHistoneHelas3H3k4me2Std
Histone modification	wgEncodeBroadHistone	wgEncodeBroadHistoneHelas3H3k4me3Std
Histone modification	wgEncodeBroadHistone	wgEncodeBroadHistoneHelas3H3k79me2Std
Histone modification	wgEncodeBroadHistone	wgEncodeBroadHistoneHelas3H3k9acStd
Histone modification	wgEncodeBroadHistone	wgEncodeBroadHistoneHelas3H4k20me1Std
Histone modification	wgEncodeBroadHistone	wgEncodeBroadHistoneHepg2H2azStd
Histone modification	wgEncodeBroadHistone	wgEncodeBroadHistoneHepg2H3k27acStd
Histone modification	wgEncodeBroadHistone	wgEncodeBroadHistoneHepg2H3k27me3Std
Histone modification	wgEncodeBroadHistone	wgEncodeBroadHistoneHepg2H3k36me3Std
Histone modification	wgEncodeBroadHistone	wgEncodeBroadHistoneHepg2H3k4me1Std
Histone modification	wgEncodeBroadHistone	wgEncodeBroadHistoneHepg2H3k4me2Std
Histone modification	wgEncodeBroadHistone	wgEncodeBroadHistoneHepg2H3k4me3Std
Histone modification	wgEncodeBroadHistone	wgEncodeBroadHistoneHepg2H3k79me2Std
Histone modification	wgEncodeBroadHistone	wgEncodeBroadHistoneHepg2H3k9acStd
Histone modification	wgEncodeBroadHistone	wgEncodeBroadHistoneHepg2H4k20me1Std
Histone modification	wgEncodeBroadHistone	wgEncodeBroadHistoneHmecH3k27acStd
Histone modification	wgEncodeBroadHistone	wgEncodeBroadHistoneHmecH3k27me3Std
Histone modification	wgEncodeBroadHistone	wgEncodeBroadHistoneHmecH3k36me3Std
Histone modification	wgEncodeBroadHistone	wgEncodeBroadHistoneHmecH3k4me1Std
Histone modification	wgEncodeBroadHistone	wgEncodeBroadHistoneHmecH3k4me2Std
Histone modification	wgEncodeBroadHistone	wgEncodeBroadHistoneHmecH3k4me3Std
Histone modification	wgEncodeBroadHistone	wgEncodeBroadHistoneHmecH3k9acStd
Histone modification	wgEncodeBroadHistone	wgEncodeBroadHistoneHmecH4k20me1Std
Histone modification	wgEncodeBroadHistone	wgEncodeBroadHistoneHsmmH2azStd
Histone modification	wgEncodeBroadHistone	wgEncodeBroadHistoneHsmmH3k27acStd
Histone modification	wgEncodeBroadHistone	wgEncodeBroadHistoneHsmmH3k27me3Std
Histone modification	wgEncodeBroadHistone	wgEncodeBroadHistoneHsmmH3k36me3Std
Histone modification	wgEncodeBroadHistone	wgEncodeBroadHistoneHsmmH3k4me1Std
Histone modification	wgEncodeBroadHistone	wgEncodeBroadHistoneHsmmH3k4me2Std
Histone modification	wgEncodeBroadHistone	wgEncodeBroadHistoneHsmmH3k4me3Std

Histone modification	wgEncodeBroadHistone	wgEncodeBroadHistoneHsmmH3k79me2Std
Histone modification	wgEncodeBroadHistone	wgEncodeBroadHistoneHsmmH3k9acStd
Histone modification	wgEncodeBroadHistone	wgEncodeBroadHistoneHsmmH3k9me3Std
Histone modification	wgEncodeBroadHistone	wgEncodeBroadHistoneHsmmH4k20me1Std
Histone modification	wgEncodeBroadHistone	wgEncodeBroadHistoneHsmmtH2azStd
Histone modification	wgEncodeBroadHistone	wgEncodeBroadHistoneHsmmtH3k27acStd
Histone modification	wgEncodeBroadHistone	wgEncodeBroadHistoneHsmmtH3k36me3Std
Histone modification	wgEncodeBroadHistone	wgEncodeBroadHistoneHsmmtH3k4me1Std
Histone modification	wgEncodeBroadHistone	wgEncodeBroadHistoneHsmmtH3k4me2Std
Histone modification	wgEncodeBroadHistone	wgEncodeBroadHistoneHsmmtH3k4me3Std
Histone modification	wgEncodeBroadHistone	wgEncodeBroadHistoneHsmmtH3k79me2Std
Histone modification	wgEncodeBroadHistone	wgEncodeBroadHistoneHsmmtH3k9acStd
Histone modification	wgEncodeBroadHistone	wgEncodeBroadHistoneHsmmtH4k20me1Std
Histone modification	wgEncodeBroadHistone	wgEncodeBroadHistoneHuvecH3k27acStd
Histone modification	wgEncodeBroadHistone	wgEncodeBroadHistoneHuvecH3k27me3Std
Histone modification	wgEncodeBroadHistone	wgEncodeBroadHistoneHuvecH3k36me3Std
Histone modification	wgEncodeBroadHistone	wgEncodeBroadHistoneHuvecH3k4me1Std
Histone modification	wgEncodeBroadHistone	wgEncodeBroadHistoneHuvecH3k4me2Std
Histone modification	wgEncodeBroadHistone	wgEncodeBroadHistoneHuvecH3k4me3Std
Histone modification	wgEncodeBroadHistone	wgEncodeBroadHistoneHuvecH3k9acStd
Histone modification	wgEncodeBroadHistone	wgEncodeBroadHistoneHuvecH3k9me1Std
Histone modification	wgEncodeBroadHistone	wgEncodeBroadHistoneHuvecH4k20me1Std
Histone modification	wgEncodeBroadHistone	wgEncodeBroadHistoneK562H2azStd
Histone modification	wgEncodeBroadHistone	wgEncodeBroadHistoneK562H3k27acStd
Histone modification	wgEncodeBroadHistone	wgEncodeBroadHistoneK562H3k27me3Std
Histone modification	wgEncodeBroadHistone	wgEncodeBroadHistoneK562H3k36me3Std
Histone modification	wgEncodeBroadHistone	wgEncodeBroadHistoneK562H3k4me1Std
Histone modification	wgEncodeBroadHistone	wgEncodeBroadHistoneK562H3k4me2Std
Histone modification	wgEncodeBroadHistone	wgEncodeBroadHistoneK562H3k4me3Std
Histone modification	wgEncodeBroadHistone	wgEncodeBroadHistoneK562H3k79me2Std
Histone modification	wgEncodeBroadHistone	wgEncodeBroadHistoneK562H3k9acStd
Histone modification	wgEncodeBroadHistone	wgEncodeBroadHistoneK562H3k9me1Std
Histone modification	wgEncodeBroadHistone	wgEncodeBroadHistoneK562H3k9me3Std
Histone modification	wgEncodeBroadHistone	wgEncodeBroadHistoneK562H4k20me1Std
Histone modification	wgEncodeBroadHistone	wgEncodeBroadHistoneNhaH3k27acStd
Histone modification	wgEncodeBroadHistone	wgEncodeBroadHistoneNhaH3k27me3Std
Histone modification	wgEncodeBroadHistone	wgEncodeBroadHistoneNhaH3k36me3Std

Histone modification	wgEncodeBroadHistone	wgEncodeBroadHistoneNhaH3k4me1Std
Histone modification	wgEncodeBroadHistone	wgEncodeBroadHistoneNhaH3k4me3Std
Histone modification	wgEncodeBroadHistone	wgEncodeBroadHistoneNhdfadH3k27acStd
Histone modification	wgEncodeBroadHistone	wgEncodeBroadHistoneNhdfadH3k27me3Std
Histone modification	wgEncodeBroadHistone	wgEncodeBroadHistoneNhdfadH3k36me3Std
Histone modification	wgEncodeBroadHistone	wgEncodeBroadHistoneNhdfadH3k4me2Std
Histone modification	wgEncodeBroadHistone	wgEncodeBroadHistoneNhdfadH3k4me3Std
Histone modification	wgEncodeBroadHistone	wgEncodeBroadHistoneNhdfadH3k9acStd
Histone modification	wgEncodeBroadHistone	wgEncodeBroadHistoneNhekH3k27acStd
Histone modification	wgEncodeBroadHistone	wgEncodeBroadHistoneNhekH3k27me3Std
Histone modification	wgEncodeBroadHistone	wgEncodeBroadHistoneNhekH3k36me3Std
Histone modification	wgEncodeBroadHistone	wgEncodeBroadHistoneNhekH3k4me1Std
Histone modification	wgEncodeBroadHistone	wgEncodeBroadHistoneNhekH3k4me2Std
Histone modification	wgEncodeBroadHistone	wgEncodeBroadHistoneNhekH3k4me3Std
Histone modification	wgEncodeBroadHistone	wgEncodeBroadHistoneNhekH3k9acStd
Histone modification	wgEncodeBroadHistone	wgEncodeBroadHistoneNhekH3k9me1Std
Histone modification	wgEncodeBroadHistone	wgEncodeBroadHistoneNhekH4k20me1Std
Histone modification	wgEncodeBroadHistone	wgEncodeBroadHistoneNhlfH3k27acStd
Histone modification	wgEncodeBroadHistone	wgEncodeBroadHistoneNhlfH3k27me3Std
Histone modification	wgEncodeBroadHistone	wgEncodeBroadHistoneNhlfH3k36me3Std
Histone modification	wgEncodeBroadHistone	wgEncodeBroadHistoneNhlfH3k4me1Std
Histone modification	wgEncodeBroadHistone	wgEncodeBroadHistoneNhlfH3k4me2Std
Histone modification	wgEncodeBroadHistone	wgEncodeBroadHistoneNhlfH3k4me3Std
Histone modification	wgEncodeBroadHistone	wgEncodeBroadHistoneNhlfH3k9acStd
Histone modification	wgEncodeBroadHistone	wgEncodeBroadHistoneNhlfH4k20me1Std
Histone modification	wgEncodeBroadHistone	wgEncodeBroadHistoneOsteoblH2azStd
Histone modification	wgEncodeBroadHistone	wgEncodeBroadHistoneOsteoblH3k27acStd
Histone modification	wgEncodeBroadHistone	wgEncodeBroadHistoneOsteoblH3k36me3Std
Histone modification	wgEncodeBroadHistone	wgEncodeBroadHistoneOsteoblH3k4me1Std
Histone modification	wgEncodeBroadHistone	wgEncodeBroadHistoneOsteoblH3k4me2Std
Histone modification	wgEncodeBroadHistone	wgEncodeBroadHistoneOsteoblH3k9me3Std
Histone modification	wgEncodeSydhHistone	wgEncodeSydhHistoneK562H3k27me3Ucd
Histone modification	wgEncodeSydhHistone	wgEncodeSydhHistoneK562H3k4me1Ucd
Histone modification	wgEncodeSydhHistone	wgEncodeSydhHistoneK562H3k4me3Ucd
Histone modification	wgEncodeSydhHistone	wgEncodeSydhHistoneK562H3k9acbUcd
Histone modification	wgEncodeSydhHistone	wgEncodeSydhHistoneNt2d1H3k27me3Std
Histone modification	wgEncodeSydhHistone	wgEncodeSydhHistoneNt2d1H3k36me3bUcd

Histone modification	wgEncodeSydhHistone	wgEncodeSydhHistoneNt2d1H3k4me1Ucd
Histone modification	wgEncodeSydhHistone	wgEncodeSydhHistoneNt2d1H3k4me3Std
Histone modification	wgEncodeSydhHistone	wgEncodeSydhHistoneNt2d1H3k9acStd
Histone modification	wgEncodeSydhHistone	wgEncodeSydhHistoneNt2d1H3k9me3Ucd
Histone modification	wgEncodeSydhHistone	wgEncodeSydhHistoneU2osH3k9me3Ucd
Histone modification	wgEncodeUwHistone	wgEncodeUwHistoneAg04449H3k4me3Std
Histone modification	wgEncodeUwHistone	wgEncodeUwHistoneAg04450H3k4me3Std
Histone modification	wgEncodeUwHistone	wgEncodeUwHistoneAg09309H3k4me3Std
Histone modification	wgEncodeUwHistone	wgEncodeUwHistoneAg09319H3k4me3Std
Histone modification	wgEncodeUwHistone	wgEncodeUwHistoneAg10803H3k4me3Std
Histone modification	wgEncodeUwHistone	wgEncodeUwHistoneAoafH3k4me3Std
Histone modification	wgEncodeUwHistone	wgEncodeUwHistoneBjH3k27me3Std
Histone modification	wgEncodeUwHistone	wgEncodeUwHistoneBjH3k36me3Std
Histone modification	wgEncodeUwHistone	wgEncodeUwHistoneBjH3k4me3Std
Histone modification	wgEncodeUwHistone	wgEncodeUwHistoneCaco2H3k27me3Std
Histone modification	wgEncodeUwHistone	wgEncodeUwHistoneCaco2H3k36me3Std
Histone modification	wgEncodeUwHistone	wgEncodeUwHistoneCaco2H3k4me3Std
Histone modification	wgEncodeUwHistone	wgEncodeUwHistoneGm06990H3k27me3Std
Histone modification	wgEncodeUwHistone	wgEncodeUwHistoneGm06990H3k36me3Std
Histone modification	wgEncodeUwHistone	wgEncodeUwHistoneGm06990H3k4me3Std
Histone modification	wgEncodeUwHistone	wgEncodeUwHistoneGm12878H3k27me3Std
Histone modification	wgEncodeUwHistone	wgEncodeUwHistoneGm12878H3k36me3Std
Histone modification	wgEncodeUwHistone	wgEncodeUwHistoneGm12878H3k4me3Std
Histone modification	wgEncodeUwHistone	wgEncodeUwHistoneH7esH3k27me3Std
Histone modification	wgEncodeUwHistone	wgEncodeUwHistoneH7esH3k36me3Std
Histone modification	wgEncodeUwHistone	wgEncodeUwHistoneH7esH3k4me3Std
Histone modification	wgEncodeUwHistone	wgEncodeUwHistoneHaspH3k4me3Std
Histone modification	wgEncodeUwHistone	wgEncodeUwHistoneHbmecH3k4me3Std
Histone modification	wgEncodeUwHistone	wgEncodeUwHistoneHcfH3k4me3Std
Histone modification	wgEncodeUwHistone	wgEncodeUwHistoneHcfaaH3k4me3Std
Histone modification	wgEncodeUwHistone	wgEncodeUwHistoneHcmH3k4me3Std
Histone modification	wgEncodeUwHistone	wgEncodeUwHistoneHcpeH3k4me3Std
Histone modification	wgEncodeUwHistone	wgEncodeUwHistoneHct116H3k4me3Std
Histone modification	wgEncodeUwHistone	wgEncodeUwHistoneHeeH3k4me3Std
Histone modification	wgEncodeUwHistone	wgEncodeUwHistoneHek293H3k4me3Std
Histone modification	wgEncodeUwHistone	wgEncodeUwHistoneHelas3H3k27me3Std
Histone modification	wgEncodeUwHistone	wgEncodeUwHistoneHelas3H3k36me3Std

Histone modification	wgEncodeUwHistone	wgEncodeUwHistoneHelas3H3k4me3Std
Histone modification	wgEncodeUwHistone	wgEncodeUwHistoneHepg2H3k27me3Std
Histone modification	wgEncodeUwHistone	wgEncodeUwHistoneHepg2H3k36me3Std
Histone modification	wgEncodeUwHistone	wgEncodeUwHistoneHepg2H3k4me3Std
Histone modification	wgEncodeUwHistone	wgEncodeUwHistoneHl60H3k4me3Std
Histone modification	wgEncodeUwHistone	wgEncodeUwHistoneHmecH3k27me3Std
Histone modification	wgEncodeUwHistone	wgEncodeUwHistoneHmecH3k4me3Std
Histone modification	wgEncodeUwHistone	wgEncodeUwHistoneHmfH3k4me3Std
Histone modification	wgEncodeUwHistone	wgEncodeUwHistoneHpafH3k4me3Std
Histone modification	wgEncodeUwHistone	wgEncodeUwHistoneHpfH3k4me3Std
Histone modification	wgEncodeUwHistone	wgEncodeUwHistoneHreH3k27me3Std
Histone modification	wgEncodeUwHistone	wgEncodeUwHistoneHreH3k36me3Std
Histone modification	wgEncodeUwHistone	wgEncodeUwHistoneHreH3k4me3Std
Histone modification	wgEncodeUwHistone	wgEncodeUwHistoneHrpeH3k4me3Std
Histone modification	wgEncodeUwHistone	wgEncodeUwHistoneHuvecH3k27me3Std
Histone modification	wgEncodeUwHistone	wgEncodeUwHistoneHuvecH3k36me3Std
Histone modification	wgEncodeUwHistone	wgEncodeUwHistoneHuvecH3k4me3Std
Histone modification	wgEncodeUwHistone	wgEncodeUwHistoneHvmfH3k4me3Std
Histone modification	wgEncodeUwHistone	wgEncodeUwHistoneJurkatH3k4me3Std
Histone modification	wgEncodeUwHistone	wgEncodeUwHistoneK562H3k27me3Std
Histone modification	wgEncodeUwHistone	wgEncodeUwHistoneK562H3k36me3Std
Histone modification	wgEncodeUwHistone	wgEncodeUwHistoneK562H3k4me3Std
Histone modification	wgEncodeUwHistone	wgEncodeUwHistoneMcf7H3k4me3Std
Histone modification	wgEncodeUwHistone	wgEncodeUwHistoneNb4H3k4me3Std
Histone modification	wgEncodeUwHistone	wgEncodeUwHistoneNhdfneoH3k4me3Std
Histone modification	wgEncodeUwHistone	wgEncodeUwHistoneNhekH3k27me3Std
Histone modification	wgEncodeUwHistone	wgEncodeUwHistoneNhekH3k36me3Std
Histone modification	wgEncodeUwHistone	wgEncodeUwHistoneNhekH3k4me3Std
Histone modification	wgEncodeUwHistone	wgEncodeUwHistoneSaecH3k27me3Std
Histone modification	wgEncodeUwHistone	wgEncodeUwHistoneSaecH3k36me3Std
Histone modification	wgEncodeUwHistone	wgEncodeUwHistoneSaecH3k4me3Std
Histone modification	wgEncodeUwHistone	wgEncodeUwHistoneSknshraH3k27me3Std
Histone modification	wgEncodeUwHistone	wgEncodeUwHistoneSknshraH3k36me3Std
Histone modification	wgEncodeUwHistone	wgEncodeUwHistoneSknshraH3k4me3Std
Open chromatin	wgEncodeOpenChromDnase	wgEncodeOpenChromDnaseGm12878
Open chromatin	wgEncodeOpenChromDnase	wgEncodeOpenChromDnaseH1hesc
Open chromatin	wgEncodeOpenChromDnase	wgEncodeOpenChromDnaseHelas3

Open chromatin	wgEncodeOpenChromDnase	wgEncodeOpenChromDnaseHelas3Ifna4h
Open chromatin	wgEncodeOpenChromDnase	wgEncodeOpenChromDnaseHepg2
Open chromatin	wgEncodeOpenChromDnase	wgEncodeOpenChromDnaseK562
Open chromatin	wgEncodeOpenChromFaire	wgEncodeOpenChromFaireGm12878
Open chromatin	wgEncodeOpenChromFaire	wgEncodeOpenChromFaireH1hesc
Open chromatin	wgEncodeOpenChromFaire	wgEncodeOpenChromFaireHelas3
Open chromatin	wgEncodeOpenChromFaire	wgEncodeOpenChromFaireHelas3Ifna4h
Open chromatin	wgEncodeOpenChromFaire	wgEncodeOpenChromFaireHelas3Ifng4h
Open chromatin	wgEncodeOpenChromFaire	wgEncodeOpenChromFaireHepg2
Open chromatin	wgEncodeOpenChromFaire	wgEncodeOpenChromFaireK562
Open chromatin	wgEncodeOpenChromFaire	wgEncodeOpenChromFaireK562Nabut
Open chromatin	wgEncodeOpenChromFaire	wgEncodeOpenChromFaireK562Ohurea
Open chromatin	wgEncodeUwDnase	wgEncodeUwDnaseGm12878
Open chromatin	wgEncodeUwDnase	wgEncodeUwDnaseH1hesc
Open chromatin	wgEncodeUwDnase	wgEncodeUwDnaseHelas3
Open chromatin	wgEncodeUwDnase	wgEncodeUwDnaseHepg2
Open chromatin	wgEncodeUwDnase	wgEncodeUwDnaseK562
Expression	wgEncodeRikenCage	wgEncodeRikenCageA549CellPap
Expression	wgEncodeRikenCage	wgEncodeRikenCageAg04450CellPap
Expression	wgEncodeRikenCage	wgEncodeRikenCageBjCellPap
Expression	wgEncodeRikenCage	wgEncodeRikenCageGm12878CellPap
Expression	wgEncodeRikenCage	wgEncodeRikenCageGm12878CytosolPam
Expression	wgEncodeRikenCage	wgEncodeRikenCageGm12878CytosolPap
Expression	wgEncodeRikenCage	wgEncodeRikenCageGm12878NucleolusTotal
Expression	wgEncodeRikenCage	wgEncodeRikenCageGm12878NucleusPam
Expression	wgEncodeRikenCage	wgEncodeRikenCageGm12878NucleusPap
Expression	wgEncodeRikenCage	wgEncodeRikenCageH1hescCellPam
Expression	wgEncodeRikenCage	wgEncodeRikenCageH1hescCellPap
Expression	wgEncodeRikenCage	wgEncodeRikenCageH1hescCytosolPap
Expression	wgEncodeRikenCage	wgEncodeRikenCageH1hescNucleusPap
Expression	wgEncodeRikenCage	wgEncodeRikenCageHelas3CellPap
Expression	wgEncodeRikenCage	wgEncodeRikenCageHelas3CytosolPam
Expression	wgEncodeRikenCage	wgEncodeRikenCageHelas3CytosolPap
Expression	wgEncodeRikenCage	wgEncodeRikenCageHelas3NucleolusTotal
Expression	wgEncodeRikenCage	wgEncodeRikenCageHelas3NucleusPap
Expression	wgEncodeRikenCage	wgEncodeRikenCageHepg2CellPap
Expression	wgEncodeRikenCage	wgEncodeRikenCageHepg2CytosolPam

Expression	wgEncodeRikenCage	wgEncodeRikenCageHepg2CytosolPap
Expression	wgEncodeRikenCage	wgEncodeRikenCageHepg2NucleolusTotal
Expression	wgEncodeRikenCage	wgEncodeRikenCageHepg2NucleusPam
Expression	wgEncodeRikenCage	wgEncodeRikenCageHepg2NucleusPap
Expression	wgEncodeRikenCage	wgEncodeRikenCageHuvecCellPap
Expression	wgEncodeRikenCage	wgEncodeRikenCageHuvecCytosolPam
Expression	wgEncodeRikenCage	wgEncodeRikenCageHuvecCytosolPap
Expression	wgEncodeRikenCage	wgEncodeRikenCageHuvecNucleusPap
Expression	wgEncodeRikenCage	wgEncodeRikenCageK562CellPap
Expression	wgEncodeRikenCage	wgEncodeRikenCageK562ChromatinTotal
Expression	wgEncodeRikenCage	wgEncodeRikenCageK562CytosolPam
Expression	wgEncodeRikenCage	wgEncodeRikenCageK562CytosolPap
Expression	wgEncodeRikenCage	wgEncodeRikenCageK562NucleolusTotal
Expression	wgEncodeRikenCage	wgEncodeRikenCageK562NucleoplasmTotal
Expression	wgEncodeRikenCage	wgEncodeRikenCageK562NucleusPam
Expression	wgEncodeRikenCage	wgEncodeRikenCageK562NucleusPap
Expression	wgEncodeRikenCage	wgEncodeRikenCageK562PolysomePam
Expression	wgEncodeRikenCage	wgEncodeRikenCageMcf7CellPap
Expression	wgEncodeRikenCage	wgEncodeRikenCageNhekCellPap
Expression	wgEncodeRikenCage	wgEncodeRikenCageNhekCytosolPam
Expression	wgEncodeRikenCage	wgEncodeRikenCageNhekCytosolPap
Expression	wgEncodeRikenCage	wgEncodeRikenCageNhekNucleusPam
Expression	wgEncodeRikenCage	wgEncodeRikenCageNhekNucleusPap
Expression	wgEncodeRikenCage	wgEncodeRikenCageProstateCellPam
Expression	wgEncodeRikenCage	wgEncodeRikenCageSknshCellPap
Expression	wgEncodeGisRnaPet	wgEncodeGisRnaPetGm12878CytosolPap
Expression	wgEncodeGisRnaPet	wgEncodeGisRnaPetGm12878NucleusPap
Expression	wgEncodeGisRnaPet	wgEncodeGisRnaPetHepg2CytosolPap
Expression	wgEncodeGisRnaPet	wgEncodeGisRnaPetHepg2NucleusPap
Expression	wgEncodeGisRnaPet	wgEncodeGisRnaPetHuvecCytosolPap
Expression	wgEncodeGisRnaPet	wgEncodeGisRnaPetHuvecNucleusPap
Expression	wgEncodeGisRnaPet	wgEncodeGisRnaPetK562ChromatinTotal
Expression	wgEncodeGisRnaPet	wgEncodeGisRnaPetK562CytosolPap
Expression	wgEncodeGisRnaPet	wgEncodeGisRnaPetK562NucleolusTotal
Expression	wgEncodeGisRnaPet	wgEncodeGisRnaPetK562NucleoplasmTotal
Expression	wgEncodeGisRnaPet	wgEncodeGisRnaPetK562NucleusPap
Expression	wgEncodeGisRnaPet	wgEncodeGisRnaPetK562PolysomePap

Expression	wgEncodeGisRnaPet	wgEncodeGisRnaPetNhekCytosolPap
Expression	wgEncodeGisRnaPet	wgEncodeGisRnaPetNhekNucleusPap
Expression	wgEncodeGisRnaPet	wgEncodeGisRnaPetProstateCellPap
Expression	wgEncodeCshlShortRnaSeq	wgEncodeCshlShortRnaSeqA549CellShorttotalTap
Expression	wgEncodeCshlShortRnaSeq	wgEncodeCshlShortRnaSeqA549CellShorttotalTap
Expression	wgEncodeCshlShortRnaSeq	wgEncodeCshlShortRnaSeqAg04450CellShorttotalTap
Expression	wgEncodeCshlShortRnaSeq	wgEncodeCshlShortRnaSeqBjCellShorttotalTap
Expression	wgEncodeCshlShortRnaSeq	wgEncodeCshlShortRnaSeqGm12878CellShort
Expression	wgEncodeCshlShortRnaSeq	wgEncodeCshlShortRnaSeqGm12878CytosolShort
Expression	wgEncodeCshlShortRnaSeq	wgEncodeCshlShortRnaSeqGm12878NucleusShort
Expression	wgEncodeCshlShortRnaSeq	wgEncodeCshlShortRnaSeqH1hescCellShorttotalTap
Expression	wgEncodeCshlShortRnaSeq	wgEncodeCshlShortRnaSeqH1hescCytosolShorttotalTap
Expression	wgEncodeCshlShortRnaSeq	wgEncodeCshlShortRnaSeqH1hescNucleusShorttotalTap
Expression	wgEncodeCshlShortRnaSeq	wgEncodeCshlShortRnaSeqHelas3CellShorttotalTap
Expression	wgEncodeCshlShortRnaSeq	wgEncodeCshlShortRnaSeqHelas3CytosolShorttotalTap
Expression	wgEncodeCshlShortRnaSeq	wgEncodeCshlShortRnaSeqHelas3NucleusShorttotalTap
Expression	wgEncodeCshlShortRnaSeq	wgEncodeCshlShortRnaSeqHepg2CellShorttotalTap
Expression	wgEncodeCshlShortRnaSeq	wgEncodeCshlShortRnaSeqHepg2CytosolShorttotalTap
Expression	wgEncodeCshlShortRnaSeq	wgEncodeCshlShortRnaSeqHepg2NucleusShorttotalTap
Expression	wgEncodeCshlShortRnaSeq	wgEncodeCshlShortRnaSeqHuvecCellShorttotalTap
Expression	wgEncodeCshlShortRnaSeq	wgEncodeCshlShortRnaSeqHuvecCytosolShorttotalTap
Expression	wgEncodeCshlShortRnaSeq	wgEncodeCshlShortRnaSeqHuvecNucleusShorttotalTap
Expression	wgEncodeCshlShortRnaSeq	wgEncodeCshlShortRnaSeqK562CellShort
Expression	wgEncodeCshlShortRnaSeq	wgEncodeCshlShortRnaSeqK562ChromatinShort
Expression	wgEncodeCshlShortRnaSeq	wgEncodeCshlShortRnaSeqK562CytosolShort
Expression	wgEncodeCshlShortRnaSeq	wgEncodeCshlShortRnaSeqK562NucleolusShort
Expression	wgEncodeCshlShortRnaSeq	wgEncodeCshlShortRnaSeqK562NucleoplasmShort
Expression	wgEncodeCshlShortRnaSeq	wgEncodeCshlShortRnaSeqK562NucleusShort
Expression	wgEncodeCshlShortRnaSeq	wgEncodeCshlShortRnaSeqK562PolysomeShort
Expression	wgEncodeCshlShortRnaSeq	wgEncodeCshlShortRnaSeqMcf7CellShorttotalTap
Expression	wgEncodeCshlShortRnaSeq	wgEncodeCshlShortRnaSeqNhekCellShorttotalTap
Expression	wgEncodeCshlShortRnaSeq	wgEncodeCshlShortRnaSeqNhekCytosolShorttotalTap
Expression	wgEncodeCshlShortRnaSeq	wgEncodeCshlShortRnaSeqNhekNucleusShorttotalTap
Expression	wgEncodeCshlShortRnaSeq	wgEncodeCshlShortRnaSeqProstateCellTotal
Expression	wgEncodeCshlShortRnaSeq	wgEncodeCshlShortRnaSeqSknshraCellShorttotalTap
Expression	wgEncodeCshlLongRnaSeq	wgEncodeCshlLongRnaSeqA549CellLongnonpolya
Expression	wgEncodeCshlLongRnaSeq	wgEncodeCshlLongRnaSeqA549CellPap

Expression	wgEncodeCshlLongRnaSeq	wgEncodeCshlLongRnaSeqAg04450CellLongnonpolya
Expression	wgEncodeCshlLongRnaSeq	wgEncodeCshlLongRnaSeqAg04450CellPap
Expression	wgEncodeCshlLongRnaSeq	wgEncodeCshlLongRnaSeqBjCellLongnonpolya
Expression	wgEncodeCshlLongRnaSeq	wgEncodeCshlLongRnaSeqBjCellPap
Expression	wgEncodeCshlLongRnaSeq	wgEncodeCshlLongRnaSeqGm12878CellLongnonpolya
Expression	wgEncodeCshlLongRnaSeq	wgEncodeCshlLongRnaSeqGm12878CellPap
Expression	wgEncodeCshlLongRnaSeq	wgEncodeCshlLongRnaSeqGm12878CytosolLongnonpolya
Expression	wgEncodeCshlLongRnaSeq	wgEncodeCshlLongRnaSeqGm12878CytosolPap
Expression	wgEncodeCshlLongRnaSeq	wgEncodeCshlLongRnaSeqGm12878NucleusLongnonpolya
Expression	wgEncodeCshlLongRnaSeq	wgEncodeCshlLongRnaSeqGm12878NucleusPap
Expression	wgEncodeCshlLongRnaSeq	wgEncodeCshlLongRnaSeqH1hescCellLongnonpolya
Expression	wgEncodeCshlLongRnaSeq	wgEncodeCshlLongRnaSeqH1hescCellPap
Expression	wgEncodeCshlLongRnaSeq	wgEncodeCshlLongRnaSeqH1hescCytosolLongnonpolya
Expression	wgEncodeCshlLongRnaSeq	wgEncodeCshlLongRnaSeqH1hescCytosolPap
Expression	wgEncodeCshlLongRnaSeq	wgEncodeCshlLongRnaSeqH1hescNucleusLongnonpolya
Expression	wgEncodeCshlLongRnaSeq	wgEncodeCshlLongRnaSeqH1hescNucleusPap
Expression	wgEncodeCshlLongRnaSeq	wgEncodeCshlLongRnaSeqHelas3CellLongnonpolya
Expression	wgEncodeCshlLongRnaSeq	wgEncodeCshlLongRnaSeqHelas3CellPap
Expression	wgEncodeCshlLongRnaSeq	wgEncodeCshlLongRnaSeqHelas3CytosolLongnonpolya
Expression	wgEncodeCshlLongRnaSeq	wgEncodeCshlLongRnaSeqHelas3CytosolPap
Expression	wgEncodeCshlLongRnaSeq	wgEncodeCshlLongRnaSeqHelas3NucleusLongnonpolya
Expression	wgEncodeCshlLongRnaSeq	wgEncodeCshlLongRnaSeqHelas3NucleusPap
Expression	wgEncodeCshlLongRnaSeq	wgEncodeCshlLongRnaSeqHepg2CellLongnonpolya
Expression	wgEncodeCshlLongRnaSeq	wgEncodeCshlLongRnaSeqHepg2CellPap
Expression	wgEncodeCshlLongRnaSeq	wgEncodeCshlLongRnaSeqHepg2CytosolLongnonpolya
Expression	wgEncodeCshlLongRnaSeq	wgEncodeCshlLongRnaSeqHepg2CytosolPap
Expression	wgEncodeCshlLongRnaSeq	wgEncodeCshlLongRnaSeqHepg2NucleusLongnonpolya
Expression	wgEncodeCshlLongRnaSeq	wgEncodeCshlLongRnaSeqHepg2NucleusPap
Expression	wgEncodeCshlLongRnaSeq	wgEncodeCshlLongRnaSeqHmecCellLongnonpolya
Expression	wgEncodeCshlLongRnaSeq	wgEncodeCshlLongRnaSeqHmecCellPap
Expression	wgEncodeCshlLongRnaSeq	wgEncodeCshlLongRnaSeqHsmmCellLongnonpolya
Expression	wgEncodeCshlLongRnaSeq	wgEncodeCshlLongRnaSeqHsmmCellPap
Expression	wgEncodeCshlLongRnaSeq	wgEncodeCshlLongRnaSeqHuvecCellLongnonpolya
Expression	wgEncodeCshlLongRnaSeq	wgEncodeCshlLongRnaSeqHuvecCellPap
Expression	wgEncodeCshlLongRnaSeq	wgEncodeCshlLongRnaSeqHuvecCytosolLongnonpolya
Expression	wgEncodeCshlLongRnaSeq	wgEncodeCshlLongRnaSeqHuvecCytosolPap
Expression	wgEncodeCshlLongRnaSeq	wgEncodeCshlLongRnaSeqHuvecNucleusLongnonpolya

Expression	wgEncodeCshlLongRnaSeq	wgEncodeCshlLongRnaSeqHuvecNucleusPap
Expression	wgEncodeCshlLongRnaSeq	wgEncodeCshlLongRnaSeqK562CellLongnonpolya
Expression	wgEncodeCshlLongRnaSeq	wgEncodeCshlLongRnaSeqK562CellPap
Expression	wgEncodeCshlLongRnaSeq	wgEncodeCshlLongRnaSeqK562ChromatinTotal
Expression	wgEncodeCshlLongRnaSeq	wgEncodeCshlLongRnaSeqK562CytosolLongnonpolya
Expression	wgEncodeCshlLongRnaSeq	wgEncodeCshlLongRnaSeqK562CytosolPap
Expression	wgEncodeCshlLongRnaSeq	wgEncodeCshlLongRnaSeqK562NucleolusTotal
Expression	wgEncodeCshlLongRnaSeq	wgEncodeCshlLongRnaSeqK562NucleoplasmTotal
Expression	wgEncodeCshlLongRnaSeq	wgEncodeCshlLongRnaSeqK562NucleusLongnonpolya
Expression	wgEncodeCshlLongRnaSeq	wgEncodeCshlLongRnaSeqK562NucleusPap
Expression	wgEncodeCshlLongRnaSeq	wgEncodeCshlLongRnaSeqMcf7CellLongnonpolya
Expression	wgEncodeCshlLongRnaSeq	wgEncodeCshlLongRnaSeqMcf7CellPap
Expression	wgEncodeCshlLongRnaSeq	wgEncodeCshlLongRnaSeqNhekCellLongnonpolya
Expression	wgEncodeCshlLongRnaSeq	wgEncodeCshlLongRnaSeqNhekCellPap
Expression	wgEncodeCshlLongRnaSeq	wgEncodeCshlLongRnaSeqNhekCytosolLongnonpolya
Expression	wgEncodeCshlLongRnaSeq	wgEncodeCshlLongRnaSeqNhekCytosolPap
Expression	wgEncodeCshlLongRnaSeq	wgEncodeCshlLongRnaSeqNhekNucleusLongnonpolya
Expression	wgEncodeCshlLongRnaSeq	wgEncodeCshlLongRnaSeqNhekNucleusPap
Expression	wgEncodeCshlLongRnaSeq	wgEncodeCshlLongRnaSeqNhlfCellLongnonpolya
Expression	wgEncodeCshlLongRnaSeq	wgEncodeCshlLongRnaSeqNhlfCellPap
Expression	wgEncodeCshlLongRnaSeq	wgEncodeCshlLongRnaSeqSknshraCellLongnonpolya
Expression	wgEncodeCshlLongRnaSeq	wgEncodeCshlLongRnaSeqSknshraCellPap

Supplementary figure legends

Figure S1: Cross-validation accuracy of the models learned for predicting binding active regions (BARs).

Figure S2: Predicted BAR scores of 1) the positive examples (regions covered by TRF binding peaks) not involved in model training, 2) the negative examples (other random regions), and 3) all bins in the whole genome.

Figure S3: Cross-validation accuracy of the models learned for predicting promoter-proximal regulatory modules (PRMs).

Figure S4: Predicted PRM scores of 1) the positive examples (TSSs of expressed annotated genes) not involved in model training, 2) the negative examples (distal

regions with TRF binding and random regions with no TRF binding), and 3) all bins in the whole genome.

Figure S5: Histograms of the degree of region-specific TRF co-occurrence of all regions in the human genome.

Figure S6: Densities of the six types of regions in the whole genome in GM12878 (A), H1-hESC (B), HeLa-S3 (C), Hep-G2 (D) and K562 (E), defined as the running fractions of bases covered by the regions. The tracks are respectively, from the outermost one to the innermost one, the ideogram for the human karyotype (genome build hg19), Gencode version 7 level 1 and level 2 genes, BARs, BIRs, PRMs, DRMs, HOT regions and LOT regions. The tracks are scaled separately to show density fluctuations.

Figure S7: Distribution of the DRMs in GM12878 (A), H1-hESC (B), HeLa-S3 (C), Hep-G2 (D) and K562 (E). The tracks are respectively, from the outermost one to the innermost one, the ideogram for the human karyotype (genome build hg19), Gencode version 7 level 1 and level 2 genes, regions in GM12878, H1-hESC, HeLa-S3, Hep-G2 and K562. The five innermost tracks in each panel are all in the same scale.

Figure S8: Distributions of open chromatin, histone modification and TRF binding signals at different types of regions in GM12878 (A), H1-hESC (B), HeLa-S3 (C), Hep-G2 (D) and K562 (E). The title of each sub-plot corresponds to the ID of the dataset, in the format <Data source>.<Experiment type>.<Cell line>.<Open chromatin method/ histone modification/ TRF>.<Experiment details>. The dot in each box-and-whisker plot is the average value. Some outlier values are not shown. See Methods for details.

Figure S9: Fraction of TRF binding peaks intersecting the six types of regions in GM12878 (A), H1-hESC (B), HeLa-S3 (C), Hep-G2 (D) and K562 (E). In each panel, the eight rows correspond to, from top to bottom, BARs, BIRs, PRMs, DRMs, HOT regions, LOT regions, intergenic HOT regions and intergenic LOT regions, respectively.

Figure S10: Pipeline for associating DRMs with potential target genes, and TRFs involved. The whole pipeline consists of four main modules. The first and second modules perform filtering of DRMs and transcripts, respectively (A). The third module correlates histone modification signals at the DRMs with the expression

levels of transcripts across different cell lines (B). For the resulting DRM-transcript pairs, the fourth module associates TRFs that are potentially involved (C).

Supplementary files

Supplementary files can be downloaded at the supplementary web site: http://metatracks.encodenets.gersteinlab.org. Detailed descriptions of each file are provided there.
