# Supplementary material for: Classification of human genomic regions based on experimentally determined binding sites of more than 100 transcription-related factors
Source: Genome Biol. 2012 Sep 5;13(9):R48. doi: 10.1186/gb-2012-13-9-r48 (PMC3491392; doi:10.1186/gb-2012-13-9-r48)
Supplement: Additional file 2 — Supplementary figures. This file contains supplementary figures. [file gb-2012-13-9-r48-S2.pdf]

Figure S1

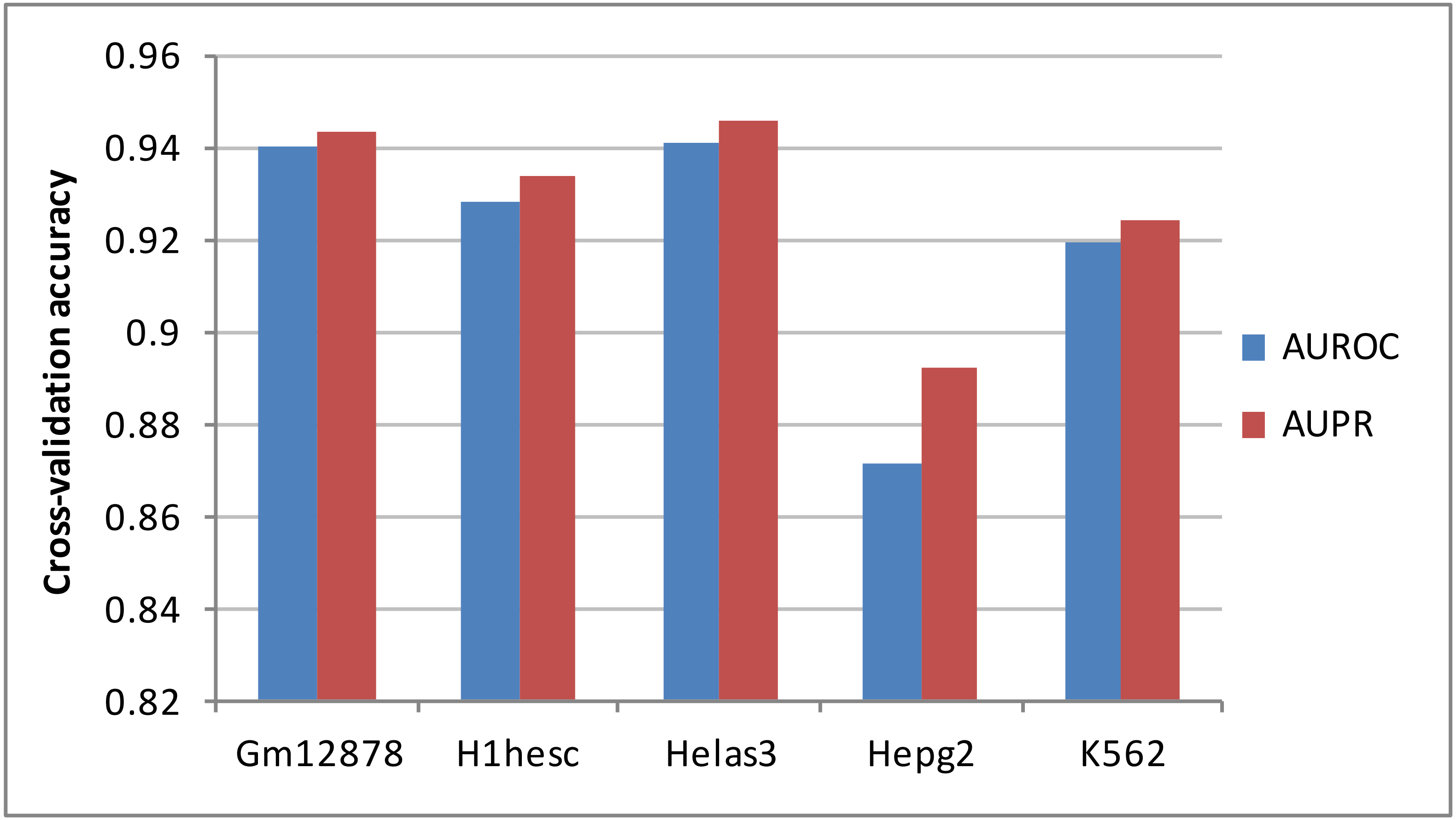
0.96
0.94
0.92
0.9
0.88
0.86
0.84
0.82
Cross-validation accuracy
Gm12878
H1hesc
Helas3
Hepg2
K562
AUROC
AUPR

Figure S2

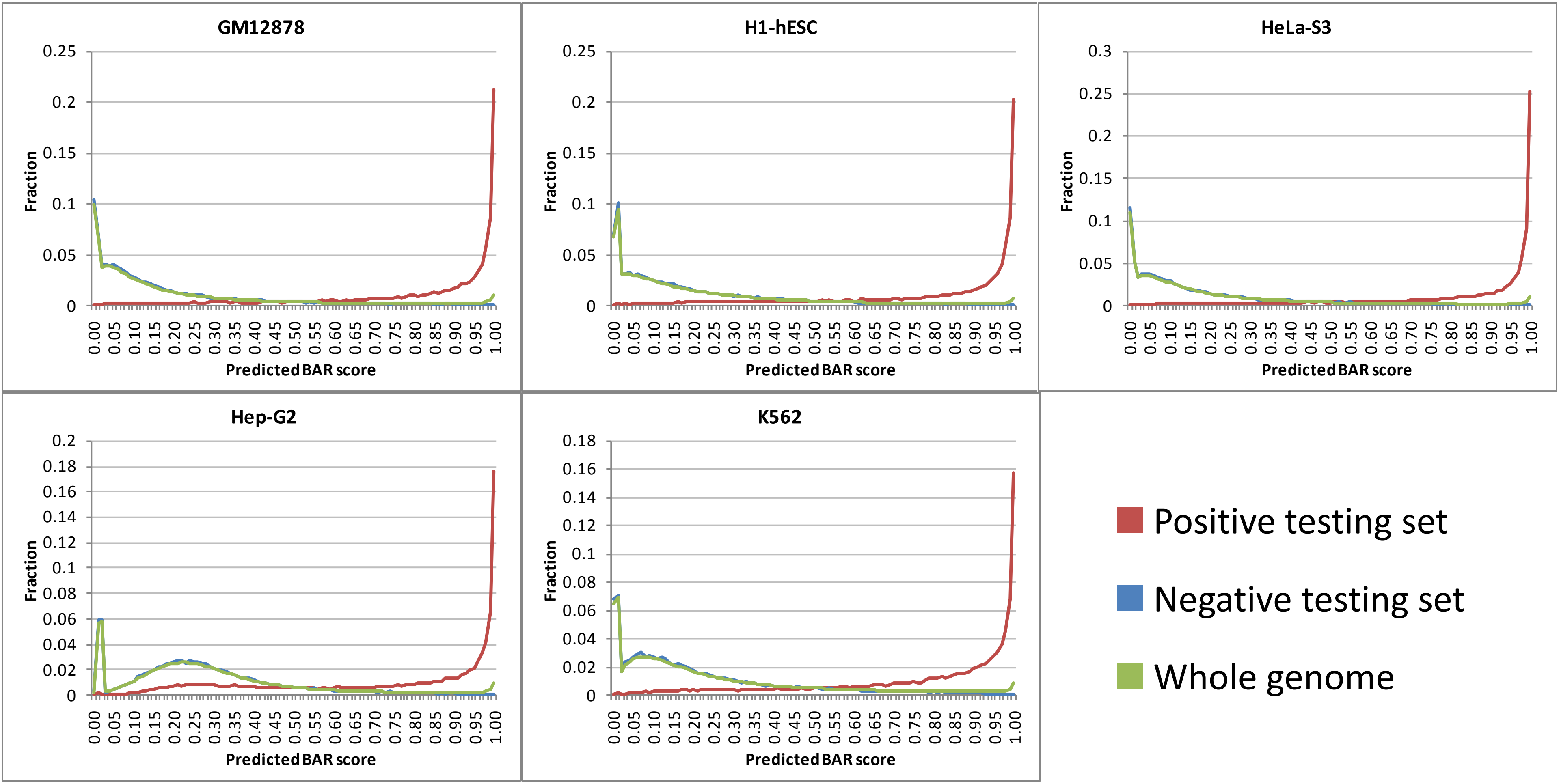
GM12878
H1-hESC
HeLa-S3
Hep-G2
K562
Fraction
Predicted BAR score
Positive testing set
Negative testing set
Whole genome

Figure S3

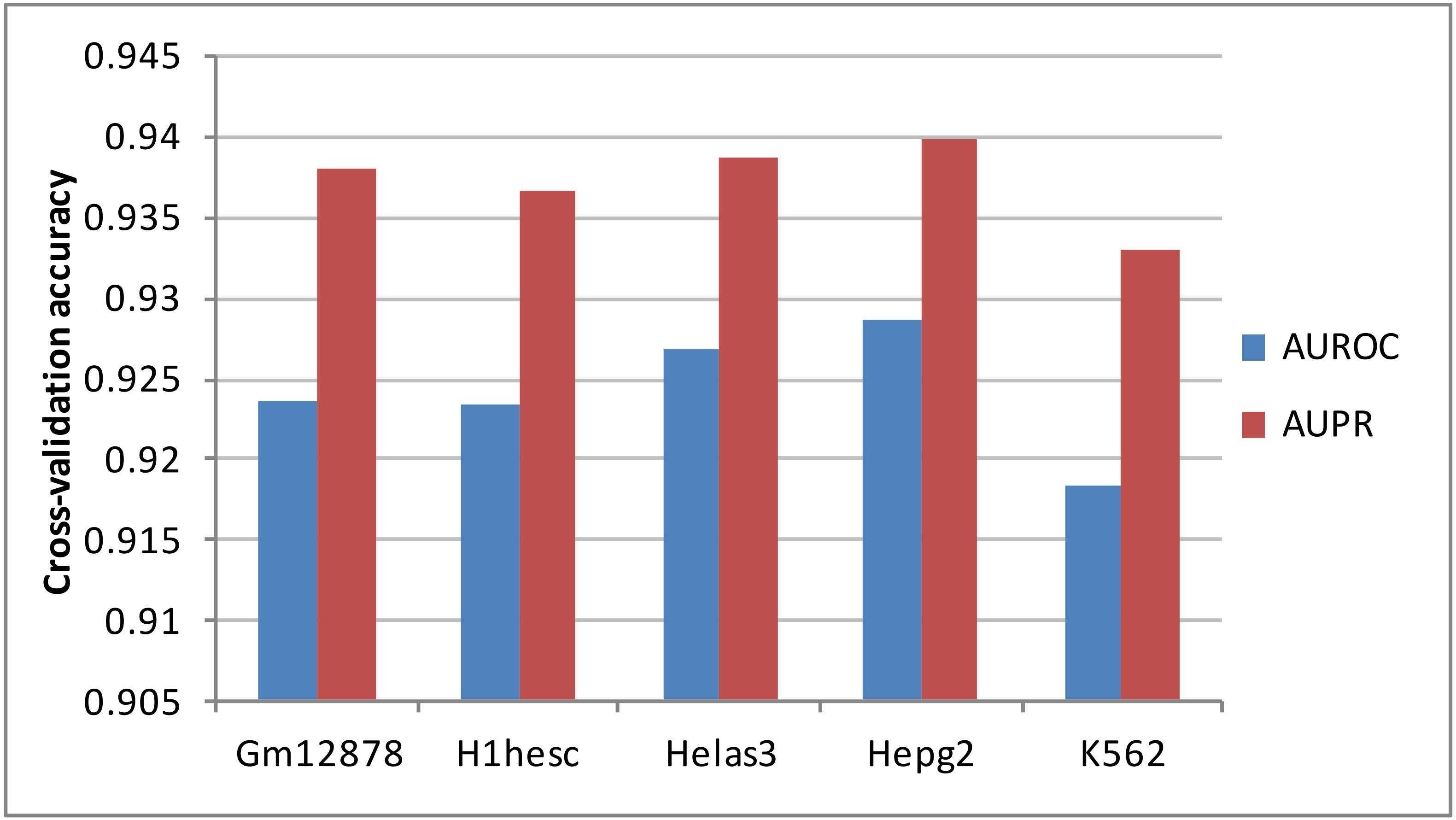
0.945
0.94
0.935
0.93
0.925
0.92
0.915
0.91
0.905
Cross-validation accuracy
Gm12878
H1hesc
Helas3
Hepg2
K562
AUROC
AUPR

Figure S4

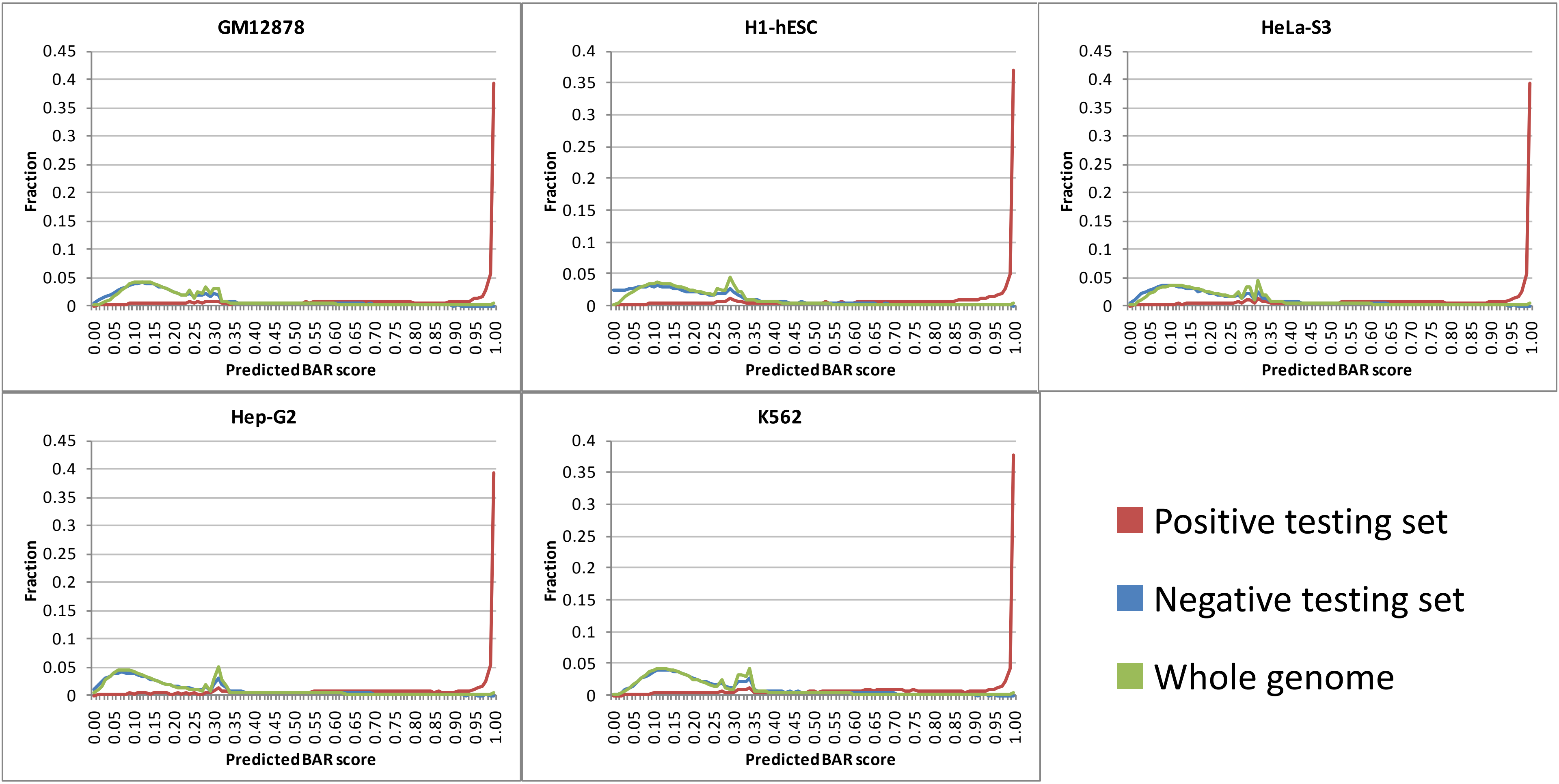
GM12878
H1-hESC
HeLa-S3
Hep-G2
K562
Fraction
Predicted BAR score
0.00 0.05 0.10 0.15 0.20 0.25 0.30 0.35 0.40 0.45 0.50 0.55 0.60 0.65 0.70 0.75 0.80 0.85 0.90 0.95 1.00
0 0.05 0.1 0.15 0.2 0.25 0.3 0.35 0.4 0.45
Positive testing set
Negative testing set
Whole genome

Figure S5

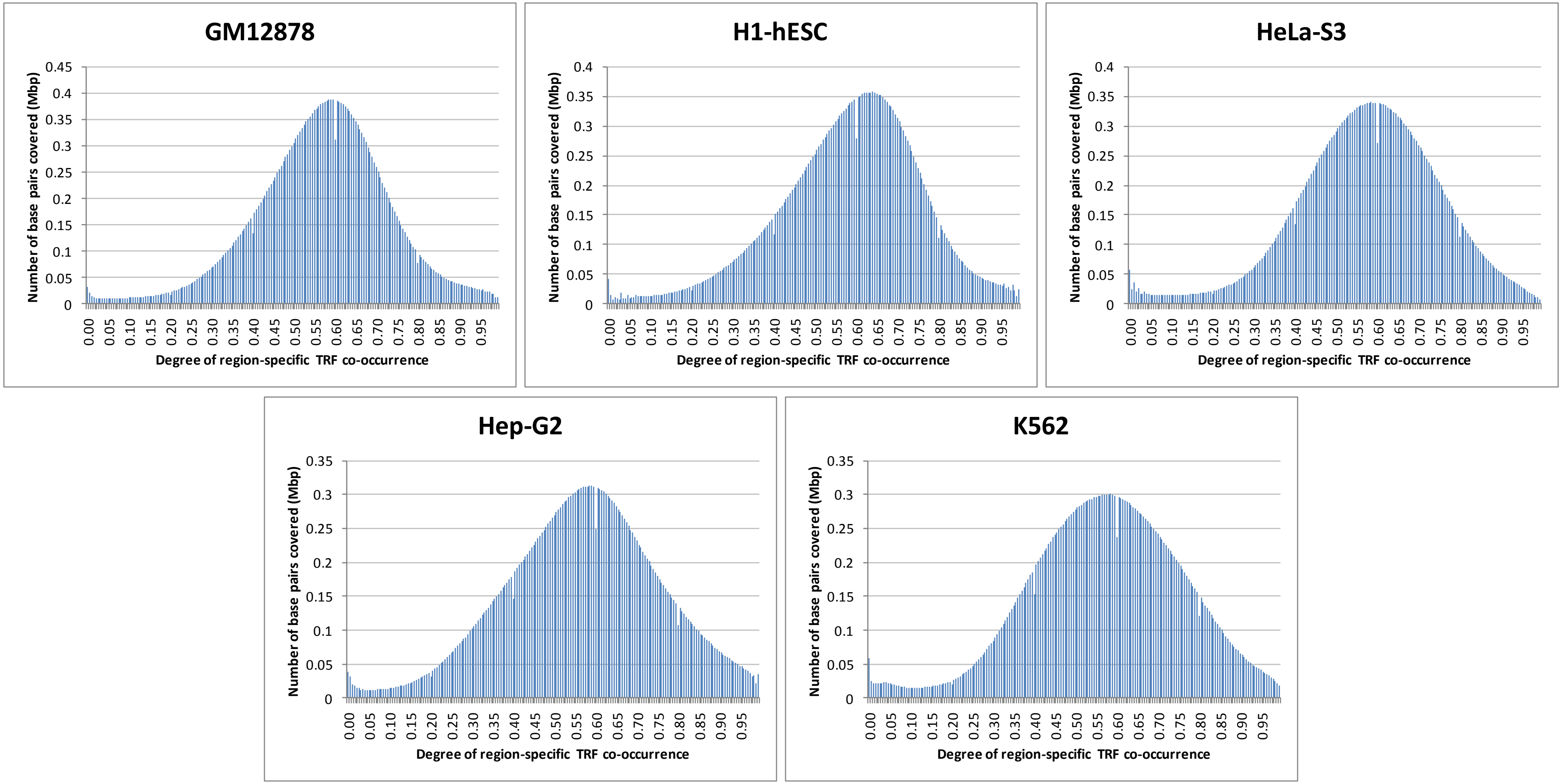
GM12878
H1-hESC
HeLa-S3
Hep-G2
K562
Number of base pairs covered (Mbp)
Degree of region-specific TRF co-occurrence
0.00 0.05 0.10 0.15 0.20 0.25 0.30 0.35 0.40 0.45 0.50 0.55 0.60 0.65 0.70 0.75 0.80 0.85 0.90 0.95
0 0.05 0.1 0.15 0.2 0.25 0.3 0.35 0.4 0.45

Figure S6A

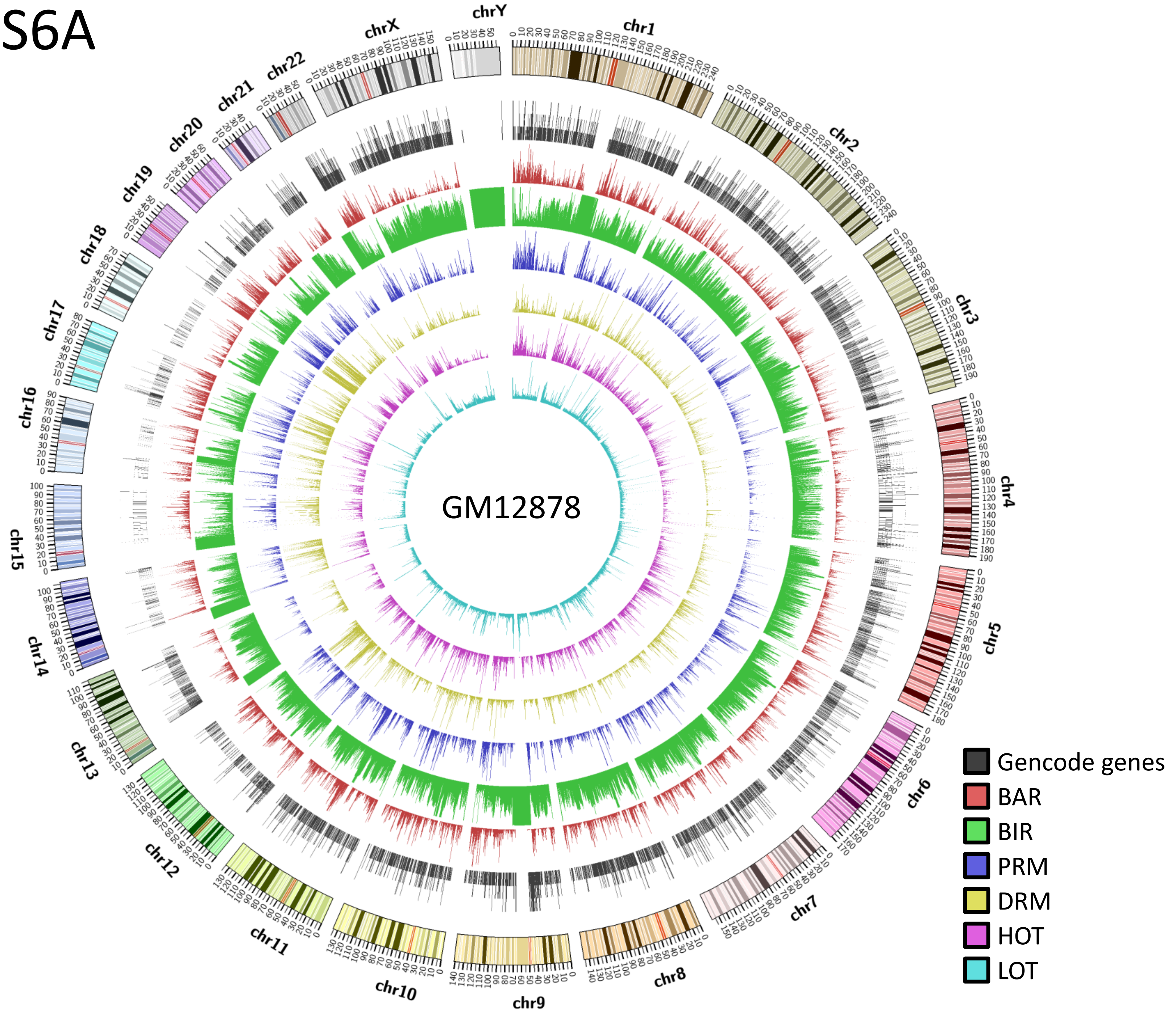
GM12878
chr1
chr2
chr3
chr4
chr5
chr6
chr7
chr8
chr9
chr10
chr11
chr12
chr13
chr14
chr15
chr16
chr17
chr18
chr19
chr20
chr21
chr22
chrX
chrY
Gencode genes
BAR
BIR
PRM
DRM
HOT
LOT

Figure S6B

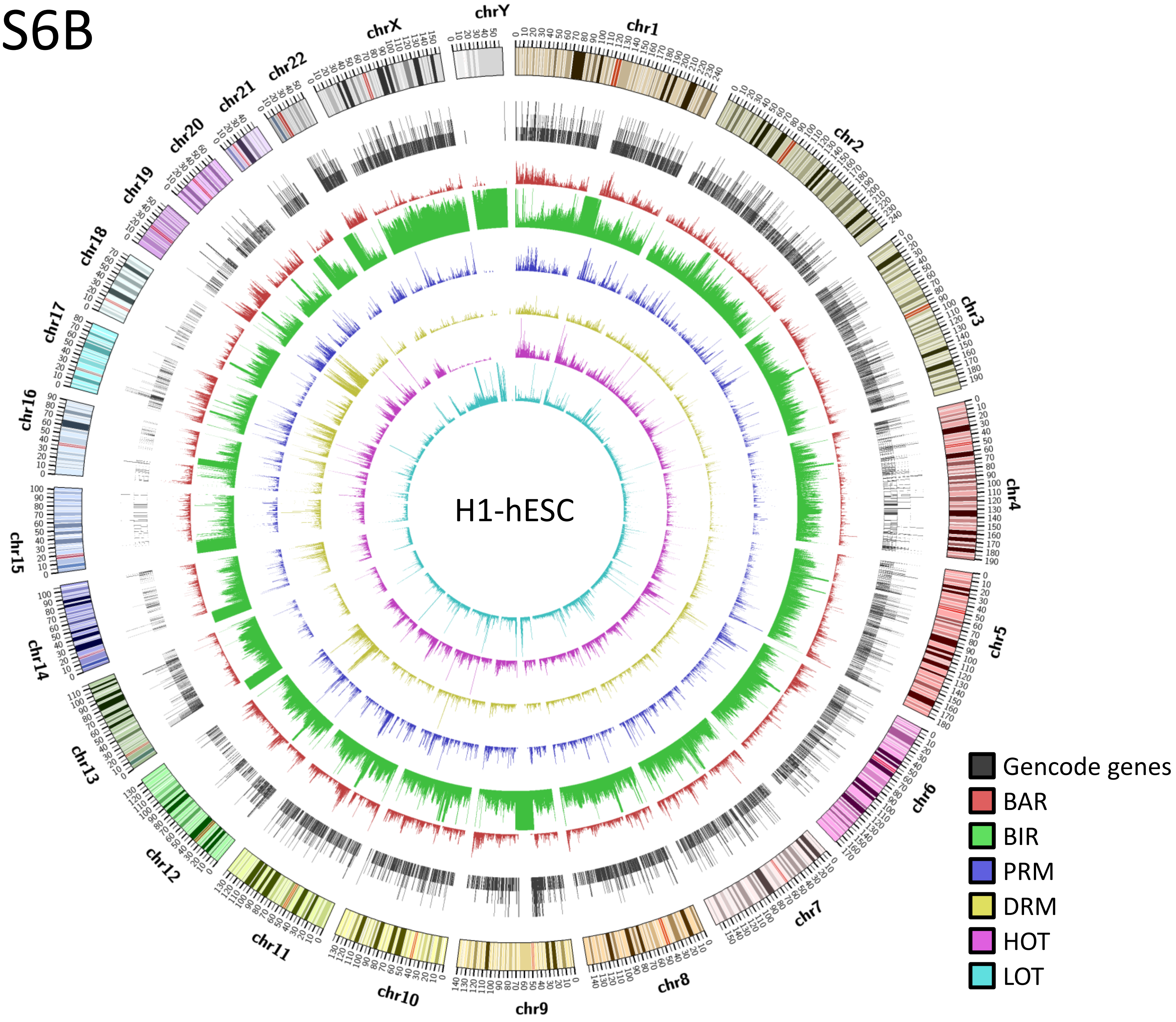
H1-hESC
chr1
chr2
chr3
chr4
chr5
chr6
chr7
chr8
chr9
chr10
chr11
chr12
chr13
chr14
chr15
chr16
chr17
chr18
chr19
chr20
chr21
chr22
chrX
chrY
Gencode genes
BAR
BIR
PRM
DRM
HOT
LOT

Figure S6C

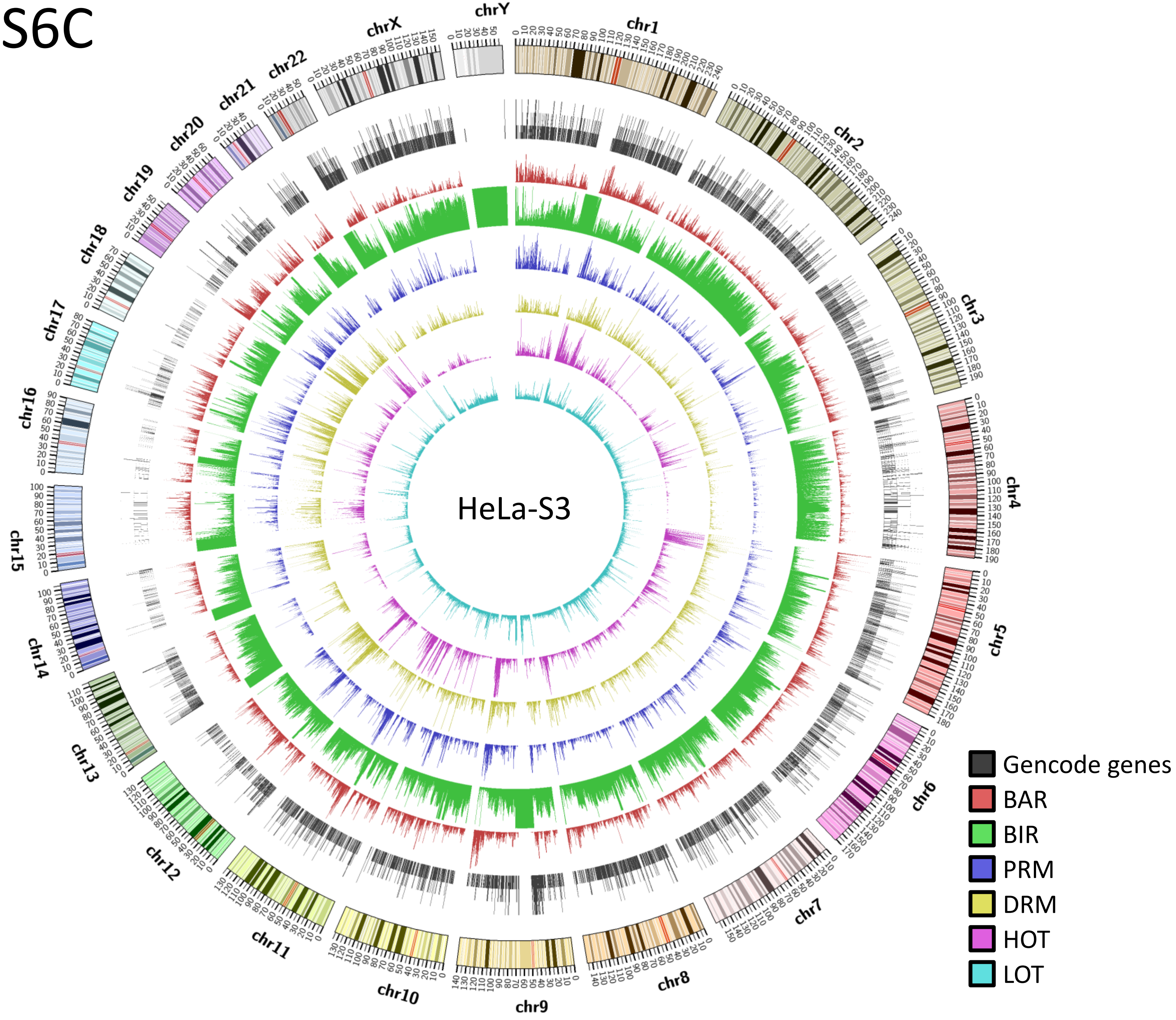
HeLa-S3
chr1
chr2
chr3
chr4
chr5
chr6
chr7
chr8
chr9
chr10
chr11
chr12
chr13
chr14
chr15
chr16
chr17
chr18
chr19
chr20
chr21
chr22
chrX
chrY
Gencode genes
BAR
BIR
PRM
DRM
HOT
LOT

Figure S6D

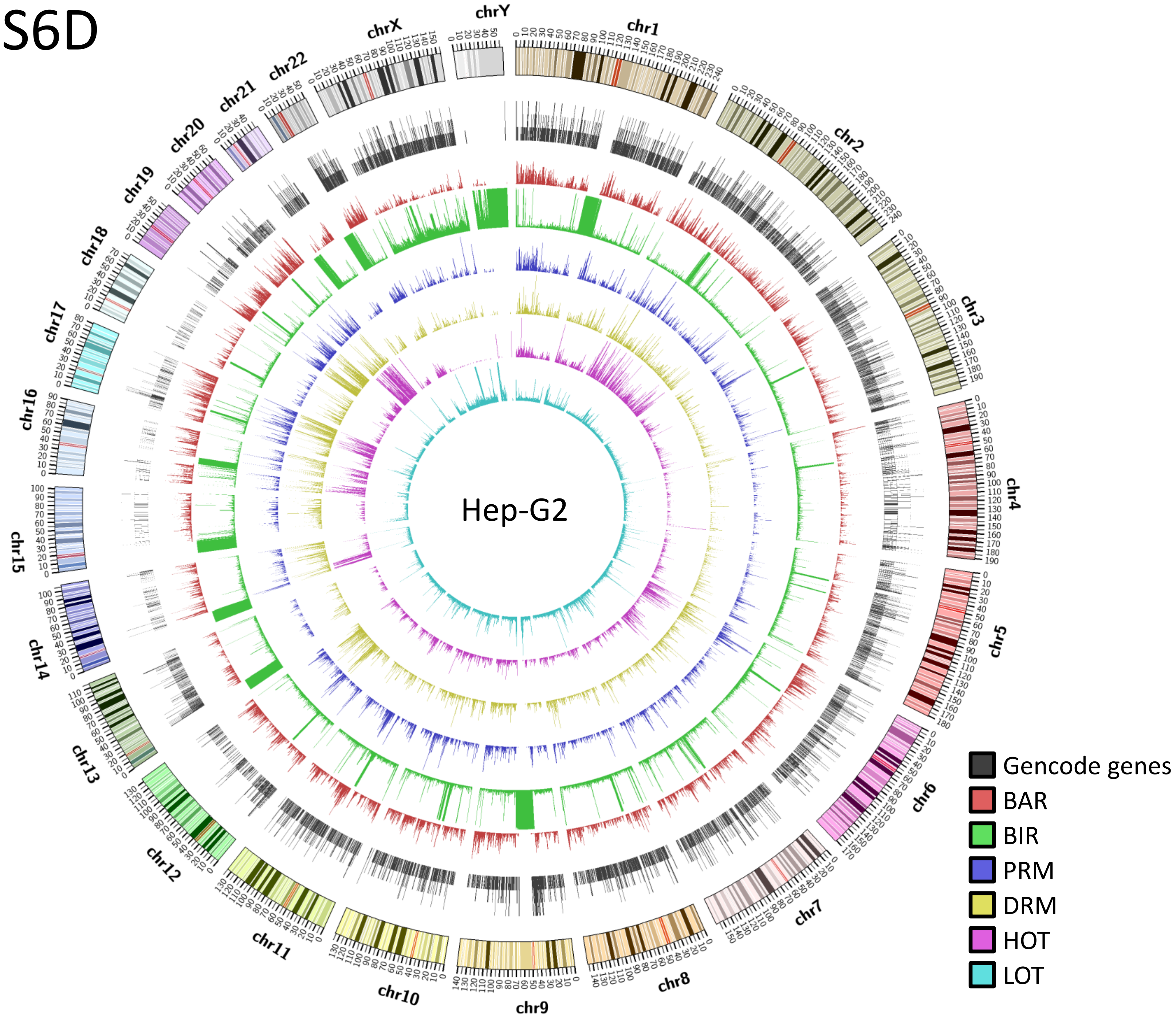
Hep-G2
chr1
chr2
chr3
chr4
chr5
chr6
chr7
chr8
chr9
chr10
chr11
chr12
chr13
chr14
chr15
chr16
chr17
chr18
chr19
chr20
chr21
chr22
chrX
chrY
Gencode genes
BAR
BIR
PRM
DRM
HOT
LOT

Figure S6E

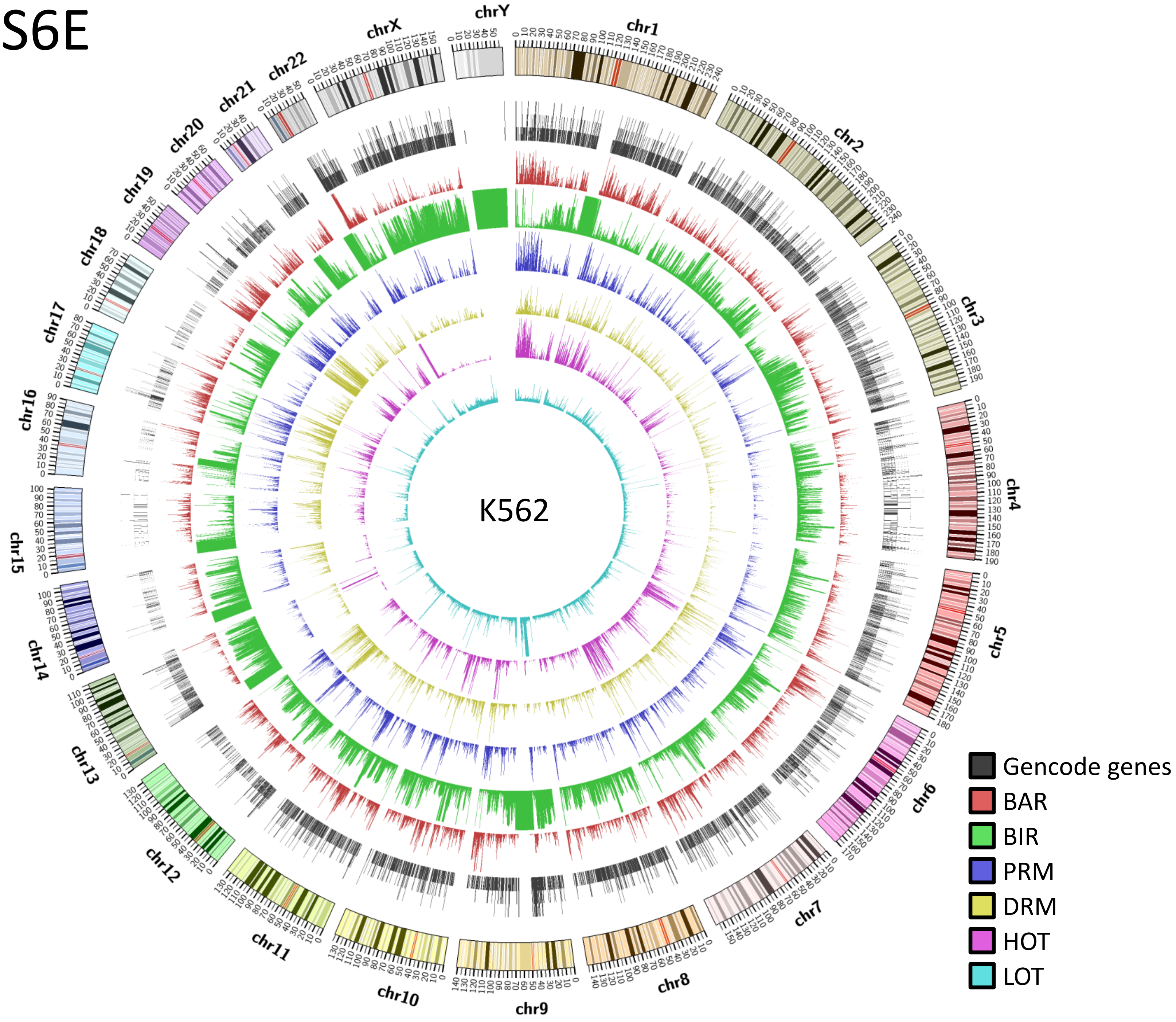
K562
chr1
chr2
chr3
chr4
chr5
chr6
chr7
chr8
chr9
chr10
chr11
chr12
chr13
chr14
chr15
chr16
chr17
chr18
chr19
chr20
chr21
chr22
chrX
chrY
Gencode genes
BAR
BIR
PRM
DRM
HOT
LOT

Figure S7A

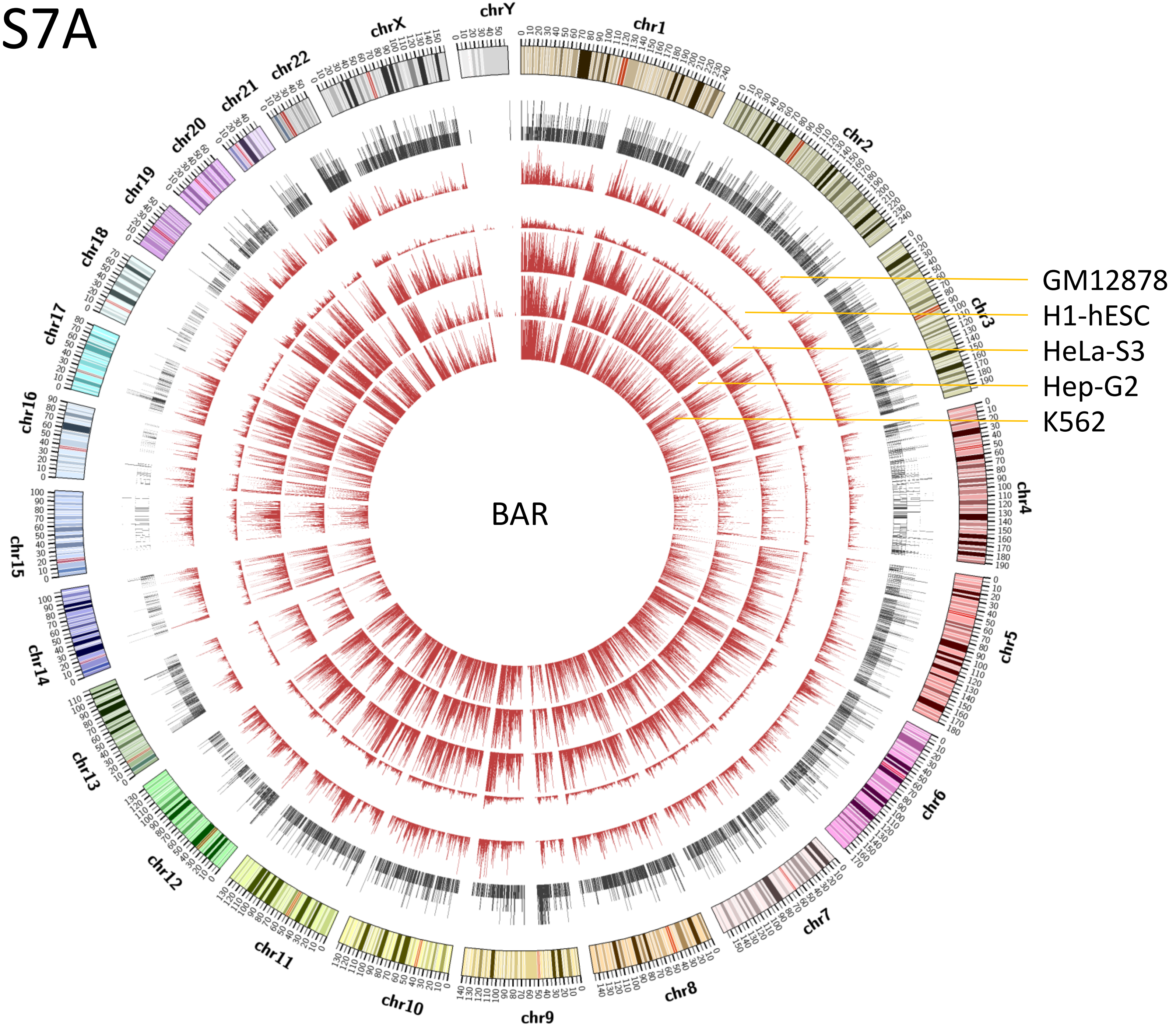

chr1
chr2
chr3
chr4
chr5
chr6
chr7
chr8
chr9
chr10
chr11
chr12
chr13
chr14
chr15
chr16
chr17
chr18
chr19
chr20
chr21
chr22
chrX
chrY
GM12878
H1-hESC
HeLa-S3
Hep-G2
K562
BAR

Figure S7B

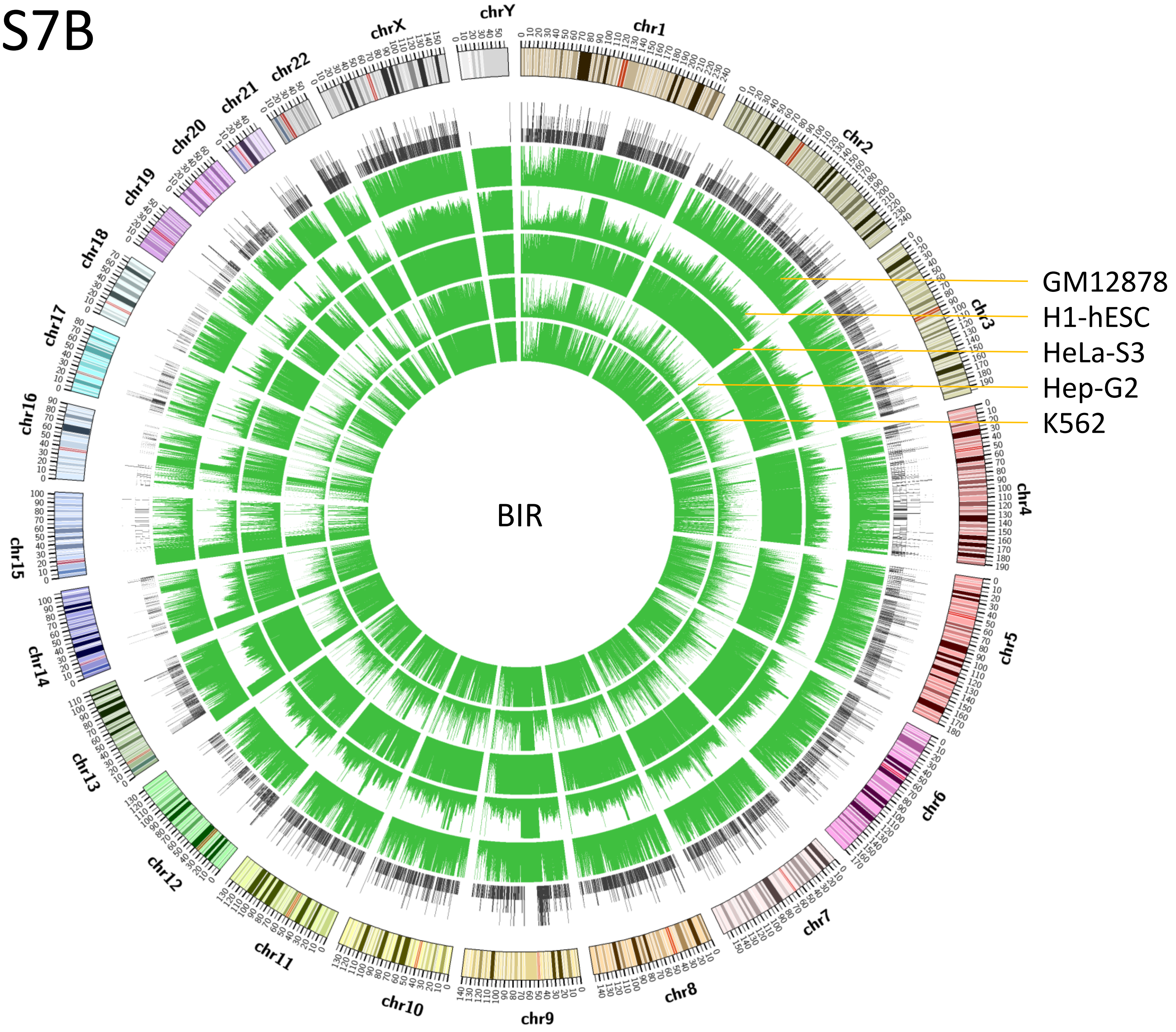
BIR
GM12878
H1-hESC
HeLa-S3
Hep-G2
K562
chr1
chr2
chr3
chr4
chr5
chr6
chr7
chr8
chr9
chr10
chr11
chr12
chr13
chr14
chr15
chr16
chr17
chr18
chr19
chr20
chr21
chr22
chrX
chrY

Figure S7C

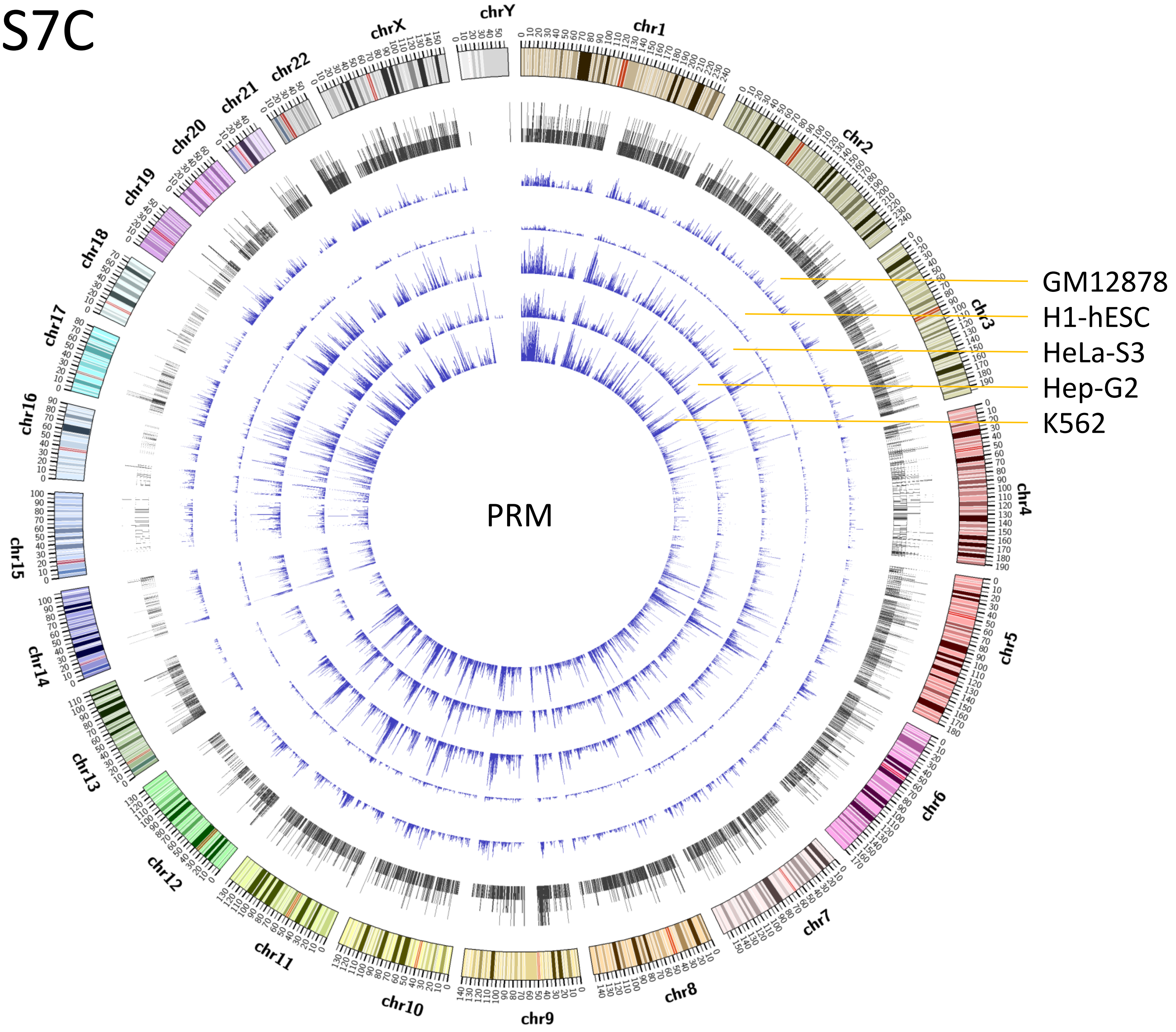
PRM
GM12878
H1-hESC
HeLa-S3
Hep-G2
K562
chr1
chr2
chr3
chr4
chr5
chr6
chr7
chr8
chr9
chr10
chr11
chr12
chr13
chr14
chr15
chr16
chr17
chr18
chr19
chr20
chr21
chr22
chrX
chrY

Figure S7D

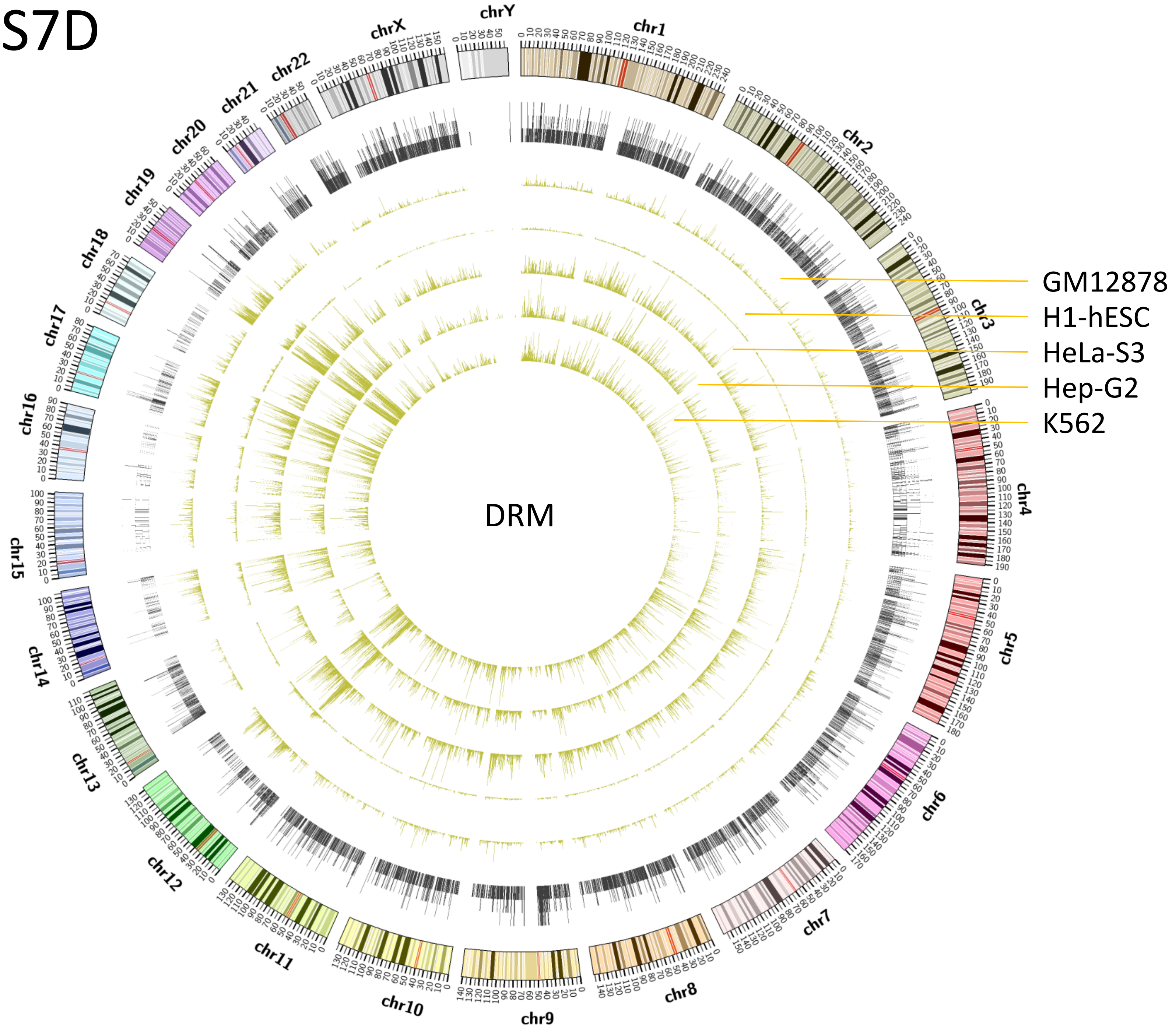
DRM
GM12878
H1-hESC
HeLa-S3
Hep-G2
K562
chr1
chr2
chr3
chr4
chr5
chr6
chr7
chr8
chr9
chr10
chr11
chr12
chr13
chr14
chr15
chr16
chr17
chr18
chr19
chr20
chr21
chr22
chrX
chrY

Figure S7E

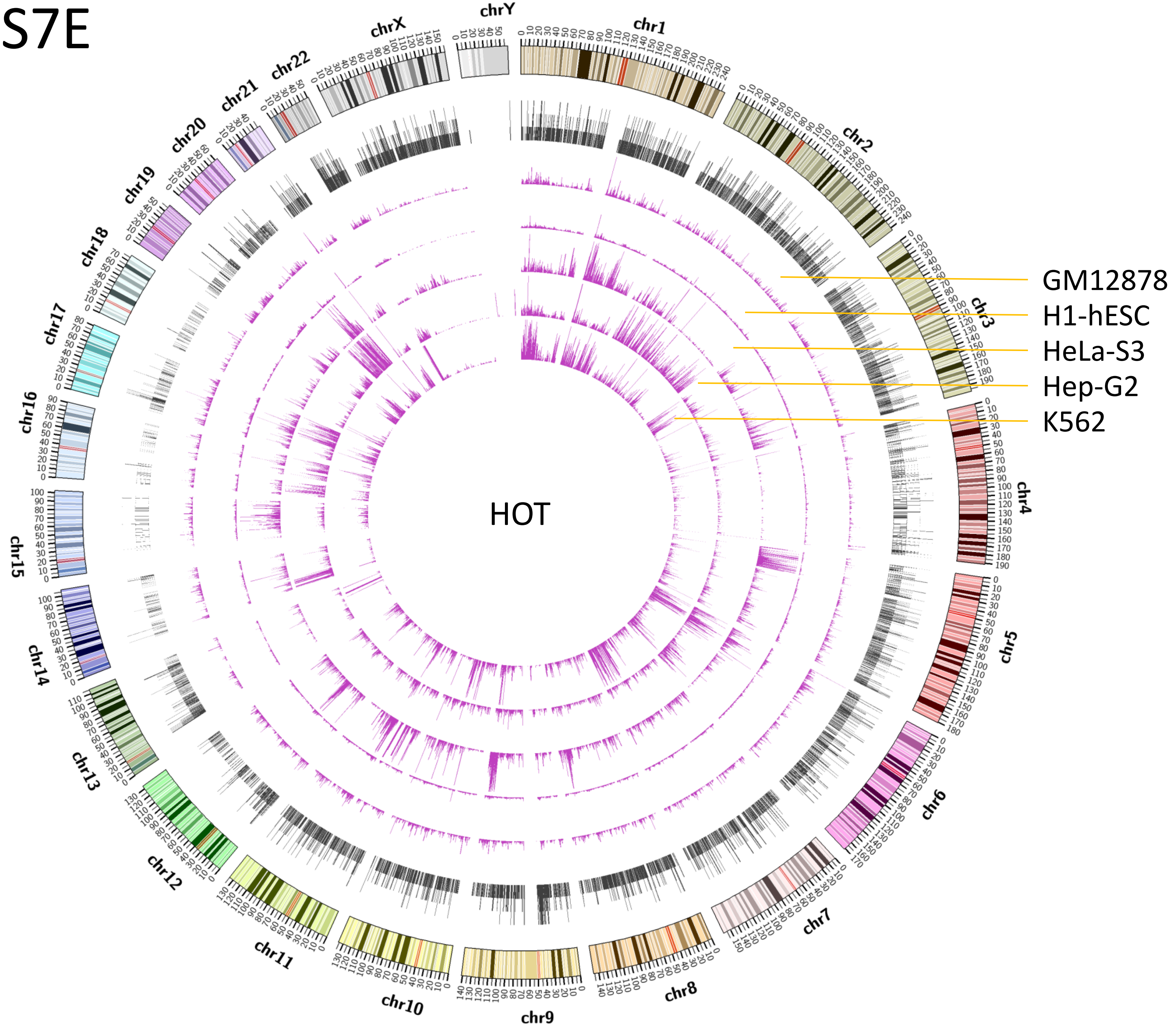
chrY
chr1
chr2
chr3
chr4
chr5
chr6
chr7
chr8
chr9
chr10
chr11
chr12
chr13
chr14
chr15
chr16
chr17
chr18
chr19
chr20
chr21
chr22
chrX
GM12878
H1-hESC
HeLa-S3
Hep-G2
K562
HOT

Figure S7F

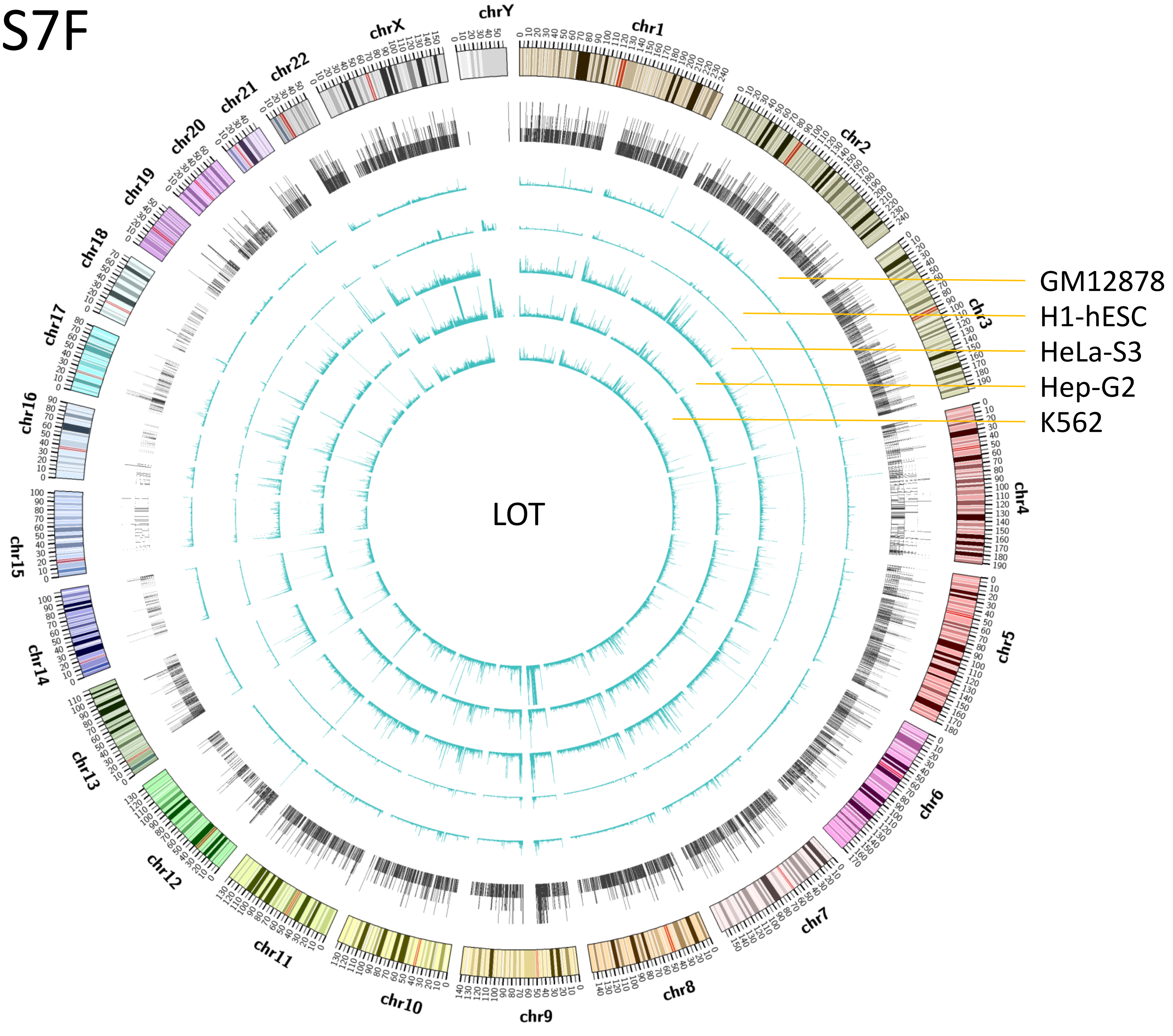
LOT
GM12878
H1-hESC
HeLa-S3
Hep-G2
K562
chr1
chr2
chr3
chr4
chr5
chr6
chr7
chr8
chr9
chr10
chr11
chr12
chr13
chr14
chr15
chr16
chr17
chr18
chr19
chr20
chr21
chr22
chrX
chrY

Figure S8A

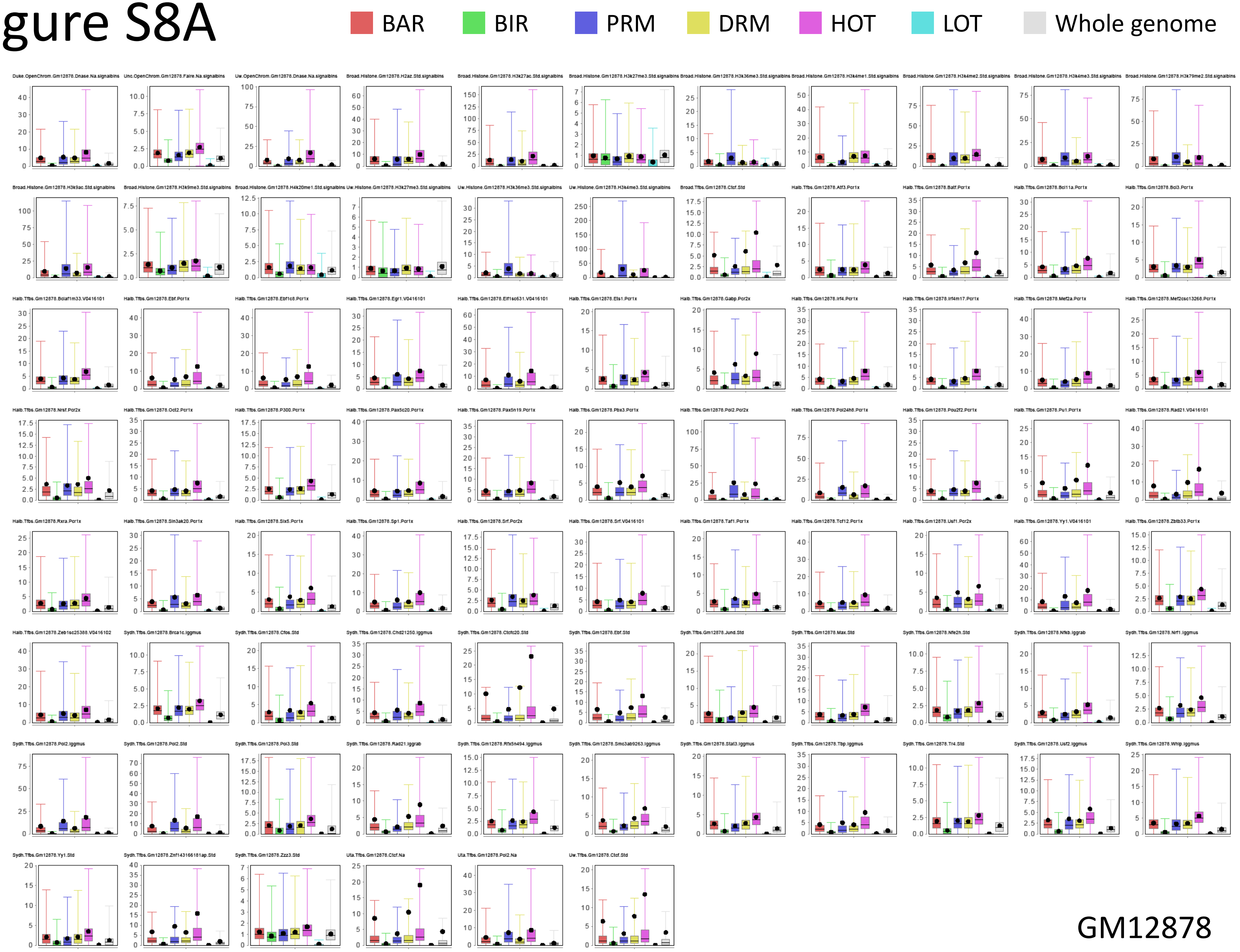
BAR
BIR
PRM
DRM
HOT
LOT
Whole genome
Duke.OpenChrom.Gm12878.Dnase.Na.signalbins
Unc.OpenChrom.Gm12878.Faire.Na.signalbins
Uw.OpenChrom.Gm12878.Dnase.Na.signalbins
Broad.Histone.Gm12878.H2az.Std.signalbins
Broad.Histone.Gm12878.H3k27ac.Std.signalbins
Broad.Histone.Gm12878.H3k27me3.Std.signalbins
Broad.Histone.Gm12878.H3k36me3.Std.signalbins
Broad.Histone.Gm12878.H3k4me1.Std.signalbins
Broad.Histone.Gm12878.H3k4me2.Std.signalbins
Broad.Histone.Gm12878.H3k4me3.Std.signalbins
Broad.Histone.Gm12878.H3k79me2.Std.signalbins
Broad.Histone.Gm12878.H3k9ac.Std.signalbins
Broad.Histone.Gm12878.H3k9me3.Std.signalbins
Broad.Histone.Gm12878.H4k20me1.Std.signalbins
Uw.Histone.Gm12878.H3k27me3.Std.signalbins
Uw.Histone.Gm12878.H3k36me3.Std.signalbins
Uw.Histone.Gm12878.H3k4me3.Std.signalbins
Broad.Tfbs.Gm12878.Ctcf.Std
Haib.Tfbs.Gm12878.Atf3.Pcr1x
Haib.Tfbs.Gm12878.Batf.Pcr1x
Haib.Tfbs.Gm12878.Bcl11a.Pcr1x
Haib.Tfbs.Gm12878.Bcl3.Pcr1x
Haib.Tfbs.Gm12878.Bclaf1m33.V0416101
Haib.Tfbs.Gm12878.Ebf.Pcr1x
Haib.Tfbs.Gm12878.Ebf1c8.Pcr1x
Haib.Tfbs.Gm12878.Egr1.V0416101
Haib.Tfbs.Gm12878.Elf1sc631.V0416101
Haib.Tfbs.Gm12878.Ets1.Pcr1x
Haib.Tfbs.Gm12878.Gabp.Pcr2x
Haib.Tfbs.Gm12878.Irf4.Pcr1x
Haib.Tfbs.Gm12878.Irf4m17.Pcr1x
Haib.Tfbs.Gm12878.Mef2a.Pcr1x
Haib.Tfbs.Gm12878.Mef2csc13268.Pcr1x
Haib.Tfbs.Gm12878.Nrsf.Pcr2x
Haib.Tfbs.Gm12878.Oct2.Pcr1x
Haib.Tfbs.Gm12878.P300.Pcr1x
Haib.Tfbs.Gm12878.Pax5c20.Pcr1x
Haib.Tfbs.Gm12878.Pax5n19.Pcr1x
Haib.Tfbs.Gm12878.Pbx3.Pcr1x
Haib.Tfbs.Gm12878.Pol2.Pcr2x
Haib.Tfbs.Gm12878.Pol24h8.Pcr1x
Haib.Tfbs.Gm12878.Pou2f2.Pcr1x
Haib.Tfbs.Gm12878.Pu1.Pcr1x
Haib.Tfbs.Gm12878.Rad21.V0416101
Haib.Tfbs.Gm12878.Rxra.Pcr1x
Haib.Tfbs.Gm12878.Sin3ak20.Pcr1x
Haib.Tfbs.Gm12878.Six5.Pcr1x
Haib.Tfbs.Gm12878.Sp1.Pcr1x
Haib.Tfbs.Gm12878.Srf.Pcr2x
Haib.Tfbs.Gm12878.Srf.V0416101
Haib.Tfbs.Gm12878.Taf1.Pcr1x
Haib.Tfbs.Gm12878.Tcf12.Pcr1x
Haib.Tfbs.Gm12878.Usf1.Pcr2x
Haib.Tfbs.Gm12878.Yy1.V0416101
Haib.Tfbs.Gm12878.Zbtb33.Pcr1x
Haib.Tfbs.Gm12878.Zeb1sc25388.V0416102
Sydh.Tfbs.Gm12878.Brca1c.Iggmus
Sydh.Tfbs.Gm12878.Cfos.Std
Sydh.Tfbs.Gm12878.Chd21250.Iggmus
Sydh.Tfbs.Gm12878.Ctcfc20.Std
Sydh.Tfbs.Gm12878.Ebf.Std
Sydh.Tfbs.Gm12878.Jund.Std
Sydh.Tfbs.Gm12878.Max.Std
Sydh.Tfbs.Gm12878.Nfe2h.Std
Sydh.Tfbs.Gm12878.Nfkb.Iggrab
Sydh.Tfbs.Gm12878.Nrf1.Iggmus
Sydh.Tfbs.Gm12878.Pol2.Iggmus
Sydh.Tfbs.Gm12878.Pol2.Std
Sydh.Tfbs.Gm12878.Pol3.Std
Sydh.Tfbs.Gm12878.Rad21.Iggrab
Sydh.Tfbs.Gm12878.Rfx5n494.Iggmus
Sydh.Tfbs.Gm12878.Smc3ab9263.Iggmus
Sydh.Tfbs.Gm12878.Stat3.Iggmus
Sydh.Tfbs.Gm12878.Tbp.Iggmus
Sydh.Tfbs.Gm12878.Tr4.Std
Sydh.Tfbs.Gm12878.Usf2.Iggmus
Sydh.Tfbs.Gm12878.Whip.Iggmus
Sydh.Tfbs.Gm12878.Yy1.Std
Sydh.Tfbs.Gm12878.Znf143166181ap.Std
Sydh.Tfbs.Gm12878.Zzz3.Std
Uta.Tfbs.Gm12878.Ctcf.Na
Uta.Tfbs.Gm12878.Pol2.Na
Uw.Tfbs.Gm12878.Ctcf.Std
GM12878

Figure S8B

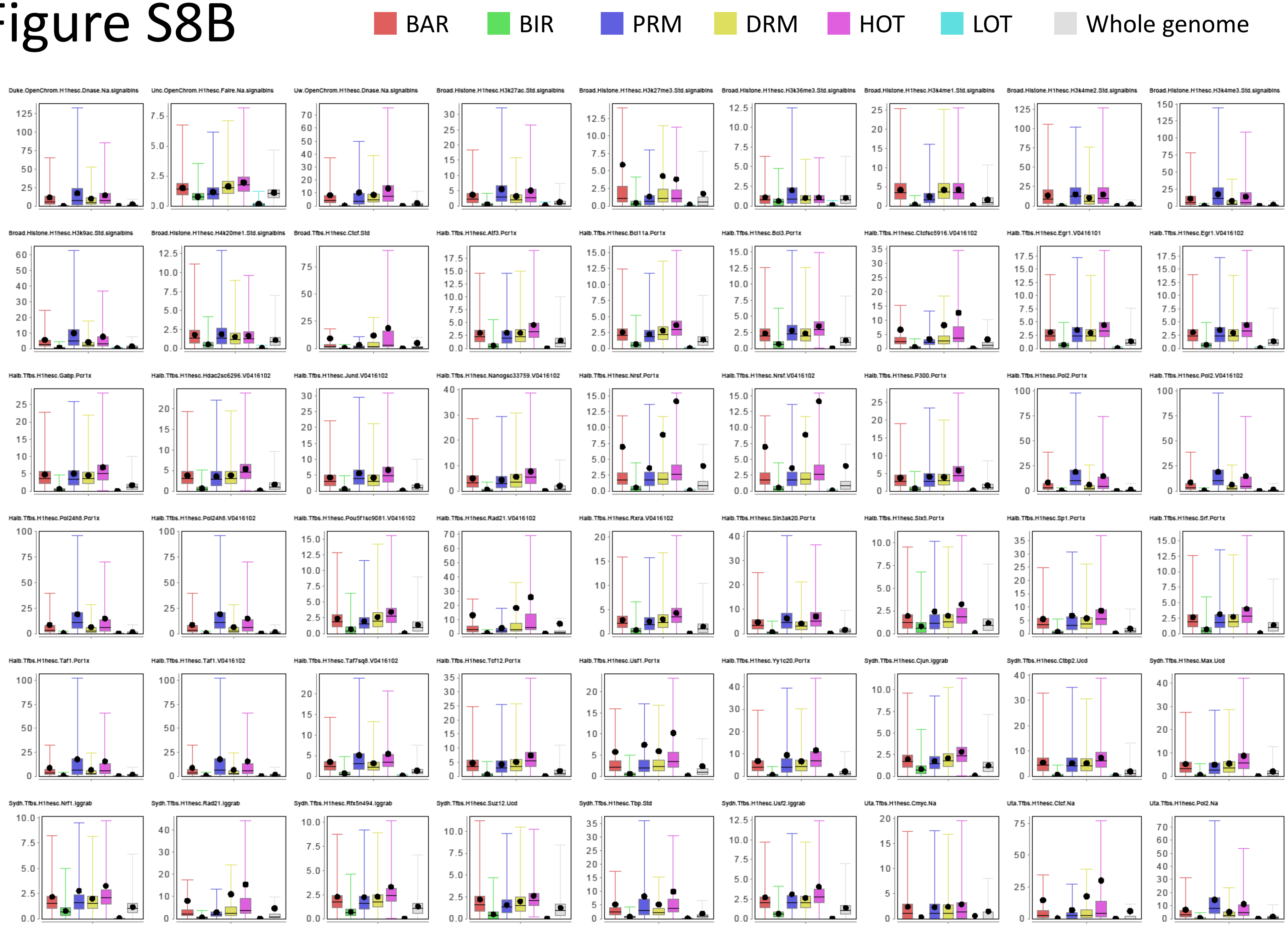

H1-hESC

Figure S8C

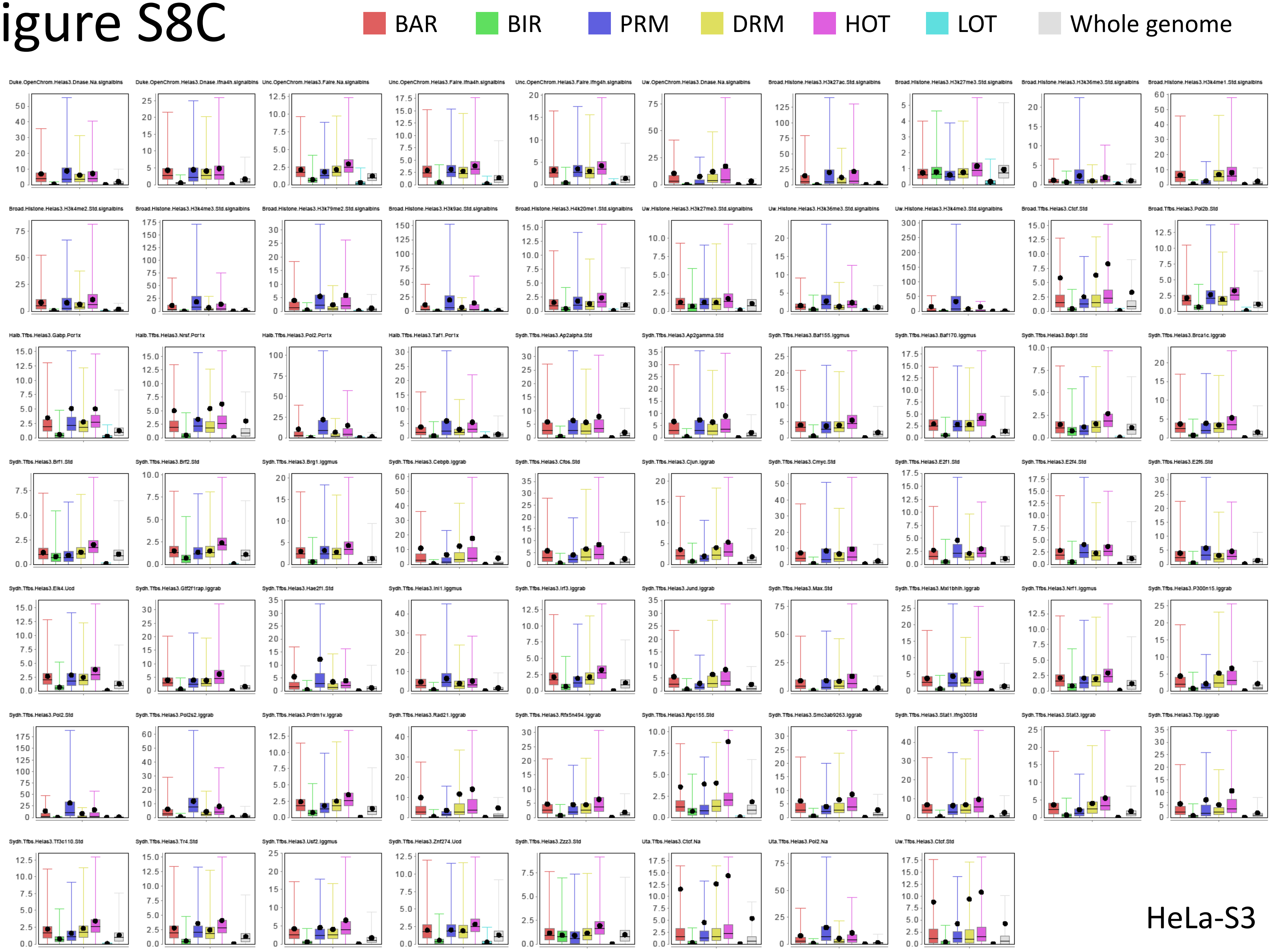
BAR
BIR
PRM
DRM
HOT
LOT
Whole genome
Duke.OpenChrom.Helas3.Dnase.Na.signalbins
Duke.OpenChrom.Helas3.Dnase.Ifna4h.signalbins
Unc.OpenChrom.Helas3.Faire.Na.signalbins
Unc.OpenChrom.Helas3.Faire.Ifna4h.signalbins
Unc.OpenChrom.Helas3.Faire.Ifng4h.signalbins
Uw.OpenChrom.Helas3.Dnase.Na.signalbins
Broad.Histone.Helas3.H3k27ac.Std.signalbins
Broad.Histone.Helas3.H3k27me3.Std.signalbins
Broad.Histone.Helas3.H3k36me3.Std.signalbins
Broad.Histone.Helas3.H3k4me1.Std.signalbins
Broad.Histone.Helas3.H3k4me2.Std.signalbins
Broad.Histone.Helas3.H3k4me3.Std.signalbins
Broad.Histone.Helas3.H3k79me2.Std.signalbins
Broad.Histone.Helas3.H3k9ac.Std.signalbins
Broad.Histone.Helas3.H4k20me1.Std.signalbins
Uw.Histone.Helas3.H3k27me3.Std.signalbins
Uw.Histone.Helas3.H3k36me3.Std.signalbins
Uw.Histone.Helas3.H3k4me3.Std.signalbins
Broad.Tfbs.Helas3.Ctcf.Std
Broad.Tfbs.Helas3.Pol2b.Std
Haib.Tfbs.Helas3.Gabp.Pcr1x
Haib.Tfbs.Helas3.Nrsf.Pcr1x
Haib.Tfbs.Helas3.Pol2.Pcr1x
Haib.Tfbs.Helas3.Taf1.Pcr1x
Sydh.Tfbs.Helas3.Ap2alpha.Std
Sydh.Tfbs.Helas3.Ap2gamma.Std
Sydh.Tfbs.Helas3.Baf155.Iggmus
Sydh.Tfbs.Helas3.Baf170.Iggmus
Sydh.Tfbs.Helas3.Bdp1.Std
Sydh.Tfbs.Helas3.Brca1c.Iggrab
Sydh.Tfbs.Helas3.Brf1.Std
Sydh.Tfbs.Helas3.Brf2.Std
Sydh.Tfbs.Helas3.Brg1.Iggmus
Sydh.Tfbs.Helas3.Cebpb.Iggrab
Sydh.Tfbs.Helas3.Cfos.Std
Sydh.Tfbs.Helas3.Cjun.Iggrab
Sydh.Tfbs.Helas3.Cmyc.Std
Sydh.Tfbs.Helas3.E2f1.Std
Sydh.Tfbs.Helas3.E2f4.Std
Sydh.Tfbs.Helas3.E2f6.Std
Sydh.Tfbs.Helas3.Elk4.Ucd
Sydh.Tfbs.Helas3.Gtf2f1rap.Iggrab
Sydh.Tfbs.Helas3.Hae2f1.Std
Sydh.Tfbs.Helas3.Ini1.Iggmus
Sydh.Tfbs.Helas3.Irf3.Iggrab
Sydh.Tfbs.Helas3.Jund.Iggrab
Sydh.Tfbs.Helas3.Max.Std
Sydh.Tfbs.Helas3.Mxi1bhlh.Iggrab
Sydh.Tfbs.Helas3.Nrf1.Iggmus
Sydh.Tfbs.Helas3.P300n15.Iggrab
Sydh.Tfbs.Helas3.Pol2.Std
Sydh.Tfbs.Helas3.Pol2s2.Iggrab
Sydh.Tfbs.Helas3.Prdm1v.Iggrab
Sydh.Tfbs.Helas3.Rad21.Iggrab
Sydh.Tfbs.Helas3.Rfx5n494.Iggrab
Sydh.Tfbs.Helas3.Rpc155.Std
Sydh.Tfbs.Helas3.Smc3ab9263.Iggrab
Sydh.Tfbs.Helas3.Stat1.Ifng30Std
Sydh.Tfbs.Helas3.Stat3.Iggrab
Sydh.Tfbs.Helas3.Tbp.Iggrab
Sydh.Tfbs.Helas3.Tf3c110.Std
Sydh.Tfbs.Helas3.Tr4.Std
Sydh.Tfbs.Helas3.Usf2.Iggmus
Sydh.Tfbs.Helas3.Znf274.Ucd
Sydh.Tfbs.Helas3.Zzz3.Std
Uta.Tfbs.Helas3.Ctcf.Na
Uta.Tfbs.Helas3.Pol2.Na
Uw.Tfbs.Helas3.Ctcf.Std
HeLa-S3

Figure S8D

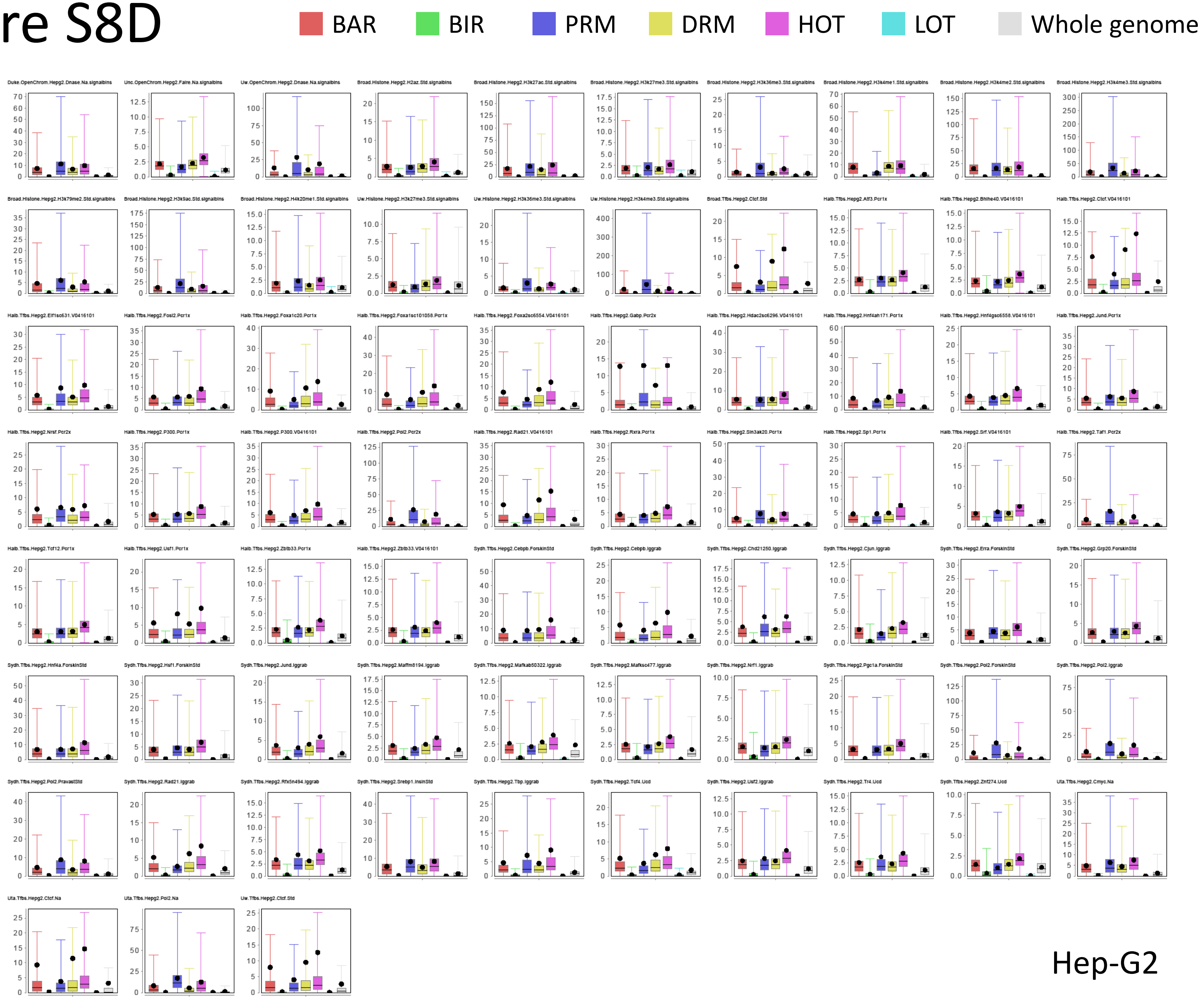

Figure S8E

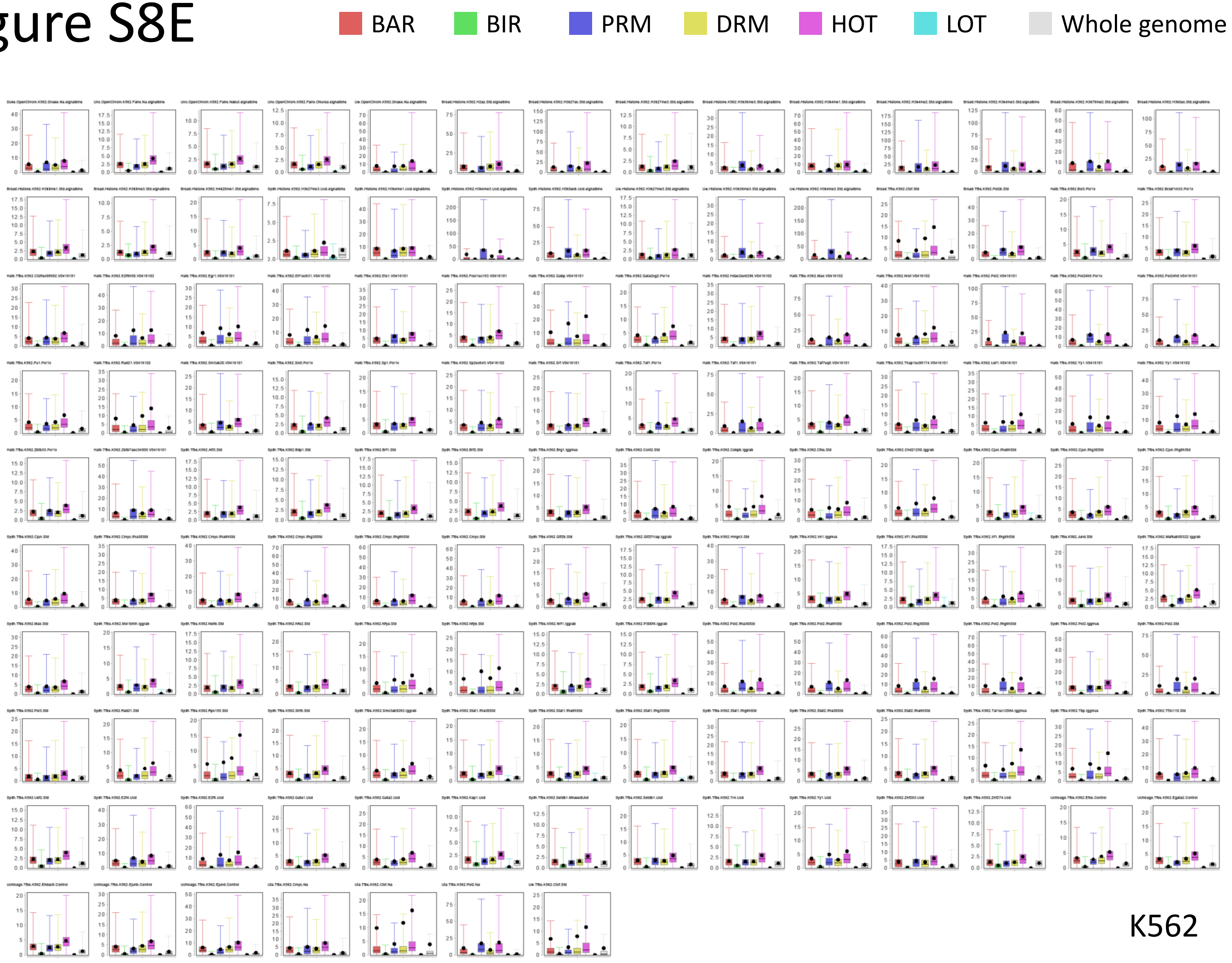

Figure S9

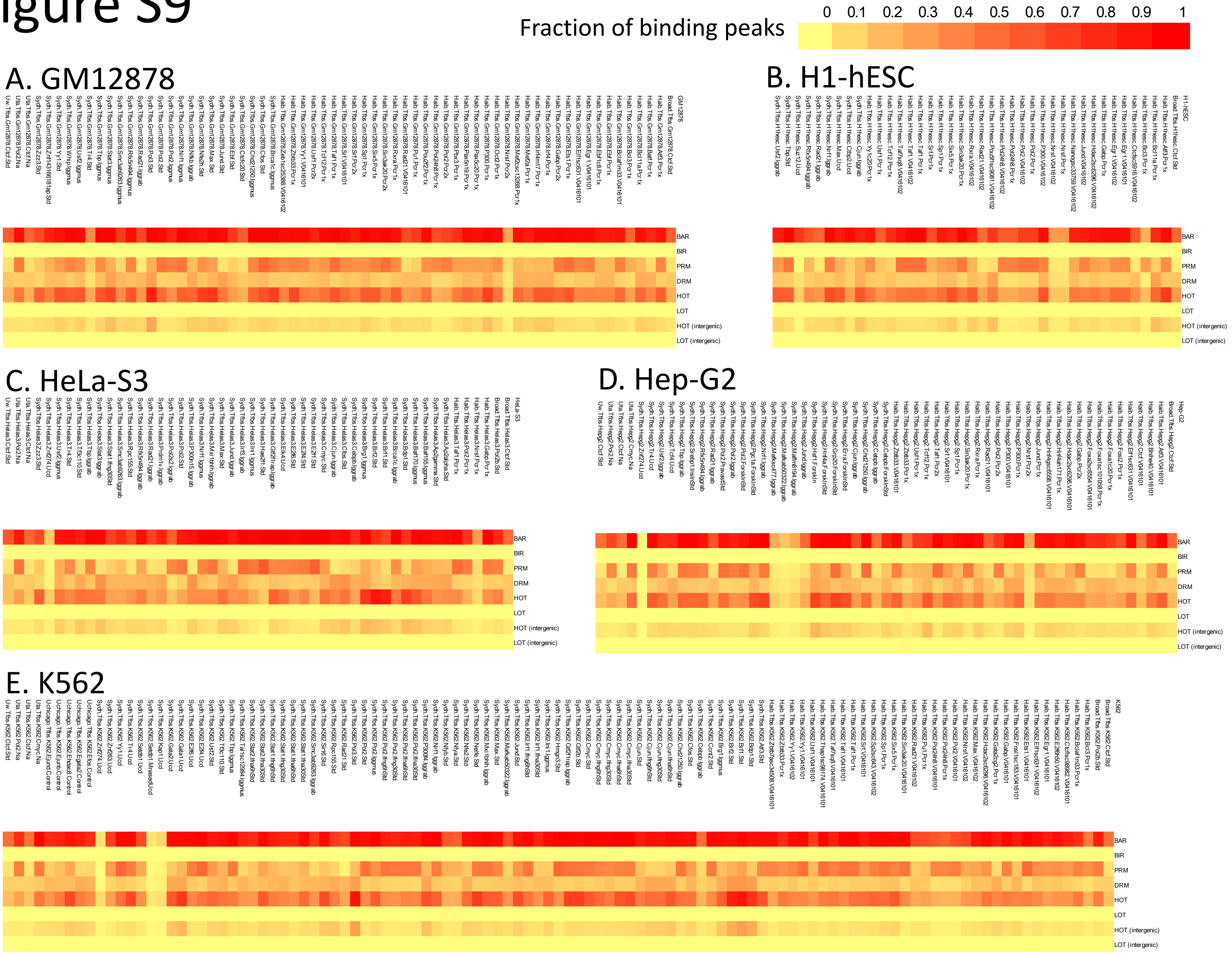

Fraction of binding peaks
0
0.1
0.2
0.3
0.4
0.5
0.6
0.7
0.8
0.9
1
A. GM12878
B. H1-hESC
C. HeLa-S3
D. Hep-G2
E. K562
BAR
BIR
PRM
DRM
HOT
LOT
HOT (intergenic)
LOT (intergenic)

Figure S10A

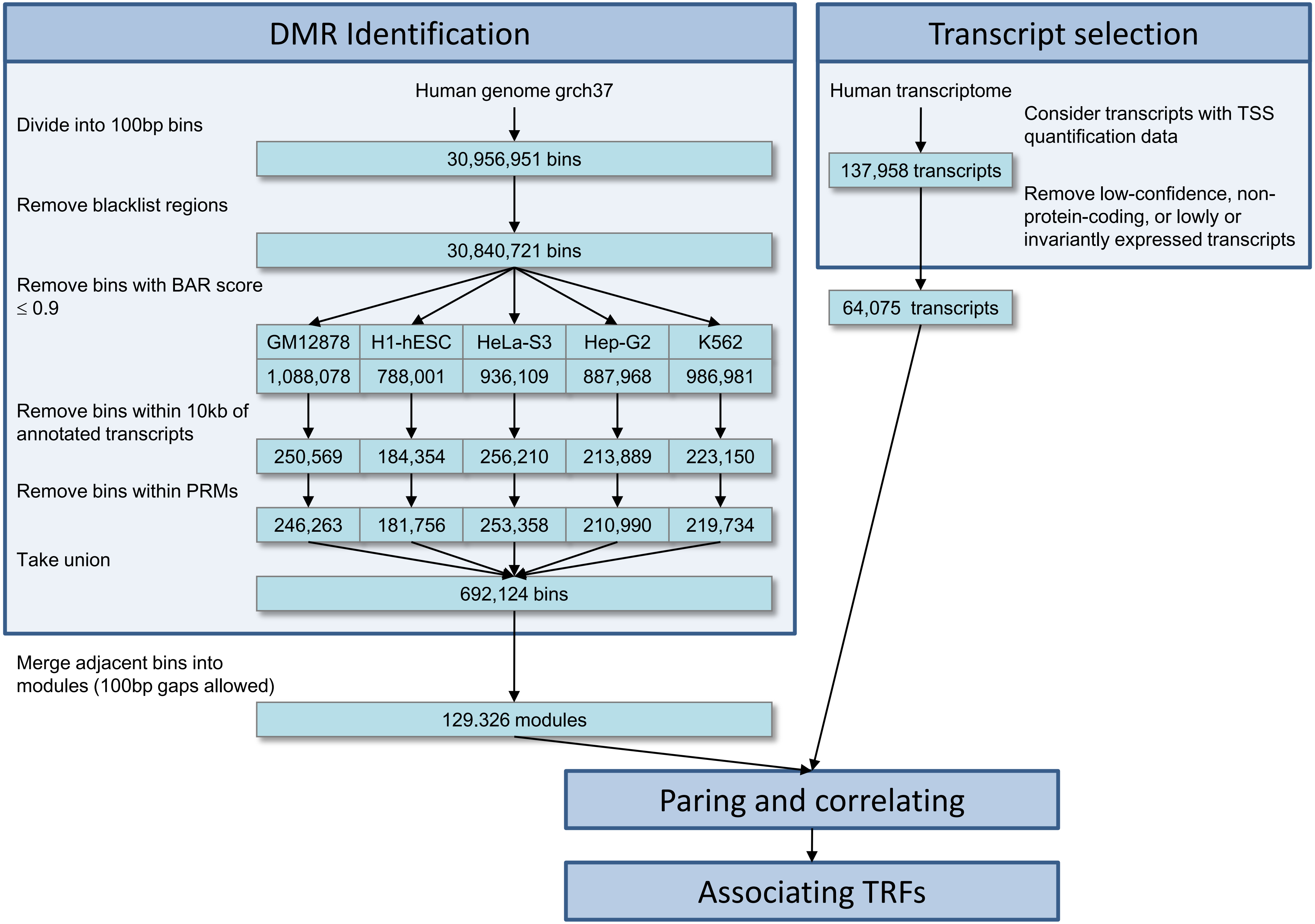

DMR Identification
Human genome grch37
Divide into 100bp bins
30,956,951 bins
Remove blacklist regions
30,840,721 bins
Remove bins with BAR score ≤ 0.9
GM12878
H1-hESC
HeLa-S3
Hep-G2
K562
1,088,078
788,001
936,109
887,968
986,981
Remove bins within 10kb of annotated transcripts
250,569
184,354
256,210
213,889
223,150
Remove bins within PRMs
246,263
181,756
253,358
210,990
219,734
Take union
692,124 bins
Merge adjacent bins into modules (100bp gaps allowed)
129.326 modules
Transcript selection
Human transcriptome
Consider transcripts with TSS quantification data
137,958 transcripts
Remove low-confidence, non-protein-coding, or lowly or invariantly expressed transcripts
64,075 transcripts
Paring and correlating
Associating TRFs

Figure S10B

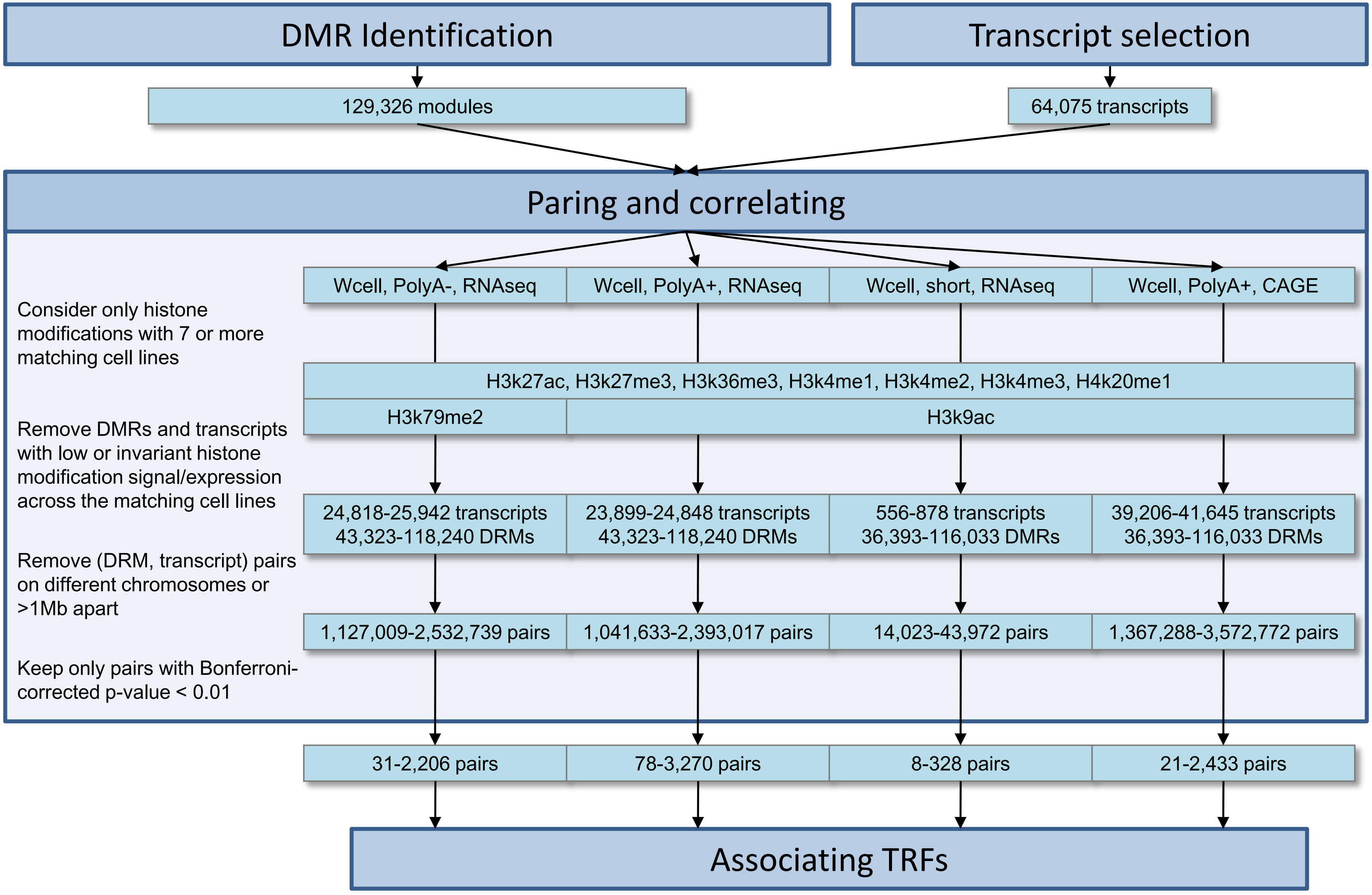
DMR Identification
Transcript selection
129,326 modules
64,075 transcripts
Paring and correlating
Consider only histone modifications with 7 or more matching cell lines
Wcell, PolyA-, RNAseq
Wcell, PolyA+, RNAseq
Wcell, short, RNAseq
Wcell, PolyA+, CAGE
H3k27ac, H3k27me3, H3k36me3, H3k4me1, H3k4me2, H3k4me3, H4k20me1
H3k79me2
H3k9ac
Remove DMRs and transcripts with low or invariant histone modification signal/expression across the matching cell lines
24,818-25,942 transcripts
43,323-118,240 DRMs
23,899-24,848 transcripts
43,323-118,240 DRMs
556-878 transcripts
36,393-116,033 DMRs
39,206-41,645 transcripts
36,393-116,033 DRMs
Remove (DRM, transcript) pairs on different chromosomes or >1Mb apart
1,127,009-2,532,739 pairs
1,041,633-2,393,017 pairs
14,023-43,972 pairs
1,367,288-3,572,772 pairs
Keep only pairs with Bonferroni-corrected p-value < 0.01
31-2,206 pairs
78-3,270 pairs
8-328 pairs
21-2,433 pairs
Associating TRFs

Figure S10C

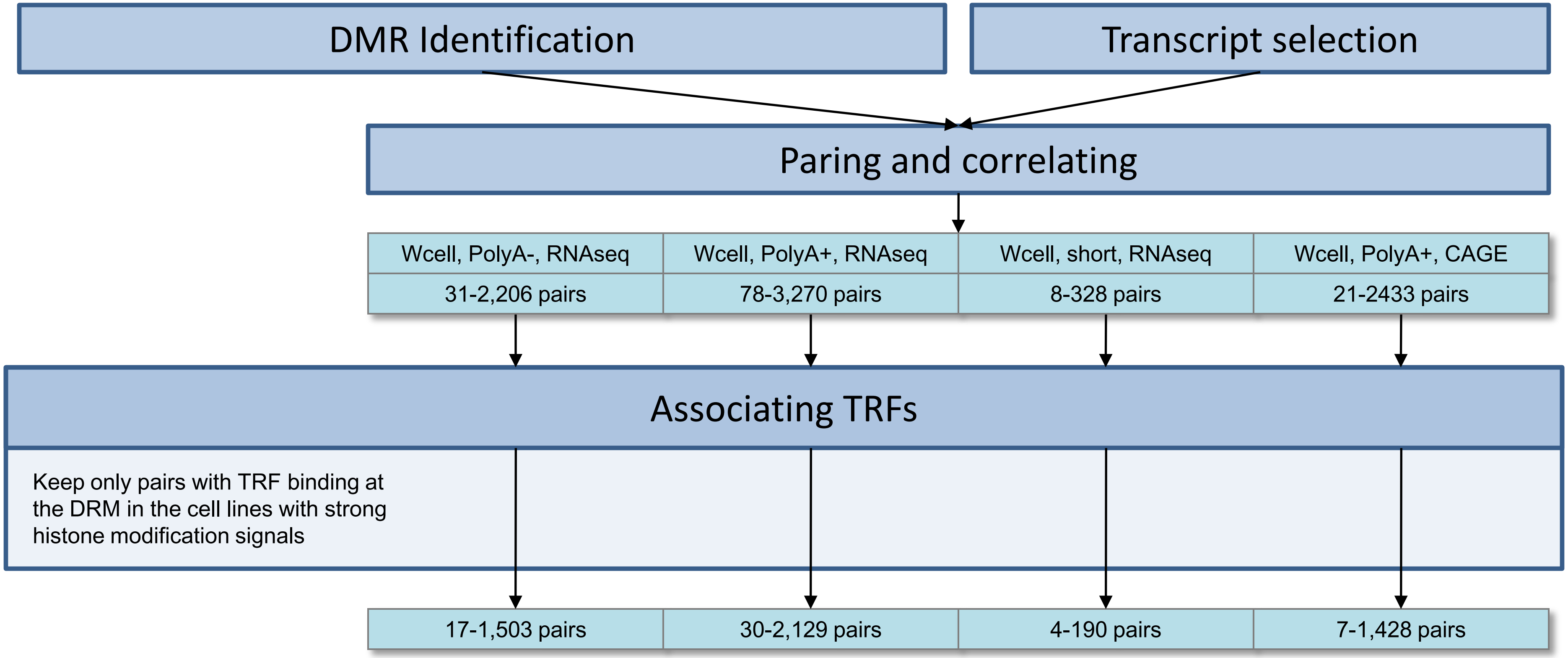

DMR Identification
Transcript selection
Paring and correlating
Wcell, PolyA-, RNAseq
31-2,206 pairs
Wcell, PolyA+, RNAseq
78-3,270 pairs
Wcell, short, RNAseq
8-328 pairs
Wcell, PolyA+, CAGE
21-2433 pairs
Associating TRFs
Keep only pairs with TRF binding at the DRM in the cell lines with strong histone modification signals
17-1,503 pairs
30-2,129 pairs
4-190 pairs
7-1,428 pairs
